# The Complex Admixture History and Recent Southern Origins of Siberian Populations

**DOI:** 10.1093/molbev/msw055

**Published:** 2016-03-18

**Authors:** Irina Pugach, Rostislav Matveev, Viktor Spitsyn, Sergey Makarov, Innokentiy Novgorodov, Vladimir Osakovsky, Mark Stoneking, Brigitte Pakendorf

**Affiliations:** ^1^Department of Evolutionary Genetics, Max Planck Institute for Evolutionary Anthropology, Leipzig, Germany; ^2^Max Planck Institute for Mathematics in the Sciences, Leipzig, Germany; ^3^Research Centre for Medical Genetics, Federal State Budgetary Institution, Moscow, Russian Federation; ^4^Institute of Foreign Philology and Regional Studies, North-Eastern Federal University, Yakutsk, Russian Federation; ^5^Institute of Health, North-Eastern Federal University, Yakutsk, Russian Federation; ^6^Laboratoire Dynamique du Langage, UMR5596, CNRS and Université Lyon Lumière 2, Lyon, France

**Keywords:** multiple admixture events, admixture dating, wavelets, LGM, population replacement, back migration

## Abstract

Although Siberia was inhabited by modern humans at an early stage, there is still debate over whether it remained habitable during the extreme cold of the Last Glacial Maximum or whether it was subsequently repopulated by peoples with recent shared ancestry. Previous studies of the genetic history of Siberian populations were hampered by the extensive admixture that appears to have taken place among these populations, because commonly used methods assume a tree-like population history and at most single admixture events. Here we analyze geogenetic maps and use other approaches to distinguish the effects of shared ancestry from prehistoric migrations and contact, and develop a new method based on the covariance of ancestry components, to investigate the potentially complex admixture history. We furthermore adapt a previously devised method of admixture dating for use with multiple events of gene flow, and apply these methods to whole-genome genotype data from over 500 individuals belonging to 20 different Siberian ethnolinguistic groups. The results of these analyses indicate that there have been multiple layers of admixture detectable in most of the Siberian populations, with considerable differences in the admixture histories of individual populations. Furthermore, most of the populations of Siberia included here, even those settled far to the north, appear to have a southern origin, with the northward expansions of different populations possibly being driven partly by the advent of pastoralism, especially reindeer domestication. These newly developed methods to analyze multiple admixture events should aid in the investigation of similarly complex population histories elsewhere.

## Introduction

Siberia is an extensive geographical region of North Asia stretching from the Ural Mountains in the west to the Pacific Ocean in the east, and from the Arctic Ocean in the north to the Kazakh and Mongolian steppes in the south (supplementary fig. S1, Supplementary Material online). This vast territory is inhabited by a relatively small number of indigenous peoples, with most populations numbering only in the hundreds or few thousands. These indigenous peoples speak a variety of languages belonging to the Turkic, Tungusic, Mongolic, Uralic, Yeniseic, Chukotko-Kamchatkan, and Aleut-Yupik-Inuit families as well as a few isolates. There is also variation in traditional subsistence patterns: The populations of southern Siberia are cattle and horse pastoralists; those of the Amur region focus mainly on fishing, hunting, and gathering; the peoples of central and northern Siberia frequently practice reindeer herding in addition to hunting; and along the coast of the Chukotka and Kamchatka peninsulas the Yupik, Chukchi, and Koryaks are sea mammal hunters and fishers of salmon (Comrie 1981; Evstigneev 2003; Pakendorf 2010). This linguistic and cultural diversity suggests potentially different origins and historical trajectories of the Siberian peoples and warrants further investigation.

The archaeological record attests to the ancient settlement of Siberia by modern humans. In particular, in the Altai-Sayan Mountains and the Lake Baikal region of South Siberia there is ample evidence of a long history of human occupation that highlights the important role South Siberia has played as a gateway into northeastern Asia and the New World. Anatomically modern humans were present in western and southern Siberia from as early as 46 kya (Derevianko et al. 2000; Vasil’ev et al. 2002; Derevianko et al. 2005; Fu et al. 2014). In the Altai region they seem to have overlapped in time and might have coexisted with Neanderthals (Krause et al. 2007; Prüfer et al. 2014) and Denisovans (Krause et al. 2010; Reich et al. 2010). The expansion of humans north was also rapid, and initial occupation of the Arctic environments is evident at more than 35 kya in the European part of the Russian Arctic (Svendsen and Pavlov 2003); by 27 kya humans are already in the Siberian northeast well above the Arctic circle (Pitulko et al. 2004; Nikolskiy and Pitulko 2013), at a time when the Bering Land Bridge was still open (Hoffecker et al. 2014).

An ongoing debate is centered on the degree to which human populations in Siberia were affected by the prolonged cold of the Last Glacial Maximum (LGM) 20-18 kya. Whereas some interpret the archaeological evidence as suggesting continued uninterrupted human occupation throughout the LGM (Fiedel and Kuzmin 2007; Kuzmin 2008; Kuzmin and Keates 2013), probably in protected locations with a milder microclimate, others argue that the extremely cold and arid conditions of the LGM led to a complete depopulation of Southern and Central Siberia (Goebel et al. 2000; Goebel 2008; Graf 2009; Chlachula 2011). It has been proposed that recolonization took place only when the climatic conditions improved after 18 kya, with new microblade stone flaking technologies appearing at this time in the archaeological record. Some researchers argue that these new technologies were brought to the area by immigrating people (Goebel 2008), while others view this change in technology as a gradual in situ transition (Derev’anko et al. 1998). The genome sequence of the 24,000-year-old individual from the Mal'ta site in southern Siberia reveals no genetic affinity between this Upper Paleolithic Siberian and modern human populations of southern and central Siberia, arguing for post-LGM population replacement, while intriguingly also revealing a genetic proximity to present-day Native Americans (Raghavan et al. 2014). Evidence that the population replacement may have taken place at a much later stage (7,000-6,000 ya), however, comes from mitochondrial DNA (mtDNA) analyses from two Neolithic cemeteries from Lake Baikal which are separated in time by an ∼800-year-long hiatus in settlement. While the prehiatus population shows affinities with western Eurasians, the posthiatus population is genetically similar to modern-day populations of Southern and Central Siberia (Mooder et al. 2006).

Despite Siberia's evident importance for understanding the peopling of North Asia and the New World, there are few genetic studies which focus specifically on the history of the Siberian populations, and these are mainly confined to mitochondrial DNA (mtDNA) and Y chromosome markers (Volodko et al. 2008; Malyarchuk et al. 2011; Derenko et al. 2012; Dulik et al. 2012; Malyarchuk et al. 2012; Sukernik et al. 2012; Duggan et al. 2013; Fedorova et al. 2013; Dryomov et al. 2015). Previous studies of ancient mtDNA and Y chromosome single nucleotide polymorphisms (SNPs) and modern uniparental markers highlighted the complexity of South Siberian history and the important role it has played in the peopling of North Asia and the Americas (Derenko et al. 2003, 2010, 2014; Starikovskaya et al. 2005; Keyser et al. 2008, 2009; Dulik et al. 2012; González-Ruiz et al. 2012). In particular, the mtDNA pool of South Siberians is highly diverse (Derenko et al. 2003 , 2010 , 2014), with different lineages tracing their ancestry to the Bronze and Iron Age periods (Keyser et al. 2008 , 2009; González-Ruiz et al. 2012; Derenko et al. 2014) as well as to recent times (Derenko et al. 2003). Furthermore, the mtDNA haplogroups found in South Siberia are shared with other linguistically and culturally unrelated populations as far distant as northern and northeastern Siberia (Starikovskaya et al. 2005; Pakendorf et al. 2006; Volodko et al. 2008; Derenko et al. 2010 , 2012; Dulik et al. 2012; Sukernik et al. 2012; Duggan et al. 2013; Fedorova et al. 2013; Dryomov et al. 2015). The populations of the Amur region, Kamchatka, and Chukotka, however, show distinct mtDNA lineages that testify to a separate history with partial links to the New World (Schurr et al. 1999; Volodko et al. 2008; Sukernik et al. 2012; Duggan et al. 2013). Only three recent studies have analyzed genome-wide data: One focusing on the population history of peoples of northeastern Siberia (Fedorova et al. 2013) and the other two focusing on selection involved in cold adaptation by Siberian populations (Cardona et al. 2014) and in adaptation to the meat-rich diet in populations of the Chukotka peninsula (Clemente et al. 2014). These showed genetic affinities of the populations of Central Siberia with those of Southern rather than Northeastern Siberia, a signal of postcolonial European admixture in the populations of Central and Northeastern Siberia, and regionally and even population-specific signals of selection.

In this study, we compiled a comprehensive data set of genome-wide SNP data from 20 Siberian and 9 reference populations (supplementary table S1, Supplementary Material online) to investigate the relationships among the indigenous populations of Siberia. The assembled data set includes new as well as previously published data (Li et al. 2008; Rasmussen et al. 2010; Reich et al. 2012) totaling 542 individuals, and covers nearly all of the indigenous language families and isolates represented in the region: Turkic, Mongolic, Tungusic, Uralic, Chukotko-Kamchatkan, Aleut-Yupik-Inuit, Yeniseic, and the isolate Yukaghir languages (which might be distantly related to Uralic; Fortescue 1998 and references therein). The aim of our study is to provide for a better understanding of the genetic interactions between Siberian peoples, with a focus on disentangling shared ancestry from prehistoric migrations and contact. To unravel this complexity we use previously published as well as new approaches; in particular, we introduce a new method for determining the order in which two or more admixture events occurred, which we refer to as an Admixture History Graph (AHG), and we modify a previous method for dating admixture (Pugach et al. 2011) for use in more complex admixture scenarios. These new methods, when combined with other approaches (in particular, sharing of blocks of DNA that are identical by descent (IBD) within and between populations and explicitly testing isolation-by-distance against admixture in a model that includes spatial information), provide insights into the complex admixture history of Siberian populations.

## New Approaches

**Fig. 1:**
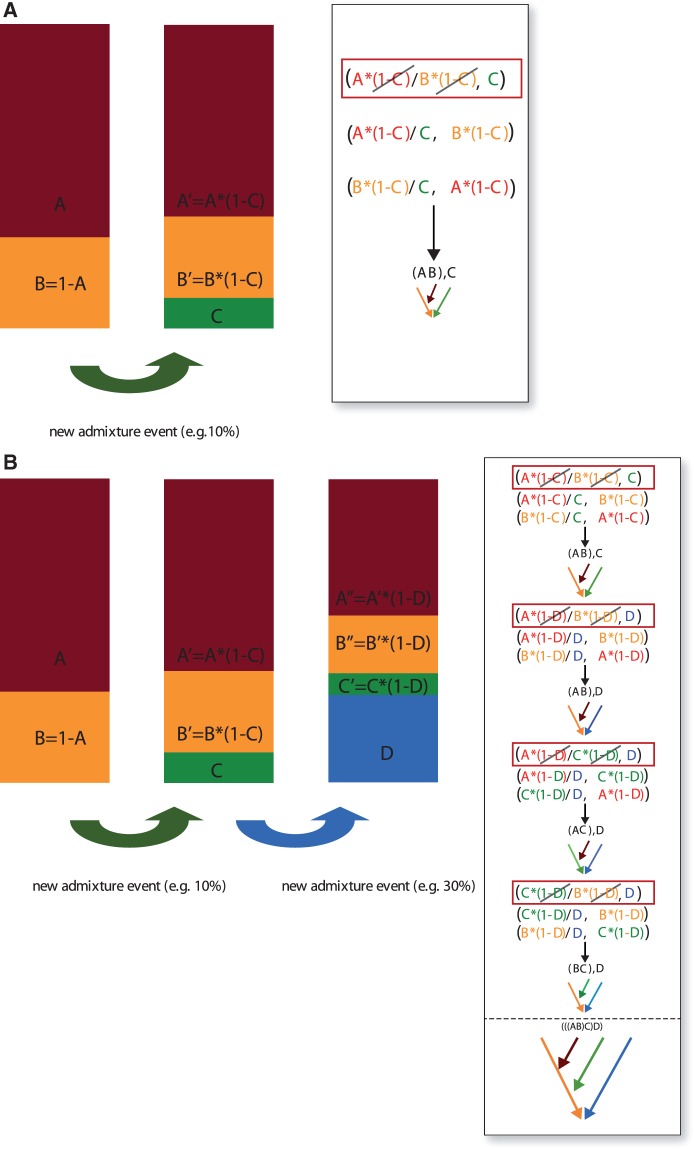
Schematic representation of the AHG approach. (*A*) When an already admixed population with ancestry coming from two different sources A and B experiences another episode of admixture, which brings into this population a new ancestry component C, the proportion of ancestry A and B in each individual in this population becomes adjusted by an amount which depends on the proportion of the component C in the individual. Although before the admixture episode the proportion of A and B among the individuals in the population varied independently of each other, after the new admixture episode A and B start to covary because due to the influx of C each of them has been adjusted by the same amount. The test is based on the detection of this covariance between A and B, as is further illustrated in the inset. (*B*) An example involving additional gene flow from ancestry D.

To disentangle the admixture chronology in populations with complex admixture histories, we developed a new approach referred to here as the AHG. The approach is based on the following idea: If we consider an admixed population with only two sources of ancestry from parental populations A and B, the contributions of A and B among the individuals in the population vary. When a second admixture event occurs, bringing into the population new ancestry C, the proportion of ancestry A and B in each individual becomes adjusted by an amount which depends on the proportion of the component C in the individual (fig. 1A). This means that the distribution of the first two ancestry components A and B across individuals in the new population will covary with C, yet the ratio of A and B throughout the population will be independent from C. Thus the covariance of the recent ancestry C and the ratio of the two older ancestries A and B should be zero. To determine the sequence of admixture events we can therefore simply compare all possible orderings of the three ancestries A, B, and C and choose the one which produces the least absolute value of covariance. The same reasoning applies for populations that have experienced more than two admixture episodes (fig. 1B). In such situations, we test the configuration of all possible trios of A, B, C, and D, and then reconstruct the full graph based on the orderings of likely configurations.

The performance of this new approach was tested with simulated data. In general, the order of admixture events was identified correctly by the algorithm and with great precision for a wide variety of scenarios and even for admixture events involving four sources of ancestry and for small sample sizes (supplementary fig. S2, Supplementary Material online). The test only becomes unreliable when the variance around the mean of all ancestry components in the sample is approximately the same, in which case any existing covariance between the components is obscured. In addition, if the final admixture proportion of one or more components in the population under study is very low (below 5%), the accuracy of the method decreases (see supplementary fig. S2*A* and text S1*B*, Supplementary Material online, for details).

Once the sequence of admixture events is determined by the AHG approach, we use wavelet transform (WT) analysis (Pugach et al. 2011) to estimate the dates of admixture. Since the first version of the WT admixture dating method (Pugach et al. 2011) is restricted to admixture events involving only two parental populations, we have modified the approach here, whereby we consider and date the most recent admixture episode first, and then mask the blocks of most recent ancestry to analyze the signal of admixture in the remaining part of the genome (see Materials and Methods, and supplementary text S1*D*, Supplementary Material online, for the validation of the method).

## Results and Discussion

### The Structure of Genetic Variation in Siberia

**Fig. 2:**
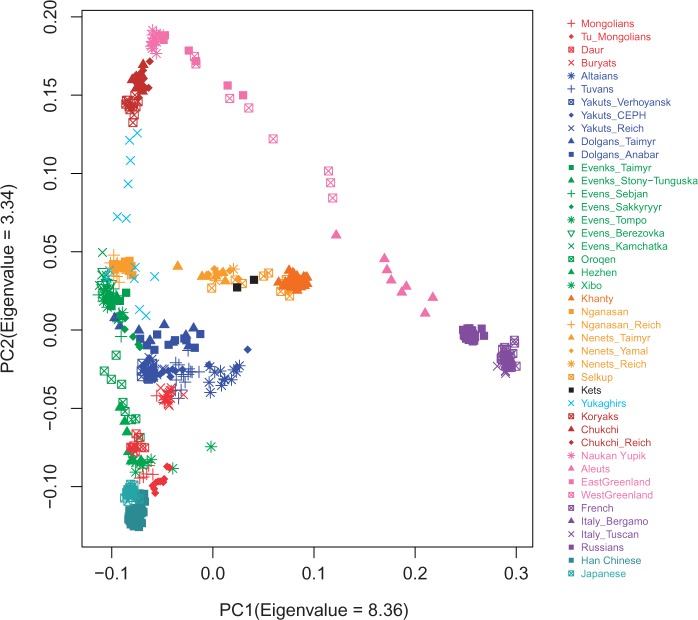
Results of the PC analysis showing the genetic structure captured by the first two principal components. Each colored label represents an individual, and individuals are colored according to their linguistic affiliation as follows: red = Mongolic, blue = Turkic, dark green = North Tungusic, light green = South Tungusic (Hezhen) and Manchu (Xibo), brown = Ugric, orange = Samoyedic, black = Yenisseic, azure = Yukaghirs, maroon = Chukotko-Kamchatkan, pink = Aleut-Yupik-Inuit, purple = Indo-European, teal = Sino-Tibetan and Japonic.

To understand the general patterns of relatedness between the samples in the data set, we started with two widely used exploratory tools: principal components analysis (PCA) (as implemented in StepPCO; Pugach et al. 2011) and the model-based clustering algorithm ADMIXTURE (Alexander et al. 2009). The first principal axis (PC) is driven by differences between Europe and Asia, while the second PC differentiates the northeasternmost populations of the Russian Far East (Chukchi, Koryaks, and Naukan Yupik) and the Inuit of Greenland (fig. 2). Although some populations fall where expected geographically and/or linguistically, in several cases the localization of populations in the PC space is unexpected given their present-day area of settlement or their linguistic affiliation. Thus, the Mongolic populations (color coded in red) fall close to Han Chinese and Japanese, except for the Buryats, who show closer affinities to the Turkic-speaking groups (color coded in blue) than to other Mongolian populations. The Turkic-speaking groups of South Siberia (Altaians and Tuvans) and of Central and Northern Siberia (Yakuts and Dolgans, respectively) fall close together in the PC space, despite the large geographic distances that separate these populations. The Tungusic-speaking Evens and Evenks (color coded in dark green), which were sampled all across central and eastern Siberia, cluster together and overlap with each other in the PC space. In contrast, the Oroqen, an ethnic minority group in northern China who are linguistically closely related to the Evenks (Whaley et al. 1999), form a cline between the Tungusic peoples of Siberia and Han Chinese together with the other Tungusic-speaking minorities of northern China (Hezhen and Xibo; color coded in light green). The Samoyedic-speaking Nganasan, who live on the Taimyr Peninsula in north Siberia (supplementary fig. S1, Supplementary Material online), fall closer to the Evens and Evenks than to their linguistic relatives. Further PCs and the results of the PCA on a subset of the data set are shown in supplementary figures S3 and S4, Supplementary Material online, respectively.

**Fig. 3:**
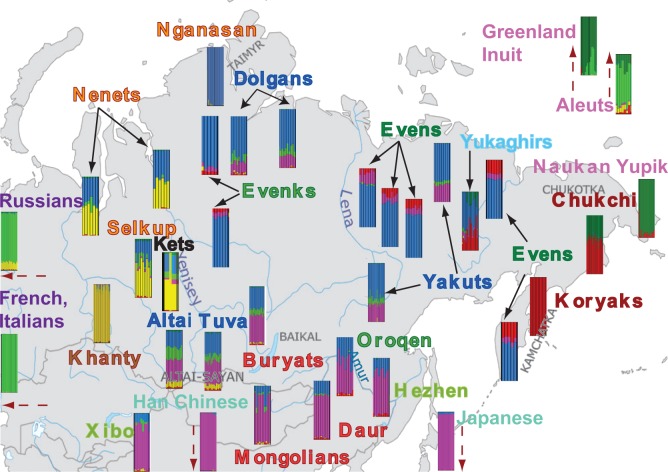
Admixture results for *K* = 6 showing the approximate location of the populations included in this study. The names of the populations are colored according to their linguistic affiliation as described in the legend to figure 2. Where two subgroups are from the same geographic location, only one of the subgroups is shown (full results are presented in supplementary fig. S5, Supplementary Material online). Note that for reasons of space the location of the two distinct Yakut subgroups does not correspond to their true location (which can be seen in supplementary fig. S1, Supplementary Material online). Each color indicates a different ancestry component referred to in the text as European, Western Siberian, Central Siberian, East Asian, Far Eastern, and Yupik-Inuit. Note that the assignment of geographic or population group labels to the genetic ancestry components should not be taken to mean that these populations constituted the actual source populations for the admixture events.

In order to investigate the patterns of potential gene flow, we estimated individual ancestry components using ADMIXTURE (Alexander et al. 2009). Results for *K* = 6 are reported in figure 3 (see supplementary fig. S5, Supplementary Material online, for the results obtained for *K* = 3 through *K* = 7). Although the lowest cross-validation error is obtained for *K* = 7 (supplementary fig. S6, Supplementary Material online), the error obtained for *K* = 6 is also very low, and the standard deviations of these two values overlap. At *K* = 7, the Yakuts are differentiated from the other Siberian populations (supplementary fig. S5, Supplementary Material online), while at *K* = 6, Yakut ancestry is characterized in terms of components found in neighboring groups, and hence these results are more valuable for understanding the prehistory of the Yakuts. Therefore, all AHG analyses to reconstruct the order of admixture events (see Materials and Methods) and admixture dates reported here are based on the results for *K* = 6. The six main components identified in this analysis can be roughly ascribed to Europe, East Asia, Western Siberia (present at highest frequency in the Khanty), Yupik-Inuit (present at highest frequency in the Naukan Yupik), Far East (Koryaks and Chukchi), and Central Siberia (found at highest frequency in the Nganasan).

Strikingly, this analysis paints a very complex picture, revealing that most of the Siberians trace their ancestry to multiple potential ancestral sources. These results suggest that admixture has been important in shaping the history of Siberia. However, some of the observed variations seem to be of a clinal nature and follow a geographical gradient. For example, the Central Siberian component (blue) is observed at highest frequency in the north, and decreases progressively southward. The opposite is true of the East Asian component (pink), while the Far Eastern component (red) seems to follow an east to west cline. While such clinal patterns could be indicative of admixture, they could also be explained by isolation-by-distance processes (Novembre and Stephens 2008). Hence, understanding and disentangling signals of common ancestry and divergence under isolation-by-distance from signals of admixture are key to understanding the history of Siberia, and are the main focus of this study.

Because PCA and ADMIXTURE are descriptive methods, and their results do not necessarily reflect admixture, we first applied two widely used analyses—the 3-population test (f3) (Patterson et al. 2012) and TreeMix (Pickrell and Pritchard 2012) to formally test for admixture. The f3 results (supplementary table S2 and text S1*A*, Supplementary Material online) confirm many, but not all, of the putative signals of admixture suggested by the PCA and ADMIXTURE analyses. TreeMix results were not easy to interpret and seem to contradict well-accepted aspects of human population history (supplementary fig. S7 and text S1*A*, Supplementary Material online). The overall complexity and contradictory signals from the f3 tests and TreeMix could be explained by the putative complexity of the Siberian population history, which potentially includes recent shared ancestry, a multilayered history of admixture, and bottlenecks. These features of Siberian population dynamics violate the underlying assumptions of these methods, i.e., instantaneous admixture at single time points and tree-like population history (Patterson et al. 2012; Pickrell and Pritchard 2012).

**Fig. 4:**
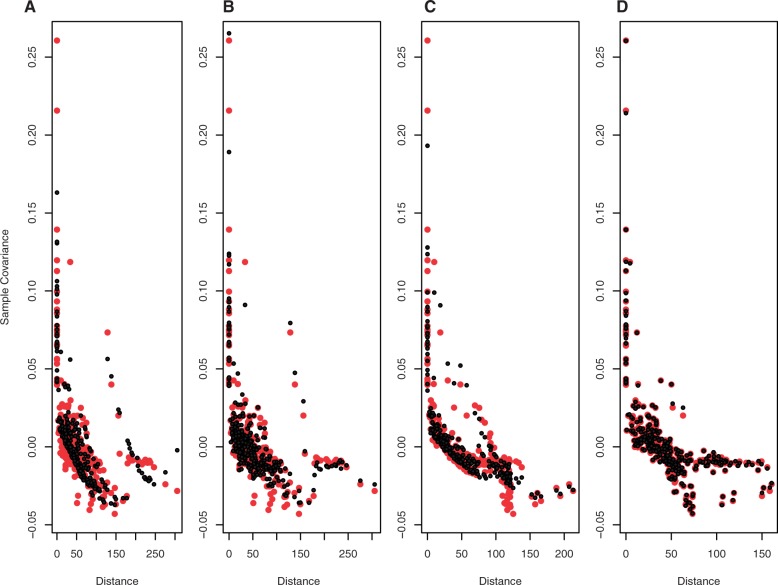
Assessing the fit of the models tested by the SpaceMix software. Each panel shows the decay in sample covariance as a function of observed geographic (red) and inferred geogenetic distance (black). (*A*) No Movement Model: Populations do not choose their own locations, nor can they draw admixture, that is, no migration and no admixture. (*B*) Source Model: Populations do not choose their own locations, but they do draw admixture, that is, admixture without long-distance migration. (*C*) Target Model: Populations choose their own locations, but no admixture, that is, migration without admixture. (*D*) Source and Target Model: Populations choose their own locations and they draw admixture, that is, migration and admixture.

To test if isolation-by-distance alone can account for all of the observed genetic variation in Siberia, we applied the recently developed SpaceMix software (Bradburd et al. 2016). This program uses a Bayesian framework to represent genetic relationships between populations as a “geogenetic” map, in which the distances are proportional to genetic differentiation between the sampled populations. These geogenetic maps are expected to accurately capture the geographic distribution of samples under a model of pure isolation-by-distance; discrepancies between the geogenetic and geographic maps arise due to long-distance migration (resulting in larger than expected geogenetic distances) or admixture (resulting in lower than expected geogenetic distances). We applied SpaceMix to the Siberian data set and found that although the geogenetic map without admixture roughly captures the general geographical distribution of the populations (supplementary fig. S8*A*, Supplementary Material online), there are distortions that are improved by allowing admixture (supplementary fig. S8*B*, Supplementary Material online), and overall the model which incorporates migration and admixture is preferred over a simple isolation-by-distance scenario (fig. 4). In particular, some populations that are separated by large geographic distances exhibit striking signs of relatedness (e.g., Oroqen and Evens; Yakuts and Dolgans with South Siberian Turkic speakers), while others who live in close geographic proximity are genetically differentiated (e.g., Nganasan and Nenets). The admixture proportions inferred by SpaceMix further demonstrate the high amount of admixture that all the Siberian populations have experienced (supplementary fig. S9*A*, Supplementary Material online), while the relatively low level of population-specific drift (variance specific to a population that is not accounted for by the spatial model) shows that the Siberian populations are closely related and share large amounts of population history (supplementary fig. S9*B*, Supplementary Material online). SpaceMix also identifies sources for the inferred admixture events; however, it allows for only a single admixture event per population (Bradburd et al. 2016). As most Siberian populations show evidence of complex multilayered gene flow from distinct geographic locations (fig. 3 and supplementary fig. S5, Supplementary Material online), the sources inferred by SpaceMix were not easily interpretable and are therefore not shown.

To better understand the patterns of relatedness of the Siberian populations and to elucidate to what extent the observed structure can be explained by recent local migrations, we inferred and analyzed chunks of DNA that were inherited without recombination by each pair of individuals from their most recent common ancestor, that is, segments that are identical by descent (IBD) (Powell et al. 2010; Ralph and Coop 2013). We expect individuals to share the highest number of, and longer, IBD segments with other individuals from the same population, and then (in the absence of long-range dispersals) with individuals from geographically close populations. In addition, we can use information on the amount of sharing within each population in comparison to other populations as indirect evidence of past population size changes, because the genomes of individuals in a population that has experienced a bottleneck have shallower genealogies, and hence are expected to share more IBD segments (Gusev et al. 2012), whereas sharing of few IBD segments is a pattern commonly seen in nonisolated populations (Harris and Nielsen 2013; Ralph and Coop 2013).

In terms of within- (and not between-) population IBD sharing, in general the individuals from the populations that now reside in the extreme North or the Far East (e.g., the Naukan and Chukchi) share more IBD blocks with individuals from the same population than do individuals from populations with a more central location, such as the Altaians and the Tuvans (supplementary fig. S10, Supplementary Material online). This result is corroborated by the pattern of genome-wide linkage disequilibrium (LD), where the Koryaks and the Nganasan (populations from the Kamchatka and the Taimyr peninsulas, respectively) exhibit much higher genome-wide LD than that observed for the Han Chinese or Europeans (supplementary fig. S11*A*, Supplementary Material online). This indicates that these populations living on the margins of the Siberian landmass likely experienced an extreme or several bottlenecks, possibly during successive migrations further north- and northeastwards.

**Fig. 5:**
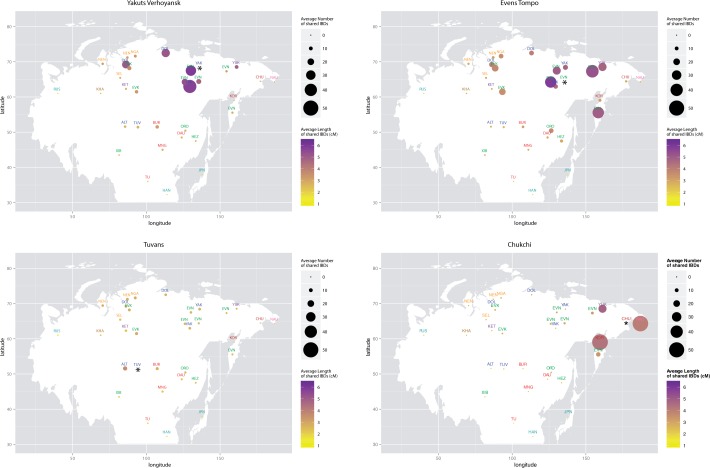
Recent relatedness as measured based on IBD blocks. Each data point represents the results for the comparison of the population marked with an asterisk to each of the other populations in the data set. Data points are placed on the map according to the sampling location of each population (geographic coordinates are listed in supplementary table S1, Supplementary Material online). Population labels are abbreviated to the first three letters of the population name, except EVN = Even; EVK = Evenk; MNG = Mongolian; JPN = Japanese. Each label is color coded according to the population’s linguistic affiliation as described in the legend to figure 2. The size of each circle is proportional to the mean number of IBD segments shared between the population marked with an asterisk and the population named in the label. The color intensity is proportional to the mean length of such shared IBD segments. Plots for the other populations can be found in supplementary fig. S12, Supplementary Material online.

With a few exceptions, the sharing of IBD blocks across Siberia is better explained by geographic proximity of the populations rather than by their linguistic affiliation (fig. 5 and supplementary fig. S12, Supplementary Material online). The most striking exceptions are the Altaians, Tuvans, and Mongolic populations, who share almost no IBD segments with any other population in the data set, and the Evens, who share IBD segments even with geographically distant populations such as the Nganasan and Dolgans from the Taimyr or the Oroqen from North China. In keeping with the SpaceMix results, such patterns of sharing indicate that although isolation-by-distance and recent local migration could explain most of the genetic variation in Siberia, they cannot account for all of the observed diversity. Rather, other demographic processes such as large-scale population dispersals and rapid expansion and/or gene flow (e.g., Evens; Tugolukov 1980; Janhunen 1996; Duggan et al. 2013) as well as language shifts (e.g,. Buryats; Cydendambaev 1981; Buraev and Shagdarov 2004; Čimitdoržieva 2004) are likely to have played a role in the history of some Siberian populations. Furthermore, the signal of recent interactions is completely absent in populations like the Altaians and Tuvans, and non-IBD-based methods are needed to infer demographic events concerning these populations.

**Fig. 6:**
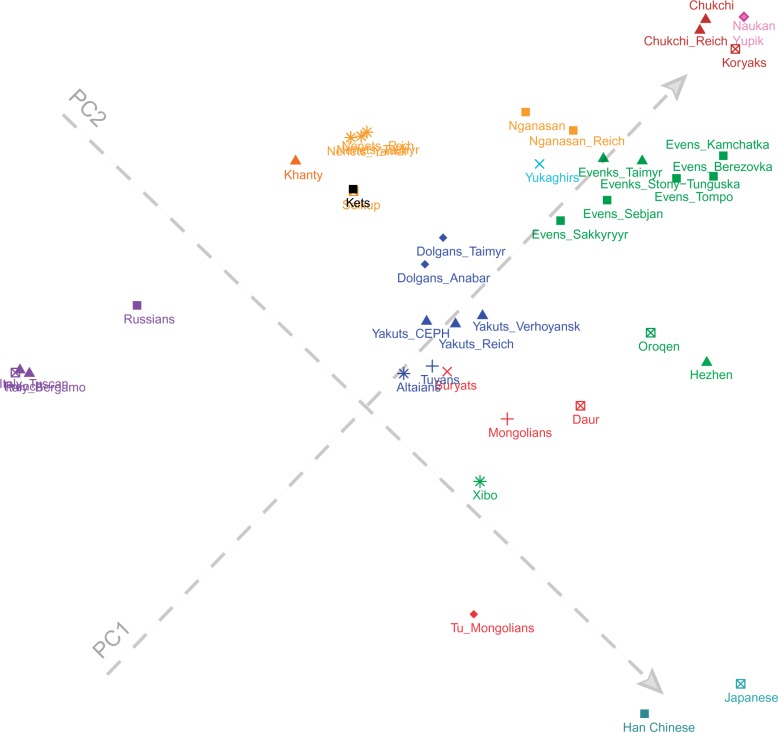
Results of the PC analysis based on the number of IBD blocks shared between populations. Each colored label represents a population, and color coding is according to linguistic affiliation, as described in the legend to figure 2. The PC axes are rotated for a better alignment with the geographic map of Siberia.

It has been previously shown that when PC analysis is applied to human genetic data from some geographic regions, such as Europe, the variation captured by the first two principal axes closely correlates with the geographic coordinates of the actual place of origin of each sample (Lao et al. 2008; Novembre et al. 2008). To further investigate the relationship between the distribution of genetic variation in our data and geography, we ran PC analysis on the inverse of the similarity matrix calculated from the number of shared IBD blocks between populations, as populations sharing more IBD blocks are more closely related genetically. For a better alignment with geography, the coordinate axes were rotated −45° (fig. 6). The main axis of variation is oriented in a northeast–southwest direction, while the second axis separates east and west. Similar to the geogenetic map inferred by SpaceMix (supplementary fig. S8*B*, Supplementary Material online), the PC map inferred from the number of shared IBD blocks reveals that most of the genetic distances between the Siberian populations are smaller than expected based on their actual location on a geographic map (fig. 6). The most notable exceptions are the populations of the Taimyr, who are dispersed on the PC plot, even though they are settled in relatively close proximity to each other. In particular, the Nganasan appear to be much closer to the Tungusic-speaking Evenks and the Yukaghirs than to the neighboring Nenets (cf. Fedorova et al. 2013), even though the Nenets are not only geographically close to the Nganasan but also speak a related language (Janhunen 1998). Similarly, the Mongolic-speaking Buryats cluster with the Turkic-speaking Altaians and Tuvans, and not with the Mongolic-speaking Mongolians and Daurs, although these are linguistically related and geographically less distant.

To further explore how genetic distances between populations are influenced by geography and to what extent the genetic variation in Siberia might be explained by isolation-by-distance, we used multiple regression of genetic distances on geographic distances (Ramachandran et al. 2005; Yunusbayev et al. 2012). Historically, rivers played an important role in the movement of peoples across Siberia. Today, as in the past, when they freeze over in winter these rivers serve as “ice highways,” efficiently connecting far-away settlements and towns. Rivers therefore were also included into our regression model as an additional explanatory variable, as described in the Materials and Methods. This analysis reveals that geographic distance alone explains 48% of the genetic variance (*P* < 0.001). Adding rivers as facilitators of gene flow improves the fit of the observed genetic distances to geography, increasing the Rsq by 0.02 (*P* < 0.001). We then jackknifed over populations by sequentially removing each population and refitting the regression (Ramachandran et al. 2005). The largest improvement to the fit was achieved by removing the Aleuts, Han Chinese, and Japanese samples, with 58% of the variation in genetic distances between populations being explained by geography (*P* < 0.001) (supplementary fig. S13, Supplementary Material online). The general conclusion from this analysis is that geography (i.e., isolation-by-distance) indeed explains a large part of the genetic variance; however, it cannot explain all of the genetic relationships in Siberia. Instead, long-distance migration and admixture are likely to have further shaped the gene pool of Siberian populations.

In summary, our exploratory analyses demonstrate that the prehistory of Siberian populations has been complex, and even though local effects of geographical proximity (isolation-by-distance) do explain much of the genetic diversity in Siberia, large-scale dispersals and variable degrees of admixture must have also played an important role in structuring the genetic variation of these peoples. These analyses also demonstrate that individual populations have had different historical trajectories, with those of the periphery being notably distinct from the others. In the following section, we analyze signals of admixture and discuss the genetic prehistory of the populations of South Siberia, while the analyses pertaining to the other populations are described in supplementary text S3 and figures S14–S17, Supplementary Material online.

### South Siberia (Altaians, Tuvans, and Buryats)

The populations of southern Siberia are mainly pastoralists who speak Turkic (Altaians and Tuvans) or Mongolic languages (Buryats). Evidence for the use of domesticated animals (cattle, sheep, goats, and horses) in this region goes back to the Neolithic (Laufer 1917; Mirov 1945; Vainshtein 1980; Clutton-Brock 1999; Clutton-Brock 2012). The results of the ADMIXTURE analysis for the populations of South Siberia are striking (fig. 3) and reveal the important role played by the Sayan and Altai Mountains in the human history of Siberia: All of the ancestry components found across Siberia are represented here (cf. Cardona et al. 2014), although two (Far East and Yupik-Inuit) are present in too low proportions to permit further analysis. This is consistent with South Siberia's rich archaeological record with a wealth of sites representing different cultures, either contemporaneous or following in quick succession, and attests to its importance at the crossroads of various migration routes (Sinor 1990; Tumen 2008; Mezzavilla et al. 2014), which resulted in an amalgamation of different ancestries (supplementary text S2, Supplementary Material online).

**Fig. 7:**
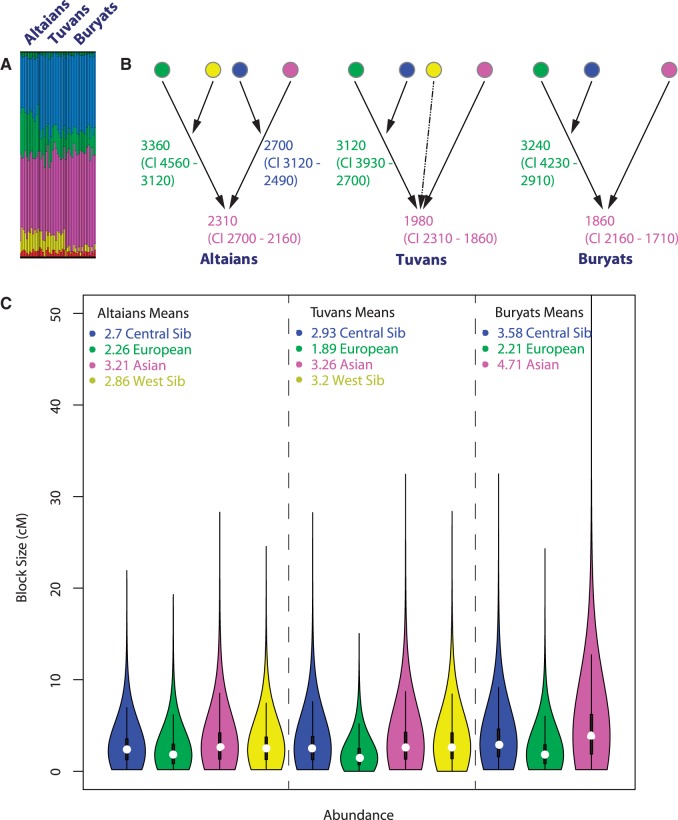
Admixture profiles for populations of South Siberia: Altaians, Tuvans, and Buryats. (*A*) An excerpt from the plot summarizing results of the ADMIXTURE analysis for the Altaians, Tuvans, and Buryats at *K* = 6. The full panel is shown in supplementary figure S5, Supplementary Material online. (*B*) AHGs and admixture dates inferred for each population and each admixture episode. Proxy parental populations for the different ancestral components (represented as circles) were as follows: European (green) = Italians; Western Siberian (yellow) = Khanty; Central Siberian (blue) = Nganasan; East Asian (pink) = Han Chinese. (*C*) Cumulative distribution of all ancestry blocks. For each population the plot captures the total abundance of blocks of each ancestry (*x*-axis) of different genetic lengths in cM (*y*-axis); the average width of the blocks of each ancestry and variance around the mean are also shown.

The ancestry composition in Altaians, Tuvans, and Buryats is almost the same (fig. 7A), even though the proportions of the different ancestry components vary slightly among the populations. The Altaians have a higher amount of the European component (21% vs. ∼10% in Tuvans and Buryats) and a lower amount of the Central Siberian ancestry component (27% vs. 37% in Tuvans and Buryats). The Western Siberian ancestry component occurs at similar frequencies in Altaians and Tuvans (10% and 8%, respectively), while it is almost absent in Buryats (2%). In contrast, the Asian ancestry component is higher in Buryats (47% vs. ∼38% in Altaians and Tuvans).

Despite the overall similarity of the ADMIXTURE results, the AHG analysis (which is consistent across the best 30% of the ADMIXTURE runs) infers different admixture graphs (and hence, admixture histories) for the Altaians as compared to Tuvans and Buryats (fig. 7B). However, it should be noted that there is some uncertainty in the reconstructed sequence of admixture events involving more than three ancestral populations when sample sizes are small and levels of admixture low (supplementary text S1*B*, Supplementary Material online). As both issues are relevant for South Siberia, we performed further tests to validate the different inferred admixture sequences we obtain for the South Siberian populations (see supplementary text S1*C*, Supplementary Material online, for details).

The configuration most supported by the AHG calculation (supplementary table S3, Supplementary Material online) is shown in figure 7B. As can be seen, the admixture history of Altaians differs from that of Tuvans and Buryats (fig. 7B and supplementary table S3, Supplementary Material online) in that the first admixture event in Altaians involved West Siberian and European ancestries, whereas for Tuvans and Buryats the first event involved the European and Central Siberian ancestries. The estimated dates of the admixture events, however, are roughly comparable, with the first event taking place ∼3,000–3,500 ya and the most recent event ∼2,000 ya. Since the average width of blocks of East Asian ancestry and the variance around the mean are larger in Buryats than in Altaians and Tuvans (fig. 7C), it is likely that the Buryats experienced additional gene flow from a source of mainly East Asian ancestry. This would explain why the signal of Asian admixture in the Buryats appears to be younger than the same signal in Altaians and Tuvans; however, it is also possible that this date is underestimated, as it is very close to the resolution limit available for the Buryats (supplementary fig. S18*D*, Supplementary Material online). The AHGs and the dates of the admixture events inferred here for the South Siberian populations are consistent with the archaeological record (see supplementary text S2, Supplementary Material online, for details).

To summarize, although we cannot rule out the possibility that the admixture history in the three South Siberian populations analyzed here was the same, the evidence instead suggests different scenarios for Altaians and Tuvans/Buryats. Since the European ancestry component is involved in the earliest admixture events in all three populations and the average length of European tracts across the South Siberian populations is the smallest, this component cannot reflect postcolonial Russian admixture, in contrast to previous findings (Cardona et al. 2014). Instead, it appears to reflect the oldest population substrate in South Siberia (fig. 7B) and might be related to the spread of the Yamnaya steppe peoples to the Sayan and Altai Mountains in the Bronze Age (Allentoft et al. 2015).

The particular demographic history of the South Siberian populations is further underlined by the LD analysis (supplementary fig. S11*B*, Supplementary Material online): South Siberian populations have consistently higher LD than Europeans, but lower short-range and higher long-range LD than Han Chinese. The short-range LD patterns suggest an intermediate-sized bottleneck in the past in South Siberian populations (Hayes et al. 2003; Tenesa et al. 2007), compared to the stronger bottleneck in Asians and weaker bottleneck in Europeans (Schaffner et al. 2005; Keinan et al. 2007), while the long-range LD patterns suggest more recent gene flow in South Siberian populations (Plagnol and Wall 2006; Jakobsson et al. 2008; DeGiorgio et al. 2009; McEvoy et al. 2011).

It is furthermore notable that the Buryats, who speak a Mongolic language closely related to Khalkha Mongolian, genetically do not pattern with the Mongolians, but with the South Siberian Turkic groups in all respects. This is consistent with the prevailing view that Buryats are the descendants of indigenous populations from Lake Baikal who shifted to their current Mongolic language (Cydendambaev 1981; Buraev and Shagdarov 2004; Čimitdoržieva 2004).

## Conclusions

**Fig. 8:**
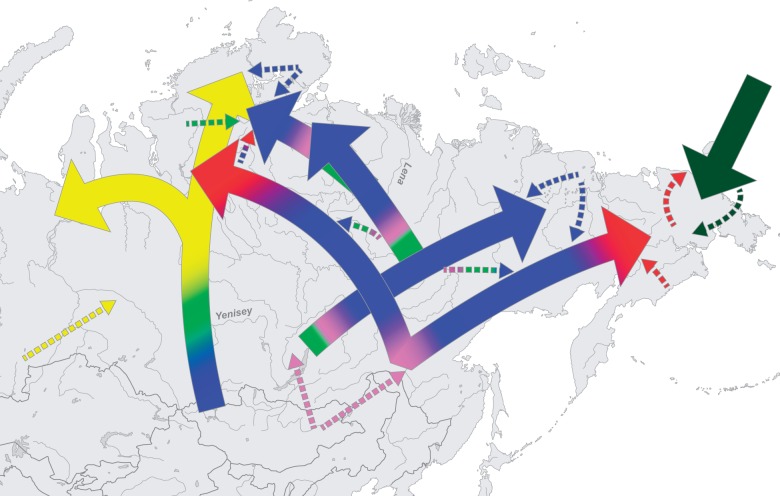
A schematic representation of the suggested migrations and directions of gene flow. This diagram illustrates the main findings from our data, and does not necessarily reflect the true history of the populations.

Figure 8 summarizes and synthesizes the results of all of our analyses in a schematic representation (see supplementary text S3, Supplementary Material online, for the details concerning individual population histories on which this is based). We find that practically all Siberian populations show evidence of extensive and deeply complicated admixture. Admixture events are often reciprocal, with many populations showing signals of multiple independent pulses of gene flow and/or of gene flow occurring over a prolonged period of time. Disentangling the ancestries from many different (often related) sources that mixed at different time points in the past, and often more than once, is a very challenging problem. To unravel this complexity we developed a new method, the AHG, which infers the sequence of admixture events in a population with a complex admixture history. The approach was tested on simulated data, and we show that it is applicable to a wide variety of admixture scenarios (supplementary text S1*B*, Supplementary Material online). The information obtained via the AHG test can then be used to date multiple admixture events involving more than two sources of ancestry. To this end, we adapted and tested with simulated data a previously published admixture dating method (Pugach et al. 2011). As with other such methods (Gravel 2012; Hellenthal et al. 2014), analyzing continuous admixture remains a difficult task. Even though we can identify continuous admixture events by analyzing the distribution of widths of ancestral blocks, the dates inferred for these events do not provide information on the beginning or the end of the gene flow, but show a composite of these two dates instead.

Our results indicate that most Siberian populations share a relatively recent history, with none of the admixture dates detected being older than ∼3,400 years. These recent dates could indicate a limit of the resolution imposed by the data, such as low level of genetic differentiation of the parental groups chosen as proxies for the sources of admixture, insufficient resolution of the SNPs on the Illumina platform, and small Ne of the groups analyzed. However, our simulations (supplementary text S1*F*, Supplementary Material online) suggest that many of the dates we infer are below the data-inherent limit of resolution; therefore, the inferred dates are unlikely to seriously underestimate the true dates of admixture. These recent dates are also indirectly supported by recent studies, which have shown massive bidirectional migrations of peoples across the Eurasian steppe and which suggest major changes in the genetic structure of Eurasia, such as admixture and replacement of autochthonous populations, to have occurred in the Bronze Age (Allentoft et al. 2015; Haak et al. 2015). This result is also in good accordance with evidence of a depopulation of Siberia during the LGM (Raghavan et al. 2014) and with previous suggestions of recent population replacements in this region (Weber et al. 2002; Mooder et al. 2006; Weber and Bettinger 2010). It is still possible however that in South Siberia human groups persisted through the LGM in small refugia (Chlachula 2011; Blomdin et al. 2014; Hais et al. 2015) which later could have been the source for subsequent re-expansions. Whether or not the South Siberian populations could be used as proxies for the ancestors of the populations that settled the New World is a question to be addressed by future studies.

The most extensive admixture has clearly happened in South Siberia, which is also the area to which several of the central and northern Siberian populations trace their origins. Thus, the Yakuts show clear evidence of having originated in an area near Lake Baikal and of stemming from a common ancestral population with the Buryats (Tokarev and Gurvich 1956; Crubézy et al. 2010). The Selkup and Nenets, too, show signs of a southern origin and possibly shared ancestry with the Tuvans. In contrast, the Tungusic-speaking populations are likely to have originated not in the Lake Baikal area, as suggested previously (Vasilevič 1969), but in the Amur region (Tugolukov 1980). Some of these recent expansions from southern to northern Siberia may have been driven by the advent of pastoralism, especially reindeer domestication, which is known in South Siberia from around 3,000 ya (Laufer 1917; Mirov 1945; Vainshtein 1980; Clutton-Brock 1999; Clutton-Brock 2012).

Furthermore, our results demonstrate that the European ancestry component detectable in many populations of southern, central, and northern Siberia is not the result of postcolonial Russian admixture as may have been expected (Fedorova et al. 2013) and as was suggested elsewhere (Cardona et al. 2014). Rather, with the exception of the Dolgans and the Samoyedic-speaking groups of western Siberia, the European ancestry represents one of the most ancient components of the complex admixture history of Siberian populations.

In summary, our new methods for determining the sequence and date of multiple admixture events have enabled us to disentangle the extremely complex patterns of prehistoric admixture among Siberian populations. These methods will also be useful in further investigations of population prehistory and gene flow in other regions of the world.

## Materials and Methods

### Ethics statement

The collection of the samples was approved by the Ethics Committee of the University of Leipzig and the Ethics Committee of the Research Centre for Medical Genetics, Russian Budgetary Federal Institution, Moscow. Written consent to use their samples was obtained from all donors after explanation of the aims of the study.

### Genotypes

We genotyped 96 Siberian samples using Illumina 660W-Quad arrays. Quality filtering was performed as described previously (Pugach et al. 2013). These data were merged with Illumina genotypes from three other studies (Li et al. 2008; Rasmussen et al. 2010; Reich et al. 2012). After quality filtering and data integration, 542 individuals and 353,357 autosomal SNPs remained for analysis. Not all samples were used for all of the analyses.

#### Samples

The data set (supplementary fig. S1 and table S1, Supplementary Material online) includes four different Turkic-speaking groups: Altaians and Tuvans (South Siberia), Yakuts (three groups from the Sakha Republic), and Dolgans (from the Taimyr peninsula and the northwest of the Sakha Republic); four Mongolic-speaking populations (Buryats, Mongolians, Daurs, Tu); four Uralic populations: Samoyedic-speaking Nganasan (Taimyr peninsula), Nenets (sampled on the Taimyr and Yamal peninsulas), and Selkup, as well as the Finno-Ugric Khanty from western Siberia; and Tungusic-speakers from five ethnolinguistic groups representing all the branches of the Tungusic family: Northern (Evens, Evenks, and Oroqen), southern (Hezhen), and Manchu (Xibo). The Even samples come from five and the Evenk samples from two different locations across Siberia. Furthermore, we included Yukaghirs and Kets (who speak an isolate language and the last surviving language of the Yeniseic family, respectively), and populations of Chukotka and Kamchatka: Chukchi, Koryaks (who speak languages of the Chukotko-Kamchatkan family) and Naukan Yupik (who speak a language of the Aleut-Yupik-Inuit family). For comparative purposes we augmented the data set with Aleuts as well as Inuit populations of Greenland, and added European (Russians, French, and Tuscan and Bergamo Italians), Han Chinese, and Japanese populations from the HGDP (for detailed information on the populations and the provenance of the samples, see supplementary table S1, Supplementary Material online).

#### Data Curation

Quality filtering was performed as described previously (Pugach et al. 2013). Twenty-five individuals were removed before analysis: Three due to low genotyping quality (one Nganasan, one Yamal Nenets, and one Taimyr Nenets), one because it was identified as a potential close relative to others (Yamal Nenets), while the others were removed because they were determined via PCA to be outliers relative to their population (one Yamal Nenets, one Taimyr Nenets, one Yakut, four Nganasan, one Altaian, six Koryaks, one Selkup, one Japanese, five Chukchi).

The Yakut, Nganasan, Chukchi, and Tundra Nenets populations from Reich et al. (2012) (supplementary table S1, (Supplementary Material online) were excluded from the IBD, regression, and dating analyses because no geographical coordinates were available for these samples, and because we had other samples from the same populations (supplementary table S1, Supplementary Material online) whose PCA and ADMIXTURE results overlapped with those of the Reich et al. samples (figs. 2 and 3).

Prior to running the AHG calculation, we removed: 1) One individual from the Selkup population, as this sample is an outlier with respect to others in terms of its admixture profile: It is the sample with the highest proportion of Central Siberian and the lowest proportion of European ancestry (45% and 4%, respectively); 2) one Yamal Nenets sample, as for this sample we estimated 28% European admixture (while the population average is 4%); 3) two Xibo samples with no Far Eastern ancestry (present in all other Tungusic speakers) and higher than the population average of European ancestry (19% and 9% vs. population average of 2%); 4) two Taimyr Dolgans with no European ancestry, while this ancestry is present on average at 12% in the remaining Dolgans; 5) in the HGDP sample of Yakuts, European admixture ranges from 4% to 30%, while in the Verkhoyansk Yakuts it ranges from 4% to 7%; therefore to exclude recent Russian admixture, we limited the analysis in the HGDP Yakuts to individuals with European admixture not exceeding 8% (this removed 11 individuals).

Furthermore, PCAdmix runs failed to resolve ancestry for some chromosomes of Altaians and Tuvans (for these populations ancestry was inferred using four source populations). These chromosomes were removed prior to the admixture dating analysis. In total, 66 (out of 528) and 78 (out of 704) haploid chromosomes (the data were phased with BEAGLE, see IBD Segments) were removed from the Altaians and Tuvans, respectively. In addition, for the same reason in the Tuvans chromosome 20 was completely removed prior to dating.

### Statistical Analyses

#### Exploratory Analyses of Genetic Variation

##### PCA

PCA was performed using the StepPCO software (Pugach et al. 2011) on 353,357 markers for the entire data set and on 504,356 markers when analyzing a subset comprising only the Siberian populations.

##### ADMIXTURE

We used the ADMIXTURE software (Alexander et al. 2009) to infer individual ancestry components and admixture proportions; the LD pruning for this analysis was done using the PLINK tool (Purcell et al. 2007) with the following settings: –indep- pairwise 200 25 0.4 (Rasmussen et al. 2011), which reduced the data set to 148,620 markers. We ran ADMIXTURE for *K* = 3 through *K* = 10, and performed ten independent runs for each value of *K*. We confirmed consistency between runs and used the cross-validation procedure implemented in ADMIXTURE to find the best value of *K* (supplementary fig. S6, Supplementary Material online). We ran an additional 90 runs for *K* = 6 (for a total of 100 runs), and used the top 30% of all runs to confirm consistency of the inferred AHGs. We also calculated the standard errors on all admixture estimates for *K* = 6 via bootstrapping as implemented in ADMIXTURE (Alexander et al. 2009). All standard errors are less than 0.025 and are reported in supplementary figure S19, Supplementary Material online.

#### Methods of Demographic Inference

For subsequent analyses, genetic distances between SNPs were interpolated using genetic coordinates and recombination rates estimated as part of the HapMap (International HapMap Consortium et al. 2007) and the 1000 Genomes Projects (1000 Genomes Project Consortium 2010), and obtained using the SNAP tool (Johnson et al. 2008).

##### 3-Population Test and TreeMix

The f3 statistics were calculated with the threepop program implemented in TreeMix. Calculation of allele frequencies for the TreeMix analysis was performed using PLINK tool (Purcell et al. 2007). The San samples were used as an outgroup, and we used the window size of 500 (-k option).

##### IBD Segments

The data were phased using BEAGLE v3.3.2. IBD segments were inferred using the fastIBD method implemented in BEAGLE (Browning SR and Browning BL 2007; Browning BL and Browning SR 2011). As recommended by the developers, we ran the algorithm 10 times with different random seeds. The results were then combined and postprocessed using custom scripts, but following the procedure described previously (Ralph and Coop 2013). In particular, we extracted only those IBDs seen at least twice in the ten BEAGLE runs and kept only those with a significance score lower than 10^9^. To remove gaps introduced artificially because of low marker density or potential switch error, we merged any two segments separated by a gap shorter than at least one of the segments and no more than 5 cM long (Ralph and Coop 2013). For this calculation, individuals were grouped in two ways: According to their population designation and by sampling location. All results were adjusted for differences in sample size, and blocks shorter than 2cM were excluded (Ralph and Coop 2013). To run PCA based on the results of the IBD calculation, we used the ade4 R package (Dray and Dufour 2007); as a distance matrix we used the inverse of the pairwise matrix based on the number of shared IBD blocks (such that two populations sharing the smallest number of IBD blocks would be furthest apart from each other in terms of distance).

##### Multiple Regression

Multiple regression on genetic and geographic distance matrices was performed and the statistical significance of the regression coefficients was determined using the ‘‘ecodist’’ R package (Goslee and Urban 2007). For this, the inverse IBD-sharing matrix was used as a measure of genetic distance between populations. Geographic coordinates for the HGDP populations were taken from Ramachandran et al. (2005), while the sampling locations for the samples introduced in this study were used as recorded at the time of sample collection, with Google Maps used to determine longitude and latitude for these locations. For the populations taken from other studies, where the exact sampling location was unknown, we used the coordinates of a central point of the population's current place of residence (province or region, as listed in supplementary table S1, Supplementary Material online). Geodesic distances were calculated using the distonearth R function (Banerjee 2005; Yunusbayev et al. 2012). Rivers were added as an additional factor into the multiple regression model as putative facilitators of gene flow as follows: The Ob as a facilitator of gene flow between the Yamal Nenets and Khanty; the Yenisei as a facilitator of gene flow between the Taimyr Nenets, Selkup, Kets, Evenks, Tuvans, and Altaians; the Amur as a facilitator of gene flow among Hezhen, Oroqen, and Daurs; the Lena for gene flow between Buryats, Yakuts, and the Even subgroups from Tompo, Sakkyryyr, and Sebjan; and finally the Kolyma as a facilitator of gene flow between Yukaghirs and the Evens from Berezovka. Two populations were assigned zero distance between them if they were connected by a river, and a distance of one if they were not.

##### SpaceMix

SpaceMix analysis was performed with default settings (Bradburd et al. 2016), namely for each tested model we ran 10^5^ Markov chain Monte Carlo iterations of the short chain; a long chain was initiated from the parameter values in the last iteration of the short chain and was run for 10^7^ iterations and sampled every 10^4^ iterations for a total of 1000 draws from the posterior. Geographic coordinates of population locations were used as the prior. The models tested were as follows: 1) Populations do not choose their own locations, nor can they draw admixture (i.e., pure isolation-by-distance); 2) populations are not allowed to choose their own locations, but they do draw admixture (i.e., isolation-by-distance plus admixture), 3) populations choose their own locations, but no admixture (i.e., isolation-by-distance plus migration), and 4) populations choose their own locations and they draw admixture (i.e., isolation-by-distance plus migration plus admixture).

##### Linkage Disequilibrium

Genome-wide LD calculation was performed using custom scripts, as described in Pugach et al. (2013), based on the genetic map downloaded from the Illumina webpage (http://support.illumina.com/array/array_kits/human660w-quad_dna_analysis_kit/downloads.ilmn, last accessed March 31, 2016). In particular, we binned genotype data from each population into 50 evenly spaced recombination distance categories (0.005–0.25 cM). For each population and for every pair of SNPs in each distance category, we calculated the squared correlation in allele frequencies (Rsq) by selecting from each population 11 individuals showing the least amount of admixture (as determined by ADMIXTURE). For this analysis, individuals from different sampling locations of the same population were grouped. To account for missing data, each pairwise measurement was adjusted by sample size.

#### Analyzing and Dating Multiple Admixture Events

To analyze signals of admixture in the Siberian populations involving more than two ancestral populations, we adopted the following workflow: The data were phased with the BEAGLE software (Browning SR and Browning BL 2007), then the PCAdmix tool was used to infer ancestry along individual chromosomes (Brisbin et al. 2012). Unlike other existing methods for ancestry estimation, this algorithm allows for simultaneous inference of admixture from more than two sources of ancestry. In order to determine the sequence of admixture events we developed a new approach, the AHG (see below for details), based on the results of the ADMIXTURE analysis. Finally, we applied our previously published WT analysis (Pugach et al. 2011), adapted to include complex admixture scenarios, to the results obtained from PCAdmix and using the information on the sequence of events obtained from the AHG, to estimate the dates of the different admixture events (see supplementary text S1*D*, Supplementary Material online, for validation). To aid interpretation, we also analyzed the length distribution of blocks of different ancestry, as inferred by PCAdmix.

##### PCAdmix

Our choice of parental populations for the PCAdmix analysis was guided by the results of the ADMIXTURE analysis for *K* = 6, and populations that had been assigned their own ancestry component at this value of *K* were chosen as proxies for the true ancestral populations. Specifically, for the European ancestry component we used Italians as the parental group, for the West Siberian component we used the Khanty, for the Central Siberian component we used the Nganasan, for the East Asian component we used the Han Chinese, for the Far Eastern component we used the Koryaks, and for the Yupik-Inuit component we used the Greenland Inuit. We assigned an equal number of individuals per parental group and selected only those individuals with not more than 1% admixture. Because Greenland Inuit, whom we used as a proxy parental population for the admixture signal observed in Chukchi and Naukan Yupik, are themselves heavily admixed, we made an exception for this group and accepted up to 10% admixture in this parental population (as only three individuals met our 1% admixture criteria). Because WT analysis (see below) requires 2*^n^* windows per chromosome (Pugach et al. 2011), we used custom scripts when running PCAdmix to ensure conformity to this requirement while maintaining relatively equal numbers of SNPs per window regardless of chromosome size. For this data set, this was achieved by taking 1024 windows across chromosomes 1–8 (*n* = 10), 512 windows across chromosomes 9–18 and 20 (*n* = 9), and 256 windows across chromosomes 19, 21, and 22 (*n* = 8).

##### AHG and WT Analysis

Once the sequence of admixture events was determined by the AHG approach (see Novel Approaches), we first considered the most recent admixture episode. PCAdmix estimates ancestry along individual chromosomes and assigns chunks of the genome to any of the three or more ancestral populations. In estimating the date for the most recent episode, we treated the population receiving the gene flow as already admixed, and its ancestry composed of ancestry blocks originating from previous admixture events, that is, we treated all blocks of ancestry assigned by PCAdmix to two (or more) older admixture contributors (as determined by AHG) as the same, and considered this part of the genome as coming from population 1. The part of the genome assigned by PCAdmix to the most recent admixture contributor (as determined by the AHG) was considered as originating from population 2. We then proceeded to use WT analysis (Pugach et al. 2011) to estimate the width of ancestry blocks contributed by these two ancestral groups, and estimated the date of admixture based on simulations described previously (Pugach et al. 2011), using simulations with the rate of admixture parameter closest to the populations being analyzed. To estimate the earlier admixture dates the approach was as follows: The blocks of most recent ancestry were masked, that is, excluded from the analysis, and the signal of admixture (the rate of admixture and the size of ancestry blocks) in the remaining part of the genome was analyzed as before. The resulting reduction in genome size was taken into consideration when the result was compared to the simulated data to obtain the date of admixture estimate. Since the first version of the WT admixture dating method (Pugach et al. 2011) is restricted to admixture events involving two parental populations, the adaptation introduced here to this previously published approach was validated in various ways, in particular with simulations of various admixture histories (supplementary text S1*D* and fig. S20, Supplementary Material online) and additional gene flow (supplementary text S1*E* and fig. S21, Supplementary Material online). We also assessed the time-depth resolution of the method (supplementary text S1*F* and fig. S18, Supplementary Material online), and its robustness to misspecification of parental groups (supplementary text S1*G* and fig. S22, Supplementary Material online), as well as addressed concerns related to potential errors in ancestry estimation by PCAdmix and phasing errors (supplementary text S1*H* and figs. S23 and S24, Supplementary Material online). These confirm that the method results in the accurate estimation of dates of multiple admixture events.

### Data Availability

To comply with the informed consent under which the samples were obtained, we make the data available upon request by asking the person requesting the data to agree in writing to the following restrictions: 1) The data will only be used for studies of population history, 2) the data will not be used for medical or disease-related studies, or for studies of natural selection, 3) the data will not be distributed to anyone else, and 4) no attempt will be made to identify any of the sample donors.

## Supplementary Material

Supplementary tables S1–S3, figures S1–S24, and text S1–S3 are available at *Molecular Biology and Evolution online* (http://www.mbe.oxfordjournals.org/).

Supplementary Data
