# Supplementary material for: The Complex Admixture History and Recent Southern Origins of Siberian Populations
Source: Mol Biol Evol. 2016 Mar 18;33(7):1777–95. doi: 10.1093/molbev/msw055 (PMC4915357; doi:10.1093/molbev/msw055)
Supplement: Supplementary Data [file supp_33_7_1777__index.html]

The Complex Admixture History and Recent Southern Origins of Siberian Populations — Supplementary Data 

# The Complex Admixture History and Recent Southern Origins of Siberian Populations

## Supplementary Data

files

- Supplementary Data - pdf file
