# Supplementary material for: The Complex Admixture History and Recent Southern Origins of Siberian Populations
Source: Mol Biol Evol. 2016 Mar 18;33(7):1777–95. doi: 10.1093/molbev/msw055 (PMC4915357; doi:10.1093/molbev/msw055)
Supplement: Supplementary Data [file supp_msw055_Pugach_Siberia_SOM_Revised_updated.pdf]

TEXT S1. SUPPLEMENTARY MATERIALS.......... 1
S1A. 3-population test and TreeMix results.......... 1
S1B. Validation of the Admixture History Graph method.......... 2
S1C. Validation of AHG results for South Siberian populations..........3
S1D. Validation of the modifications to the admixture dating method based on wavelet transform analysis..........5
S1E. Simulations of additional gene flow.......... 7
S1F. Simulations to determine the time-depth resolution of the method..........8
S1G. Simulations to assess the robustness of the method to mis-specification of parental groups..........9
S1H. Addressing other concerns with dates of admixture inference..........10

TEXT S2. SUPPLEMENTARY NOTE ON HISOTRY AND ARCHAEOLOGICAL RECORD OF SOUTH SIBERIA (ALTAIANS, TUVANS, BURYATS)..........13

TEXT S3. SUPPLEMENTARY NOTE ON GENETIC HISTORY AND ARCHAEOLOGICAL RECORD OF INDIVIDUAL POPULATION GROUPS..........16
S3A. Yakuts and Dolgans.......... 16
S3B. Tungusic-speaking populations.......... 19
S3C. Samoyedic-speaking populations.......... 22
S3D. Populations of the Russian Far East: Chukchi and Naukan Yupik..........24
S3E. General comments.......... 27

Supplementary References..........29
Supplementary Tables S1-S3.......... 35
Supplementary Figures S1-S23..........54

TEXT S1. SUPPLEMENTARY MATERIALS

S1A. 3-population test and TreeMix results

The 3-population test (Patterson et al. 2012) is based on allele frequency correlations across populations, and the observation of a significantly negative value of the f3 statistic is taken as clear evidence of admixture. We have applied this test (in its TreeMix implementation) to the Siberian data, and although most of the admixture signals inferFar Eastern and discussed in the manuscript are confirmed (see Table S2 for all significantly negative values of f3), some patent signals of admixture, which are confirmed by multiple other independent analyses and attested to historically are missed. In particular, the test confirms that the South Siberian populations show complex signals of admixture, and also confirms the signals of admixture in the Turkic-speaking Dolgans of northern Siberia. Yet, while historical accounts (supported by the results of other analyses based on genetic data, see Text S3A) suggest that the Dolgans trace their ancestry to Yakuts and Evenks (Stapert 2013, and references therein), Yakut ancestry in the Dolgans is not confirmed by the f3 statistic. Also, whereas the three-population test provides statistically significant evidence that the southern Tungusic-speaking populations (Oroqen, Hezhen, Xibo) trace their ancestry to populations related to the modern day Koryaks, Nganasan and Mongolians (as is suggested by other analyses in Text S3B), no such signal is detected for the northern Tungusic-speaking groups (Even and Evenks); the strong signal of recent gene flow into these groups from the neighbouring Yakuts and Koryaks (particularly evident in the analysis of IBD segments) is not identified either. Similarly, while the test supports a signal of admixture in the Chukchi when Naukan Yupik and Koryaks are used as source populations, the signal of the reciprocal gene flow from the Chukchi into the Naukan Yupik is not supported. In addition, while the Samoyedic-speaking populations (except the Nganasan) appear to be closely related based on PCA, ADMIXTURE, AHG, and the analysis of IBD segments, the three-population test fails to detect Khanty ancestry in the Selkup, while it does identify it in the Nenets groups.

We also attempted to investigate the history of admixture in the Siberian populations with the TreeMix approach (Pickrell & Pritchard 2012). This approach infers a maximum-likelihood tree and then sequentially adds migrations that reduce the residuals between the observed data and the fitted data. However, even after ten migration edges were added (Fig.S7A) the residuals were still quite large (24.9 standard errors; Fig.S7B). The results are difficult to interpret due to the close relationship between the populations being analysed, although the analysis does confirm that all

present-day Siberian populations trace their ancestry to South Siberia. Some other results reported in the Main Text and Text S3 are also supported, e.g. additional gene flow into the Samoyedic-speakers from populations related to the modern day Nganasan and Khanty. Admixture events suggested by PCA, ADMIXTURE, analysis of the IBD segments and multiple regression analyses (and in many cases supported by the f3 statistic or independent genetic analyses and attested to in the historical records) that are nevertheless not inferred by TreeMix include: Finno-Ugric gene flow into Russians, recent gene flow from Yakuts into the neighbouring Even population, and reciprocal gene flow between Chukchi and Naukan. In addition, the Dolgans are not being modelled as admixed between Evenks and Yakuts, and the impact of the Xiongnu invasions (Asian admixture in the northern Turkic- and Tungusic-speakers, see Text S3E below) is also not observed. At the same time, a number of inferred migration edges are hardly plausible, e.g. the gene flow from the San population of southern Africa into the Buryats of south Siberia, or the gene flow from the Han Chinese into the San. Also unlikely is the gene flow edge inferred between the Hezhen (southern branch of the Tungusic language family) and the branch leading to all the northern Tungusic and Far Eastern populations as well as Greenland Inuit (this gene flow therefore could not have been recent, and must have preceded the migration of Paleo-Yupik-Inuit into the Bering Sea region).

While significant f3 scores are taken to indicate admixture, nonsignificant tests do not necessarily indicate its absence, as processes such as genetic drift following admixture could mask the signal (Patterson et al. 2012). This could affect for example admixture signals in the Yakuts - a population which has been shown by the analysis of the distribution of lengths of IBD segments to have recently experienced a founder effect (Text S3A). In addition, given complex admixture histories with multiple admixture events as suggested by the ADMIXTURE analysis (Fig.3), the tests based on pairwise comparisons might not be very informative. Similarly, it is not surprising that the tree inferred by the TreeMix analysis appears to be a poor fit to the data, since all other results suggest recent shared ancestry followed by complex multiple and often reciprocal admixture events; i.e. the Siberian populations did not experience a tree-like population history.

S1B. Validation of the Admixture History Graph method

In order to validate the AHG method, we generated (using R) populations with all the possible combinations of admixture proportions A, B and C such that in the populations reconstructed as they were at the time of admixture the initial ancestry component A was allowed to vary around 10%, 20%, 30%, 40% and 50% (with B=1-A), and the later contribution of C similarly varied from around 10%, 20%, 30% etc to around 90%. For each of the different admixture histories 100,000 outcomes were generated. We then ran the covariance test described in the Materials and Methods

section of the main text to determine the sequence of admixture events. For sample sizes of 10 individuals the sequence of admixture events was inferred correctly for all values of A (100% of outcomes with the correct history inferred), unless the initial proportion of A or C was less than 10%, or the initial proportion of C was over 90%, bringing the final proportion of A to below 5%. When the final proportion of ancestry A in a population dropped to below 5%, the performance of the method diminished slightly (Fig.S2A).

To determine if the test is useful for more complicated admixture scenarios we added another ancestry component (D) to the hypothetical populations already containing ancestries A, B, and C at various mixture proportions. We tested various possible mixture combinations that would allow for the final proportions of any ancestry to be above 5% (Fig.S2B). For sample sizes of 10, the admixture history is inferred correctly more than 95% of the time for a wide range of possible permissible values of A and C (0.2-0.8) and for values of D up to 70%. (Fig.S2B).

Additionally, we explored a scenario where one admixture event between two already admixed populations leads to the appearance of a population with four sources of ancestry ((AB)(CD)) (Fig.S2C). Again, for sample sizes of 10, the admixture history is inferred correctly more than 98% of the time for a wide range of possible permissible values of A and C and for different mixing ratios of AB and CD (Fig.S2C).

S1C. Validation of AHG results for South Siberian populations

Altaians

For the Altaians, the configuration most supported by the AHG calculation is (European, Western Siberian)(Central Siberian, EAsian), shown in Fig.7B. This graph is supported by three out of four sets of covariance tests across the top 30% of the ADMIXTURE runs (see Main Text and Table S3), while the remaining set of tests involving West Siberian, Asian and European ancestries suggest Western Siberian was coupled with Asian, rather than European. Because the admixture history suggested by the AHG results is different for the Altaians and Tuvans, despite their apparent similarity in ancestry composition as inferred by ADMIXTURE, we tested the admixture tree supported by the AHG for the Altaians (European,West Siberian)(Central Siberian, EAsian) against the tree suggested for the Tuvans (((European, Central Siberian)Western Siberian)EAsian). The question here was: if we simulate the ancestral population under the (((AB)C)D) admixture model using as input parameters the means and variances for the A, B, C and D components as observed in the Altaians, how likely are we to recover the incorrect (AB)(CD) graph using the AHG method?

Based on the results of 100,000 simulations we estimate the probability of this error to be around 5%.

Tuvans

Because Tuvans have two out of four ancestries present at low proportion (namely Western Siberian and European), the covariance tests were largely inconclusive (Table S3), and we had to rely only on covariance tests which did not involve both of these ancestries simultaneously as part of the tested trio. Based on these more reliable tests, out of fifteen possible configurations only three were supported. These three configurations are: (((European, Western Siberian)Central Siberian)EAsian), (((European, Central Siberian)Western Siberian)EAsian), and (((Western Siberian, Central Siberian)European)EAsian).

Nevertheless, analysis of ancestry block structure reveals that blocks of European and Central Siberian ancestry are the smallest and therefore are likely to be the oldest in Tuvans. However, there is clear evidence of recent additional Central Siberian gene flow into Tuvans, as in addition to small blocks they exhibit an abundance of wide blocks not seen in the Altaians (Fig.7C). On the other hand, the average width of Western Siberian ancestry blocks is only slightly smaller than that of Asian ancestry blocks, and although this implies that the Asian ancestry is more recent (in agreement with all the AHG results), the difference is too small to distinguish in terms of inferred admixture dates (the date inferred for these two ancestries is ~1,980 ya). Given this additional evidence, we conclude that the configuration best supported for Tuvans is (((European, Central Siberian)Western Siberian)EAsian), and it was this configuration that we tested against the graph inferred for Altaians (European, Western Siberian)(Central Siberian, EAsian). We tried to simulate the admixture history (AB)(CD) using as the input for A, B, C and D the sample means and variances for the European, Central Siberian, Western Siberian and EAsian ancestry components observed in the Tuvans. However, as part of the simulation we generate the parameter space of all possible combinations of A, B, C and D available for the population at the time of admixture under a given admixture model and observed admixture proportions, and the Tuvan set of parameters was outside such a parameter space, i.e. the (AB)(CD) admixture model (at least in its simplest form without additional gene flow) inferred for Altaians is not possible for Tuvans. For example, if we assume that the Central Siberian and EAsian ancestry we observe in Tuvans originated from the same source population (as in Altaians), then we can estimate the initial parameters in the admixing population. For Tuvans, let

$$mc=0.37 \text{ (observed mean frequency of Central Siberian ancestry)}$$

$$md=0.39 \text{ (observed mean frequency of EAsian ancestry)}$$

$$ma=0.11 \text{ (observed mean frequency of European ancestry)}$$

$$mb=0.08 \text{ (observed mean frequency of Western Siberian ancestry)}$$

(Note that the Tuvans also have 3% Far Eastern and 2% Yupik-Inuit ancestry components, which do not enter into the calculations). From these values we can first estimate the initial frequency of the Central Siberian ancestry in the initial admixing population:

$$mz = mc / (1-(ma+mb)).$$

In this example, the initial mean frequency of the component mc was 0.46. As mz is distributed in the interval (0,1) and follows a truncated gamma distribution, the maximal expected variance is $((1-mz)^2)/3$ or 0.097. We can compare this value for variance with the one that would yield the observed variances in mc and md :

$$vc= 0.0005 \text{ (observed variance of Central Siberian ancestry)}$$

$$vd=0.002 \text{ (observed variance of EAsian ancestry)}$$

$$vz = (1-2*mz) /((vd+md^2) / (vc+mc^2) -1) - mz^2$$

In this example, the value of vz we obtain is 0.46 and this value violates the restriction of 0.097 calculated above.

In addition, we can simulate the admixture history inferred for Tuvans ((AB)C)D) only if the A component assumes the mean and variance of the European component as observed in Tuvans (and B is calculated as 1-A), while if we express A through the Central Siberian component, this admixture graph becomes impossible. In fact the Central Siberian component with the mean and variance as observed in Tuvans is not possible under any simple model of 4-way admixture given the other parameters seen in this population. We therefore suggest that this is complementary indirect evidence for more recent additional Central Siberian gene flow into this population.

S1D. Validation of the modifications to the admixture dating method based on wavelet transform analysis.

We constructed different sets of artificially admixed chromosome 1 from the phased genotypes of

unrelated Yoruban (YRI) and Han Chinese (CHB) individuals and individuals of European ancestry (CEU), downloaded from the International HapMap project home page (International HapMap Consortium et al. 2007). First, we simulated admixture between YRI and CEU haploid chromosomes. Admixed genomes were constructed as described previously (Pugach et al. 2011). Briefly, we started by building a recombination map by drawing from an exponential distribution with weight λ, such that an ancestry switch occurred with probability $1 - e^{-\lambda g}$ for each distance of g Morgans. The λ parameter reflects the time of admixture through the expected number of ancestry switches (or breakpoints) that occur given the rate of admixture and recombination (Pugach et al. 2011). In our previous study (Pugach et al. 2011) we showed that for simulated data, the observed number of breakpoints starts to deviate from the expected value beginning around 50-100 generations since the admixture event (depending on the rate of admixture and the effective population size, Ne) (Fig.S18A), which means that the actual time of admixture the simulated number of breakpoints (parameterized by λ) corresponds to is older than λ. In other words and based on the results obtained previously (Pugach et al. 2011), recovering a λ of 80 in the hybrids would indicate power to recover admixture events which have occurred 85 generations ago, if the rate of admixture was 30%; 100 generations ago, if the admixture rate was 40%, and 115 generations ago, if 30% admixture happened in a population with small Ne (which applies to most of the Siberian populations as evident by the genome-wide pattern of LD (Fig.S11)). To generate artificial genomes we started at the beginning of each simulated chromosome and at each of the recombination points from the recombination map we sampled CEU ancestry with probability α and YRI ancestry with probability 1 - α, where the value of α was sampled from a beta distribution with mean 0.30 and standard deviation 0.10. The following values of λ were simulated: 80, 100, 120. We constructed 20 admixed chromosomes for each value of λ. These artificial chromosomes were then used as one of the parental populations for another simulated admixture event, involving CHB. New hybrids were constructed exactly as above, but the values of λ simulated for this more recent admixture event were: 10, 20, 40, and 60. As before, 20 haploid chromosomes were simulated per population. Thus, in total 12 artificial data sets with different admixture histories were simulated (each recent admixture time point is simulated three times, each time using a different admixture background). To recover these 24 admixture dates (two dates of admixture for each of these artificial populations), we first inferred ancestry along the chromosomes using PCAdmix with the same settings as for the empirical data. Wavelet decomposition of the admixture signal as inferred by PCAdmix was performed following the workflow described for the empirical data. The results are reported in Fig.S20 and demonstrate that the admixture dating protocol (Pugach et al. 2011) which we now modified to apply to dating two (or more) pulses of gene flow into the same

population, does recover both dates of admixture quite accurately. The time-depth resolution of the method is discussed in the next section.

As a further part of the validation scheme we inferred the dates of admixture in Russians as revealed by ADMIXTURE (Fig.3) using two different methods: the one described in this study, and the previously published StepPCO method (Pugach et al. 2011). Although both methods use wavelet transform analysis for the time of admixture estimation, the inference of ancestry along the chromosome is based on different approaches in these two methods. The method used here requires phased data (which potentially introduces phasing switch error (Tang et al. 2006; Andrés et al. 2007)), and the PCAdmix algorithm (Brisbin et al. 2012), which uses HMM to assign ancestry for small non-overlapping windows across the genome. The second method uses the StepPCO approach (which does not require the data to be phased), and the windows taken across the genome are larger and overlapping; furthermore, the assignment of ancestry within each window is based on the PC1 coordinates of the SNPs within the window. In both instances we used Italians and Khanty as proxies for the parental populations (chosen based on the results of ADMIXTURE, Fig.3). Based on the results of PCAdmix the admixture event in Russians is estimated to have occurred 870 years ago (CI: 810-1,020), and the StepPCO method yields a similar date of 750 years ago (CI: 600-960). It has been suggested previously that in the course of their history Russians assimilated and admixed with Finno-Ugric tribes of northeastern Europe (Balanovsky et al. 2008; Khrunin et al. 2013), and the time we infer here with both methods (beginning to middle of the 13th century) is consistent with this potential source of the admixture signal (Alekseeva 2002).

<u>*S1E. Simulations of additional gene flow*</u>

To understand the effect on estimates of time of admixture in situations when the same ancestral population contributes to the admixed population multiple times, we first simulated admixture between YRI and CEU haploid chromosomes at 120, 100 and 80 generations ago (as described in the previous section). Then, on each of these artificial backgrounds one additional admixture event involving haploid chromosomes from CHB was introduced at 60 generations ago (as before), thus producing three sets of artificially admixed populations. For each of these three sets we then introduced additional gene flow from CEU haploid chromosomes. The values of λ simulated for this additional admixture event were: 20, 30 and 40. Furthermore, for each of these time points we tested scenarios involving four different rates of admixture, with α being 0.1, 0.2, 0.3 or 0.4. This produced 36 different admixture histories. As before, to recover the dates of admixture (two dates for each of these admixture histories), we first inferred ancestry along the chromosomes using

PCAdmix with the same settings as for the empirical data. Wavelet decomposition of the admixture signal as inferred by PCAdmix was performed following the workflow described for the empirical data. The results are reported in Fig.S21. As expected, additional gene flow lowers the estimated dates of admixture, as it introduces new wider ancestry blocks into the population, thereby altering older narrower block structure and erasing information on past admixture events. Higher rates of additional gene flow amplify this effect, and the underestimates are most severe for scenarios involving the most recent gene flow (λ =20), and the highest admixture rates (α = 0.4). Furthermore, the confidence intervals for the dates inferred for the earlier vs. later event of admixture overlap considerably. This means that in some situations an earlier admixture event might be inferred to be younger than a more recent admixture event.

S1F. Simulations to determine the time-depth resolution of the method

The power to correctly infer small ancestry tracts characteristic of older admixture events depends a) on the density of informative SNPs present in the data, b) on the degree of genetic differentiation of the source populations and c) on the robustness of the method to the source population being mis-specified. Although we have shown previously that in general the wavelet transform method offers a better resolution than any of the existing admixture dating methods it has been compared to (Pugach et al. 2011), the data itself could pose a limitation on how far back in time the dates of admixture could be recovered. To test this we again constructed different sets of artificially admixed genomes following the strategy described in the previous paragraph, but using the same populations (and same markers) that were used as the source groups for our admixture dating routine, i.e. Koryaks (KRY), Italians (ITA), Khanty (KHA), Nganasan (NGA) and Greenland Inuit (ESK), generating hybrid chromosomes to mimic the admixture histories observed for the Siberian dataset. For the first event of admixture we simulated 22 different values of λ ranging from 20 to 200. For each value of λ 20 admixed chromosomes were constructed. These artificial chromosomes were then used as one of the parental populations for another simulated admixture event. New hybrids were constructed exactly as above, but the values of λ simulated for this more recent admixture event ranged from 10 to 100 (but always keeping this λ smaller than the λ used to generate the background event of admixture). As before, 20 haploid chromosomes were simulated per population. In total 118 artificial data sets for each of the admixture histories were simulated. To recover the dates of admixture (two dates of admixture for each of these artificial populations) we followed the workflow described for the empirical data, we first inferred ancestry along the chromosomes using PCAdmix, and then used wavelet decomposition of the admixture signal as

inferred by PCAdmix to recover the dates. The results are reported in Fig.S18 and demonstrate that most simulated admixture events are inferred to have happened not more than 4,000 years ago. Nevertheless, most of the dates we infer for the Siberian dataset are within the bounds of the method's resolution. Furthermore, they are in good agreement with what has been suggested by other studies, e.g. Haak et al (Haak et al. 2015)and Allentoft et al (Allentoft et al. 2015) which show that large scale bi-directional migrations across the Eurasian steppe in the Bronze age produced major changes in the genetic structure of Eurasia, admixing with and replacing autochtonous populations.

S1G. Simulations to assess the robustness of the method to mis-specification of parental groups

To assess the effect of mis-specification of parental groups on the time of admixture estimates, we similarly constructed different sets of artificially admixed chromosomes (chromosome 1 only), but using CEPH panel Affymetrix 500K SNP chip data (López Herráez et al. 2009) from Siberian (Oroqen), Pakistani (Balochi) and European (French) individuals, generating hybrid chromosomes which matched the Siberian dataset in the most important characteristics such as the degree of separation of the parental groups and the density of markers (24,068 Affy SNPs vs 26,420 Illumina SNPs). First, we simulated admixture between French (FRE) and Oroqen (ORO) haploid chromosomes. We used a rate of admixture of 0.3 and simulated 19 different values of λ ranging from 65 to 260. For each value of λ 20 admixed chromosomes were constructed. These artificial chromosomes were then used as one of the parental populations for another simulated admixture event, involving Balochi (BAL). New hybrids were constructed exactly as above, but the values of λ simulated for this more recent admixture event ranged from 55 to 110 (but always keeping this λ smaller than the λ used to generate the background event of admixture). As before, 20 haploid chromosomes were simulated per population. In total 134 artificial data sets with different admixture histories were simulated. Next, we inferred ancestry along the chromosomes with PCAdmix supplying as parental groups either the true source populations (French, Oroqen and Balochi) or the mis-specified proxies a) Italians from Bergamo in lieu of French, b) Yakut and Burusho or c) Han and Yoruba in lieu of Oroqen and Balochi, respectively. The results of this analysis are shown in Fig.S22A-C, while Fig.S22D shows results of the PCA performed on the SNPs included in the analysis. It is evident that when a reasonable proxy is used (Fst=0.002 between French and Bergamo individuals, as estimated by PLINK) the results are essentially the same (Fig.S22A). Furthermore, for admixture times up to 100 generations ago the results remain reliable even when the proxies are a poor choice for a true source population (Fst=0.016 between Oroqen and Yakuts, and Fst=0.013 between Oroqen and Han)(Fig.S22B). Considerable errors in

admixture time estimation (Fig.S22C) are only observed when the proxies used are dramatically either less (experiment b) or more differentiated (experiment c) than the true source populations are (Fig.S22D). In both examples the dates involving Burusho and Yoruba admixture are over-estimated. In the first scenario, where all three proxy populations are not well differentiated for the SNPs used in the analysis, PCAdmix seems to randomly assign segments of chromosomes it does not "recognize" making the inferred ancestry blocks more uniform in length. In the second scenario one of the proxies used was so different from the true source population (Fst=0.136 between Yoruba and Balochi), that only 3% of the chromosomes were assigned to this ancestry (instead of the simulated 30%), making the ancestry blocks very small and leading to a dramatic overestimation of one of the admixture dates, while the second one was underestimated due to artificial extension of the remaining ancestry blocks. Unfortunately, most of the currently available admixture dating methods require the use of pre-defined source populations. Identification of an appropriate source group is always difficult, especially for populations that have experienced admixture a long time ago, and hence had more time to experience genetic drift. Since incorrect identification of source groups could potentially lead to erroneous conclusions, careful attention is required when identifying parental groups. These experiments however show that PCAdmix is fairly robust to the mis-specification of parental groups (as was already shown in (Brisbin et al. 2012) for admixture events involving two ancestral populations) and that the mistake in the choice of proxies would have to be considerable for it to noticeably affect the inferred admixture dates.

S1H. Addressing other concerns with dates of admixture inference

We assessed the possibility of overestimating the dates of admixture due to potential errors in ancestry estimation by PCAdmix. Indeed, if small errors are being introduced during ancestry estimation (with small blocks of “inappropriate” ancestry being inserted within long continuous blocks), such errors would cause the signal of admixture to appear older. We calculated the widths of ancestry blocks, as inferred by PCAdmix, in Anabar Dolgans and scrutinized the genomewide distribution of block widths for each individual and each ancestry. The results are summarized in Fig.S23. We do not observe an excess of short ancestral blocks, and hence find no evidence for erroneous insertions by PCAdmix.

In addition, for every individual and every window, PCAdmix outputs posterior probabilities obtained from the HMM of assignment to a particular ancestry. To further explore if errors in PCAdmix ancestry estimation affect our dates of admixture inference, in another set of experiments we performed admixture dating using only those PCAdmix windows where the ancestry was

inferred with high confidence (at least 0.8 probability in at least 80% of samples). Comparison of dates of admixture inferred using a full set of windows vs. only high confidence windows (Fig.S24A) reveals no tendency to overestimate admixture dates when all the data are used, and again argues against PCAdmix being the source of systematic error in our dates of admixture inference.

Since it is known that haplotype phasing at the level of the entire chromosome is subject to phasing (switch) errors (Tang et al. 2006; Andrés et al. 2007), we wanted to assess to what extent such potential switch errors might affect our results. For two-way admixture we can directly compare results obtained from phased and unphased data (see the discussion in section S1D of similar dates obtained for admixture in Russians using two different methods). For admixture scenarios involving more than two sources of ancestry, the direct comparison is impossible, since the StepPCO approach (which uses unphased data) cannot handle more than two parental populations and PCAdmix requires phased data. Therefore, to test the effect of phasing on admixture dating in a population with more than two sources of ancestry, we re-phased part of the data using a different phasing algorithm, SHAPE-it (Delaneau et al. 2008). SHAPE-it has been shown to have a lower error rate than BEAGLE (Browning & Browning 2007), which was the phasing algorithm employed for this study. The expectation here was that if phasing switch error has a strong influence on the inferred dates of admixture, then a different switch error rate should result in a different date of admixture estimate. Our results show that dating a three-way admixture event using BEAGLE-phased vs. SHAPE-it-phased data yields almost identical dates (Fig.S24B).

In another set of experiments we took the PCAdmix output and "unphased" it by collapsing each pair of windows corresponding to two phased chromosomes of each individual into one, and then running wavelet transform analysis on such "unphased" chromosomes. Although this is not the same as using unphased data (as "unphasing" is done on windows, not on genotypes), we still expect the small ancestry blocks potentially introduced by switch error to disappear and their effect on dates of admixture estimation to be removed. For example, if the ancestry inferred by PCAdmix in six consecutive windows on two phased parental chromosomes is 1 2 1 2 0 1 and 2 1 2 1 0 1 (ancestry from parental populations 0, 1 or 2), then our "collapsed" chromosome will have the following assignment for these windows: 3 3 3 3 0 1 (3 coding a window with ancestry assigned to both parental populations 1 and 2). It is easy to see that such "unphasing" converts four potentially erroneous small ancestry blocks into one longer ancestry block. If we indeed have such erroneously inferred small blocks of ancestry in our data, "unphasing" should result in a more recent admixture

date. This however is not the case, and the dates of admixture inferred using phased and "unphased" data were almost identical (Fig.S24A). In summary, phasing switch error does not appear to have a strong influence on our results.

TEXT S2. SUPPLEMENTARY NOTE ON HISTORY AND ARCHAEOLOGICAL RECORD OF SOUTH SIBERIA (ALTAIANS, TUVANS, BURYATS)

Some of the earliest archaeological monuments in the Altai region that postdate the Last Glacial Maximum belong to the Afanasevo culture, which flourished from around 5,000 years ago (ya) (Naumov 2006). Morphologically, the skeletal remains associated with this culture show affinities with Europeans (Solodovnikov 2003). These affinities are confirmed by analyses of ancient genomes which demonstrate that the Afanasevo culture can be traced to an immigration of peoples from the Yamnaya culture from the Pontic-Caspian steppe. The same peoples have been shown to have also contributed genetically to populations of northern and central Europe (Allentoft et al. 2015). Around 3,500-4,000 ya the Afanasevo culture was succeeded or replaced by the Okunev culture (Savinov & Podolskiy 2006; Sokolova 2007), the bearers of which have been shown to be genetically distinct from the Afanasevo and manifest genetic affinity towards present-day Native Americans (Allentoft et al. 2015). Controversially, some researchers claim that the Okunev culture has been brought to the Altai region by immigrating peoples (Sinor 1990; Savinov & Podolskiy 2006), while others suggest that it pre-dated, possibly co-existed with, and later assimilated the Afanasevo people (Sokolova 2007). The recent finding that the Okunev people could be related to the Upper Paleolithic Mal'ta individuals from Lake Baikal (Allentoft et al. 2015) that was shown to be genetically related to Native Americans (Raghavan, Skoglund, et al. 2014) is more consistent with the second possibility. The date of 3,360 ya inferred here for the admixture between the European and Western Siberian substrates in Altaians (Fig.7B) fits well with the Early Bronze Age eastward expansion of the Yamnaya people and their potential subsequent contact with the Okunev people in the Middle Bronze Age.

Around Lake Baikal, to the east of the Afanasevo and Okunev cultures and contemporary with them, thrived an independent archaeological culture called Serovo-Glazkovo (dated to 6,200-3,000 ya; (Weber & Bettinger 2010)). Analysis of ancient mtDNA lineages from Serovo-Glazkovo sites of the Baikal region show affinities with modern-day South and Central Siberian and East Asian populations, but not with western Eurasians (Mooder et al. 2006). Yet artifacts from Okunev and Serovo-Glazkovo archaeological sites show reciprocal influence of both traditions on each other, and it is not surprising that this cultural contact might have been accompanied by genetic contact as well (Nikolaev 2004), as is suggested by our results.

The archaeological record of Mongolia for the same time period attests to the emergence of a new grave culture, known as the Slab Grave culture. This cultural tradition persisted in Mongolia from 3,100 to 2,300 ya, and is attributed to the ancestors of the Xiongnu people (also known as Asian Huns), who around 2,000 ya created a powerful empire which for three centuries dominated central and eastern Asia and extended from eastern Kazakhstan to western Manchuria (Rudenko 1962; Taskin 1968; Konovalov 1976; Miniaev 2001). There are notable similarities between the Bronze Age cultures of the Altai region (Okunev culture and its successors) and the Slab Grave culture of Mongolia, thus suggesting extensive cross-regional contacts between the Altai, Mongolia and Lake Baikal (Mooder et al. 2006; Tumen 2008 and references therein). Nuclear and mitochondrial DNA extracted from the skeletal remains contained in a 2,000 year old Xiongnu necropolis in northern Mongolia (Keyser-Tracqui et al. 2003) show affinities to modern-day South Siberian populations and Mongolians (Crubézy et al. 2010; González-Ruiz et al. 2012). And although the dates of around 2,000 ya estimated for the appearance of the EAsian signal in Buryats and Tuvans perfectly correspond to the time period of the Xiongnu empire, we suggest, based on the distribution of the ancestral Asian block widths in populations of South Siberia (Fig.7C), that Buryats must have received additional gene flow from some East Eurasian source. This additional gene flow is likely to have occurred 800-600 ya, in the times of the Mongol empire under Genghis Khan and his successors, which stretched far beyond the modern borders of Mongolia. Hence, we suggest that the dates of EAsian admixture estimated for Buryats are composite and reflect: an older admixture event of around 2,700 ya, as observed in Altaians and as consistent with the dates for the appearance of the Slab Grave Culture in Mongolia associated with the ancestors of the Xiongnu: and a more recent admixture of around 800-600 ya, consistent with the Mongol expansion under Genghis Khan. Furthermore, mtDNA analysis of skeletal remains from Bronze Age archaeological sites in the eastern and western Altai shows that 5,000-3,000 ya mtDNA lineages in the western (Russian) Altai were exclusively of western Eurasian origin, while the mtDNA lineages in the eastern (Mongolian) Altai were exclusively eastern Eurasian. In contrast, all analysed skeletal remains from the Iron Age (2,800-1,800 ya) sites from the eastern and western Altai as well as from Kazakhstan (Pazyryk culture, traditionally associated with the Eastern Scythians and also with the Tarim mummies of western China (Li et al. 2010)), reveal the presence of both western and eastern Eurasian mtDNA haplogroups (González-Ruiz et al. 2012), in agreement with the AHG inferred for Altaians. These findings are also reflected in studies of uniparental markers in modern-day South Siberian populations that show a larger proportion of western Eurasian lineages in the populations of the Altai-Sayan than in the Baikal region (Derenko et al. 2003; Derenko et al. 2006). Since the

peoples with western and eastern Eurasian ancestries co-existed in the Altai for a prolonged period of time (González-Ruiz et al. 2012), it is plausible that the admixture date of ~2,300 ya estimated for the Altaians captures admixture between these local groups and is not associated with any additional migration from the outside.

TEXT S3. SUPPLEMENTARY NOTE ON GENETIC HISTORY AND ARCHAEOLOGICAL RECORD OF INDIVIDUAL POPULATION GROUPS

In the following sections we analyze signals of admixture and discuss the genetic prehistory of populations grouped either by their present-day geographical location or by their linguistic affiliation.

S3A. Yakuts and Dolgans

Although they live in central and northern Siberia, the Yakuts and Dolgans speak Turkic languages related to those spoken in the south. The Yakuts practice animal husbandry like the South Siberian Turkic-speakers, whereas the Dolgans are nomadic reindeer breeders and hunters like their neighbours, the Tungusic-speaking Evenks (Popov 1956a; Tokarev & Gurvich 1956; Vasilevich 1956). Notwithstanding the great geographic distances that separate the Yakuts and Dolgans from their linguistic relatives, their genetic relationship with the South Siberian populations emerges clearly in both the PC and ADMIXTURE analyses. In the PC analysis, the Turkic-speaking populations are not separated along PC1-PC2, and a distinction between the southern (Altaians, Tuvans) and northern (Yakuts, Dolgans) Turkic-speaking groups is first observable only at PC3 and PC4 (Fig.S3). It has been shown before that for spatially structured data the first two PCs accurately capture geography, and genetic information can thus be used to infer geographic origin (Lao et al. 2008; Novembre et al. 2008). Such a genetic trace of pre-migration origins can be discerned for the Yakuts: their position in the PC analysis would place their geographic origin to the east of Tuva and north of Buryatia; this is in good accordance with their purported origins around Lake Baikal (Tokarev & Gurvich 1956; Crubézy et al. 2010).

The hypothesis of a southern origin is also indirectly supported by the results of the IBD analysis. The amount of the IBD segments shared within the Yakuts is relatively high in comparison to other populations (Fig.S10); for instance, they share twice as many segments within the population as the Buryats, even though the census sizes of Buryats and Yakuts are comparable (Buryats – 461,389 individuals, Yakuts – 478,085 individuals; Russian 2010 census). Furthermore, the slow decay in the frequency of small IBD segments (2-10 cM) in comparison to longer segments in the Yakuts (Fig.S10) also suggests a strong founder event (Palamara et al. 2012). Although the Yakuts currently constitute one of the most numerous indigenous ethnic groups in Siberia, this pattern of sharing of

IBD segments is consistent with a recent expansion from a small founding population, which is also supported by uniparental data (Pakendorf et al. 2002; Pakendorf et al. 2006; Zlojutro et al. 2009) and by the relatively high genome-wide LD in the Yakuts (Fig.S11B).

In terms of sharing with other populations, the Yakuts share more and the longest IBD blocks with the Dolgans, who are thought to be partially descended from Yakuts (Stapert 2013, and references therein), but also with the neighbouring Even groups (Sakkyryyr, Sebjan, Tompo), Buryats, and Evenks (Fig.5 and Fig.S12). The sharing of IBD blocks with Buryats is consistent with the idea that the Yakut ancestors once lived in close proximity to the ancestors of modern-day Buryats. The Dolgans share a large amount of long IBD segments with the Yakuts, Evenks, Nganasan and Evens (Fig.S12). While the Yakuts and Evenks are thought to have contributed genetically to the modern-day Dolgans, the affinity towards the Nganasan and Evens is probably indirect, and is best explained by these populations having themselves shared ancestry with the Evenks, although direct admixture with Nganasan cannot be excluded.

The ADMIXTURE results (Fig.3) suggest three sources of ancestry for the Yakuts and the Dolgans: Central Siberian (blue) (59% in the Yakuts, 67% in the Dolgans), EAsian (pink) (30% in the Yakuts, 18% in the Dolgans), and European (light green) (10% in the Yakuts, 11% in the Dolgans). In contrast to their South Siberian Turkic-speaking relatives, the Yakuts and Dolgans lack the Western Siberian (yellow) component.

Analysis of the distribution of the widths of blocks of different ancestries reveals that the blocks of Central Siberian ancestry in Yakuts and Dolgans become progressively wider to the north and west (Fig.S14B). This would be consistent with additional recent gene flow from a population of mainly Central Siberian ancestry. In fact, the widest blocks and the highest variance around the mean are observed in the Taimyr Dolgans, the population with close genetic ties to the Evenks, who in turn have a high proportion of the Central Siberian ancestral component. The inferred dates for the Central Siberian admixture given in Fig.S14A therefore provide a lower bound for this event in the Yakuts. In the Dolgans, the Central Siberian date is likely an underestimate since the resolution for dating this admixture in the Dolgans is relatively low (Fig.S18H). The source of recent gene flow of Central Siberian ancestry to the Yakuts does not appear to be any of the neighboring Tungusic populations, since Yakuts completely lack the Far Eastern component (red) found in all the Tungusic groups (see below); however, given the frequency of this component in the neighboring Evenks and Evens, low levels of gene flow (1-3%) cannot be excluded. In contrast, the Far Eastern

component is observed in both Dolgan subgroups, albeit at very low frequency (less than 3%). This again is consistent with historical accounts of Dolgans tracing some of their ancestry (at least 33%, based on the amount of Far Eastern ancestry) to Evenks (Stapert 2013, and references therein). The absence vs. presence of this Far Eastern component thus genetically differentiates the Yakuts from the Dolgans. The substantially younger signal of European admixture in the Dolgans than the Yakuts can be explained by gene flow from Russians, known on the Taimyr from the 17th century (Stapert 2013, and references therein). Indeed, analysis of the means and variance of the inferred admixture blocks in the Yakuts and Dolgans (Fig.S14B) shows that the blocks of European ancestry are longer in the Dolgans than in the Yakuts and have higher variance, thus providing corroborating evidence for additional European gene flow in Dolgans. This implies that the date of 1,470 years BP inferred for the European admixture in the Dolgans reflects both an older signal of admixture already present in the Yakuts and South Siberian populations, and more recent contact with the Russian population of the Taimyr.

In summary, our results are in good accordance with the hypothesis that the Yakuts originated in South Siberia and stem from a shared ancestral population with Buryats (Tokarev & Gurvich 1956; Crubézy et al. 2010). The two seemingly contradictory pieces of evidence - the absence of a Western Siberian signal in the Yakuts and the different AHGs (which are consistent across the top 30% of the ADMIXTURE runs) inferred for the Yakuts (Fig.S14A and Table S3) vs Buryats (Fig.7B) -- can be explained by drift and differential gene flow: on the one hand, low levels of Western Siberian ancestry could have been lost by drift in the small founding population of the Yakuts (indeed, Western Siberian ancestry is present at only 2% in the Buryats). On the other hand, recent differential gene flow after the divergence of the Yakuts (Central Siberian into the Yakuts and EAsian into the Buryats), as supported by the comparatively wide blocks of Central Siberian and EAsian ancestry in these populations, would have changed the configuration of their AHGs (see Fig.S21 and the simulations of the effect of additional gene flow from a population that was involved in an early admixture event described in Text S1E). The ethnogenesis of the Dolgans, who are linguistically very closely related to the Yakuts, but culturally very close to Evenks, has occurred in recent times and is known to have been influenced by the neighboring Yakuts, Evenks and Russians. Indeed, all our analyses confirm that the Dolgans are most closely related to the Yakuts and the Evenks (although some additional gene flow from Nganasan cannot be excluded with the genetic data), with evidence for recent additional gene flow from a European source.

S3B. Tungusic-speaking populations

Speakers of Tungusic languages are spread from the Yenisey river in the west to the Kamchatka Peninsula in the east, and from the Taimyr Peninsula in the north to China in the south (Fig.3). The Evenks and Evens, whose languages belong to the Northern Tungusic branch, are traditionally highly mobile hunters and reindeer herders who are dispersed over vast territories of Siberia (Levin 1956; Vasilevich 1956), while the linguistically closely related Oroqen from northeastern China are traditionally hunters and horse herders. In contrast, the other Tungusic populations of the Amur region and northern China, such as the Hezhen and Xibo, speak languages belonging to the Southern Tungusic and Manchu branches. The Hezhen are traditionally fishers and hunters (Ivanov et al. 1956), while the Xibo are agriculturalists (Cheboksarov et al. 1965).

In the PCA, the Even and Evenk subgroups sampled all across Siberia show remarkably little genetic differentiation; the Evenk and Even populations are differentiated only along PC3 and PC4 (Fig.2, Fig.S3) and along PC2 in the analysis comprising only the Siberian populations (Fig.S4). Surprisingly, there is no observable structure within either of these populations, even though the samples are from locations that are up to 2700 km apart.

Analysis of the IBD blocks suggests that the Evenks and Evens have a lower effective population size than the Tungusic peoples of the south (e.g. the Oroqen), as the former share more IBD segments within the population than the latter (Fig.S10). This is also evident in the pattern of genome-wide LD, where the Oroqen have lower genome-wide LD values than the Evens or the Evenks (Fig.S11A). Overall, Evenks and Evens share a large number of long IBD segments, and they also share such segments with neighbouring populations: the Evenks with the Nganasan, Dolgans, Yukaghirs and Nenets, the Evens with the Yukaghirs, Dolgans and the Yakuts (Fig.S12).

The results of the ADMIXTURE analysis (Fig.3) reveal that the Tungusic populations from all across Siberia, with the exception of the Xibo, trace their ancestry to three sources (Central Siberian (blue), EAsian (pink) and Far Eastern (red)). The Central Siberian component is higher in the west than in the east and south, and ranges from 85% in the Evenk subgroups and 64% in the Evens of Kamchatka to 40% in the Oroqen, 24% in the Hezhen, and 15% in the Xibo. The Far Eastern component is highest in the easternmost Even subgroups and lowest in Tungusic populations of western and southern Siberia; this ranges in frequency from 28% in the Evens of Kamchatka to 6% in the Evenks, 5% in the Oroqen, 4% in the Hezhen, and 2% in the Xibo. The EAsian component is seen at highest frequency in the southeast, where it reaches 54% in the Oroqen and 71% and 80% in

the Hezhen and the Xibo, respectively, while it is present at around 10% in the Evenks and Evens. The Tungusic peoples are further characterized by the complete absence of any European (light green) ancestry (except for the Xibo, who have 2% of this ancestry component).

Analysis of the ancestry block width inferred by PCAdmix (Fig.S15B) indicates that the blocks of Central Siberian ancestry are wider and the variance around the mean is higher in the Evenk groups, while the blocks become narrower and the variance decreases in the Oroqen, Hezhen, and Xibo. This pattern is compatible with a scenario of additional gene flow from a population of mainly Central Siberian ancestry into the western Tungusic groups (Evenks, and Evens from Sakkyryyr, Sebjan and Tompo). In addition, it has been shown previously with mtDNA and Y chromosome data that the Sakkyryyr and Sebjan Even subgroups have experienced substantial amounts of admixture from Yakuts (Pakendorf et al. 2007; Duggan et al. 2013). Our results based on autosomal data confirm this conclusion, as the Sakkyryyr Evens are much closer to the Yakuts than to the other Tungusic groups in all measures of genetic relatedness based on IBDs, while the Sebjan and Tompo Evens appear substantially closer to the Yakuts than do the Berezovka and Kamchatka Evens (Fig.S12). Also, the ADMIXTURE results show that Sakkyryyr Evens carry around 1.5% European ancestry (presumably contributed by the Yakuts). Assuming, that the elevated levels of EAsian ancestry in the Sakkyryyr, Sebjan and Tompo Evens in comparison to the Kamchatka and Berezovka Evens were contributed via admixture from the Yakuts, we can roughly estimate that they have received 14-40% gene flow from Yakuts (also see Fig.S5 for the ADMIXTURE results at K=7). On the other hand, the widest blocks of EAsian ancestry, and the highest variance around the mean, are observed in the Oroqen, Hezhen, and Xibo (Fig.S15B). This suggests substantial recent gene flow from a EAsian source population into the southern Tungusic peoples. Similarly, the distribution of the Far Eastern ancestry blocks is consistent with some additional contact between the Even subgroups closest to Kamchatka and the Koryaks, although this gene flow was not as abundant as the Central Siberian admixture detected in the Evenks and western Even subgroups or the EAsian admixture into the Tungusic populations of the south (Fig.S15B).

The AHGs (consistent across the top 30% of the ADMIXTURE runs) therefore yield a composite picture of the successive layers of differential admixture experienced by the Tungusic subgroups, leading to different configurations: ((Far Eastern, EAsian) Central Siberian) for the Evenks and western Even subgroups, and ((Central Siberian, Far Eastern) EAsian) for eastern Evens, Oroqen and Hezhen (Fig.S15A). Since the two eastern Even subgroups (Berezovka and Kamchatka), as

well as the Oroqen and Hezhen have experienced the least amount of recent gene flow from a population of Central Siberian ancestry, we assume that their AHG reflects the ancestral admixture history of all of the Tungusic-speaking populations. Later substantial gene flow from a source or sources of predominantly Central Siberian ancestry in the Evenks and western Even subgroups (Sakkyryyr, Sebjan, and Tompo) will have considerably lowered the estimated age of Central Siberian admixture for these groups, leading to this being reconstructed as the most recent event (see Fig.S21 and the simulations of the effects of additional gene flow from a population that was involved in previous admixture described in Text S1E). We therefore did not date the admixture events for the Evenks and western Even subgroups, but only show the inferred AHG (Fig.S15A). Substantial gene flow from a population of predominantly EAsian ancestry will have lowered the estimated age of the EAsian admixture in the Tungusic populations of the Amur region, but this would not have altered the AHG configuration.

The earliest ages estimated for the first admixture event are observed in the Oroqen (3,120 ya; CI 2,700 – 3,630) and Hezhen (3,120 ya; CI 2,910- 3,930); however, these are unlikely to reflect the true age of the ancestral admixture event, as these dates exceed the resolution limit available for the Tungusic-speaking populations of ca. 2,700 years (Fig.S18F). These dates probably reflect the difficulty of PCAdmix to correctly assign fragments of chromosomes to either Central Siberian or Far Eastern ancestry when these fragments are small; alternatively this Central Siberian-Far Eastern substrate might represent the ancient diversity shared by all the Tungusic-speaking people, with additional Central Siberian admixture experienced only by Evens and Evenks. The oldest dates for the EAsian admixture of 1,860 ya (CI: 1710-2160) are seen in the Berezovka and Kamchatka Evens, who have experienced the least amount of recent EAsian admixture. However, since this date is at the very limit of our resolution (Fig. S18F) this gene flow could have happened earlier. This EAsian admixture event is likely to have occurred in the south, since all the Tungusic populations carry the EAsian ancestry component, and might have been associated with the Xiongnu empire (see Text S2), as although the Xiongnu empire was centred around modern-day Mongolia, it stretched far eastward to the Amur River tributaries and Western Manchuria (Barfield 2001).

Previous studies have suggested either Lake Baikal (Vasilevič 1969) or the Amur River (reviewed in (Tugolukov 1980)) as the place of origin of the Tungusic populations. Based on our results, we can reject the area around Lake Baikal, as all populations in our study from this area have European

ancestry tracing back more than 3,200 years ago, and the Tungusic-speaking populations lack this substrate. We therefore suggest the Amur River as the place of origin of the Tungusic populations, which is also in good agreement with the archaeological evidence. Archaeological sites along the Amur are numerous, and the earliest are dated to at least 13,000 ya (Popov & Tabarev 2008). The Far Eastern and Central Siberian admixture inferred by our analysis could correspond to the latest Neolithic material culture of the region, which is dated to 4,000-3,000 ya and is associated with the appearance of fundamentally new stone-working techniques, which are ascribed to the arrival of new peoples (Derevianko & Clark 1965).

S3C. Samoyedic populations

The Samoyedic languages spoken by traditionally semi-nomadic reindeer-herding as well as hunting populations of western Siberia and the Yamal and Taimyr Peninsulas of the northernmost Russian Arctic belong to the Uralic language family, which also includes Finno-Ugric languages such as Finnish and Hungarian in Europe and Khanty in western Siberia. From historical data it is known that some Samoyedic languages were spoken in the Altai region of south Siberia, but these are now extinct (Vajda 2009). As shown by the PC analysis (Fig.2), the Nganasan are separated from their linguistic relatives, showing affinities with Evenks and Evens instead. The separation of the Nganasan from the other Samoyedic speakers is further underlined by the ADMIXTURE analysis and the analysis of the genome-wide LD (Fig.3 and Fig.S11C).

The distinguishing characteristic of the Samoyedic-speaking populations other than the Nganasan is that they trace their ancestry to at least three different sources. Both the Selkup and the Nenets have substantial proportions of Western Siberian (yellow) (Selkup: 52%, Yamal Nenets: 48%, Taimyr Nenets: 44%) and Central Siberian (blue) (Selkup: 26%, Yamal Nenets: 44%, Taimyr Nenets: 50%) ancestry. They also have European ancestry (light green) (Selkup: 13%, Yamal Nenets: 4%, Taimyr Nenets: 2%). In the Nganasan we observe neither a Western Siberian nor a European component. In fact, already at K=4 individuals in this population are ascribed their own component (Central Siberian; cf. Fig.S5), indicating a different history of this population compared to the other Samoyedic peoples.

AHG analysis (Table S3) indicates that in the Selkup and Nenets the Western Siberian gene flow is the latest contribution (Fig.S16A; this is consistent across the top 30% of the ADMIXTURE runs).

Furthermore, wider on average blocks of Western Siberian and Central Siberian ancestry, and higher variance around the mean, suggest that all populations have experienced multiple events of gene flow from these sources (Fig.S16B). Therefore, the dates of admixture reported below are composite dates, and reflect an older date of admixture as well as this more recent additional gene flow. We estimate the date of admixture between the European (light green) and Central Siberian (blue) ancestries to be 2,700 ya (CI 2,490 -3,120) in the Selkup, and 2,490 ya (CI 2,310 – 3,120) in the Yamal Nenets (Fig.S16A). We did not attempt to date this signal of admixture in the Taimyr Nenets subgroup, because the estimated proportion of admixture in this group is too low for our method to work reliably (see Fig.S2A and Text S1B). The date obtained for the more recent admixture with the Western Siberian ancestral group is 1,590 ya for the Selkup and the Yamal Nenets (CIs 1,470-1,710 and 1,470-1,860, respectively); the estimated date for the Taimyr Nenets is more recent at 1,170 ya (CI 1,020 – 1,260). In this group we also observe wider and more variable ancestry blocks (Fig.S16B), which is consistent with the gene flow either being more recent, or occurring over an extended period of time. Importantly, the inferred dates for the Western Siberian admixture in the Samoyedic-speakers are at the limit of the resolution (Fig.S18E) and could therefore be an underestimate of the true date.

The sequence of admixture events and the dates of admixture estimated for the Selkup and Nenets (Fig.S16A) are relatively close to those obtained for Tuvans (see Fig.7B). Although the dates for the prehistoric mixture between the European and Central Siberian components are more recent for the Samoyedic populations (~2500-2700 ya) than the dates estimated for Tuvans (~3100 ya), this is most likely due to later additional gene flow into the Selkup and Nenets from some population of predominantly Central Siberian ancestry, as discussed in the main text, and also due to probable additional recent admixture with Russians (Fig.S16B). On the other hand, the EAsian component we observe in Tuvans (and Altaians) is not seen in any of the Samoyedic groups, and its arrival in the Sayan plateau (and the Altai region) predates the Western Siberian admixture estimated for the Samoyeds. Our analyses therefore suggest the following history for the Selkup and Nenets: it is likely that modern-day Selkup, Nenets, and Tuvans trace their ancestry to the same source population, and either modern-day Tuvans migrated to the Sayan plateau after their split from the ancestors of modern-day Selkup and Nenets, or the Sayan plateau itself was the ancestral homeland of these populations. Before later migrations brought Asian genes into this area of South Siberia, the ancestors of the Samoyeds started to expand north (e.g. it is thought that the northward migration of the Kulai people of the southeastern part of Western Siberia, which some suggest were the ancestors

of the modern-day Samoyedic populations, took place approximately 1500 ya (Pletneva 1987)). Later admixture with some population of primarily Western Siberian ancestry would have added further Western Siberian ancestry to the gene pool of the Samoyedic groups, resulting in a younger date for the Western Siberian gene flow than that estimated for the Tuvans. The date for this mixture in the Selkup and Nenets of 1,590 ya coincides with what is known as the Great Migration Period, which took place in Eurasia 1,700-1,200 years ago. This was characterized by massive movements of people across the continent and could have been precipitated by climate deterioration (Larsen et al. 2008) or the rise of the Hunnic Empire under Attila the Hun and the invasion of Europe by the Huns of Central Asia (Kim 2013). Its impact on the genetic landscape of Europe and Asia was profound (Kim 2013). However, since the inferred date of the Western Siberian admixture is at the limit of resolution available for the Samoyedic-speakers (Fig.S18E) we cannot rule out that this admixture is actually older. The Nenets subsequently experienced additional gene flow from some population of Central Siberian ancestry (such as the Nganasan), which probably happened not before 1000 ya, after the Nenets settled on the Yamal and Taimyr (Vasiliev 1974). Blocks of European ancestry that are on average wider in Nenets than anywhere else in Siberia, and a larger variance around the mean, also suggest additional later gene flow, probably from Russians.

All our analyses indicate that the Nganasan, the northernmost indigenous people of Siberia, are quite distinct from the other Samoyedic populations. They lack the Western Siberian and the European ancestry components, which are both characteristic of the other Samoyedic groups. Genetically, they appear to be much closer to the neighboring Tungusic speakers and the Yukaghirs than to the Selkup and even the neighboring Nenets. This leads us to conclude that the Nganasan have a different genetic history than the other Samoyedic groups and that they were linguistically (Popov 1956b; Dolgih 1957; Helimskij 2000), but not genetically, assimilated by the Samoyedic populations who around 1,000 years ago migrated from the south to the northern Arctic.

S3D. Populations of the Russian Far East: Koryaks, Chukchi and Naukan Yupik

The Chukotka and Kamchatka Peninsulas were once part of ancient Beringia, a vast late Pleistocene glacial refugium for modern humans and a center of their dispersal into the New World (Hoffecker et al. 2014). The oldest reliably dated human remains from Beringia come from the Kamchatka Peninsula and are 13,000 years old (Goebel et al. 2010). Today, native populations of western Beringia – in particular, Chukchi, Koryaks and Naukan Yupik – speak distinct languages and differ

in their social organization. The Chukchi and Koryaks, who speak languages of the Chukotka-Kamchatkan family, used to lead a nomadic lifestyle and practice inland reindeer herding in combination with coastal hunting and fishing as a mode of subsistence. The Naukan Yupik, who live in close proximity to the Chukchi, speak a Yupik language (which belongs to the Aleut-Yupik-Inuit language family extending across Alaska, the Canadian Arctic and Greenland) and traditionally engaged solely in coastal sea mammal hunting (Antropova & Kuznetsova 1956; Menovschikov 1956).

The PCA results reveal genetic affinities between the Koryaks, the Chukchi and the Naukan Yupik. The genetic relationship between the Chukchi and Koryaks is further confirmed by the ADMIXTURE analysis, which in addition suggests reciprocal gene flow between the Chukchi and Naukan Yupik, albeit with gene flow predominantly from the Naukan Yupik into the Chukchi.

Strikingly, both the short-range and long-range genomewide LD is substantially higher in the Naukan Yupik than in the Chukchi, who often reside in neighboring villages or even the same settlements. This would imply that in contrast to the Chukchi, the Naukan Yupik underwent an additional strong bottleneck. Overall, the populations of Chukotka and Kamchatka extensively share IBD blocks among themselves as well as with the Yukaghirs and the neighbouring Even subgroups (Fig.5, Fig.S12). The Koryaks are observed to share fewer IBD blocks with the Naukan Yupik than the Chukchi do. This is probably due to admixture between the Naukan and the Chukchi, as also indicated by the ADMIXTURE analysis.

Fitting a multiple regression of genetic distances on geographic distances reveals that the Naukan Yupik exhibit elevated genetic distances with all other populations (except for the neighbouring Chukotko-Kamchatkan populations and Greenland Inuit) (Fig.S13). This pattern of generally elevated genetic distances, along with the genetic proximity to Greenland Inuit as suggested by the SpaceMix analysis (Fig.S8), is consistent with the viewpoint that the ancestors of the Naukan Yupik back-migrated to Siberia from the Americas (Tamm et al. 2007; Reich et al. 2012a; Achilli et al. 2013).

To estimate dates of admixture for the gene flow between the Naukan Yupik and Chukchi, the Koryaks and Greenland Inuit were taken as proxies for the parental populations. We estimated 7% of the Far Eastern component in the Naukan Yupik, and 40% Yupik-Inuit ancestry in Chukchi. The

estimated time of admixture for the Naukan Yupik is 1,590 ya (CI 1,470 – 1,710), and for the Chukchi 1,470 ya (CI 1,380 – 1,710), suggesting a single event of reciprocal admixture. The date of this admixture event is in good accordance with the time of the first appearance of the Neo-Yupik-Inuit culture in Siberia (Rainey & Ralph 1959) as well as with the first appearance of cultural complexes in Alaska similar to the ones on Chukotka (Dixon 2013).

The presence of tribes of hunters and fishers on the Kamchatka and Chukotka Peninsulas is documented archaeologically from around 13,000 ya (Vasil'ev et al. 2002; Goebel et al. 2010), yet cultural traits that can be attributed to the putative ancestors of Chukchi and Koryaks do not appear in the archaeological record until around 3,000 ya (Dikov & Clark 1965). The origin of the Naukan Yupik is still widely debated. While Paleo-Yupik-Inuit populations migrated into the Bering Sea region from Siberia around 5,500 ya (Rasmussen et al. 2010), the Naukan Yupik are the descendants of the pre-Inuit Neo-Yupik-Inuit populations which have been recently shown to have a migration history independent of Paleo-Yupik-Inuit (Raghavan, DeGiorgio, et al. 2014).While some authors suggest that the Naukan Yupik are a group that never crossed the Bering Strait, but who persisted in Siberia (e.g. (Dikov & Clark 1965)), others think that their presence in Chukotka is the result of a back-migration from North America (Tamm et al. 2007; Reich et al. 2012b; Achilli et al. 2013; Raghavan, DeGiorgio, et al. 2014). Based on the typology of elements of the harpoon complex, the distinctive features of the Yupik-Inuit culture are not seen on Chukotka until around 2,200 ya and the earliest Yupik-Inuit sites along the coasts are dated to around 2,000 ya (Dikov 1977). Radiocarbon dates, however, put the earliest Yupik-Inuit cultures on Chukotka (Old Bering and Okvik cultures) at ca. 1700-1500 ya (Rainey & Ralph 1959). The estimated date of admixture also coincides with severe climatic changes in Greenland (McGhee 2001), which could have incited large-scale migrations and relocation of peoples. It is also in agreement with the age of 1,000-2,000 years estimated for the expansion from Alaska and westward spread of the mtDNA haplogroup A2a, which is typical of the Yupik-Inuit populations of North America and Siberia (Achilli et al. 2013).

In order to infer the probable homeland of the Naukan Yupik in the Americas, we recalculated the regression coefficients (see Main Text), assigning to the Naukan Yupik the geographic coordinates of a range of locations in Alaska, Canada and Greenland that are home to the current Yupik and Inuit populations. The largest improvement was achieved when the location of the Naukan Yupik was assigned to the geographic area circumscribed by the south-west of the Yukon Territories, the western part of the Northern Territories and British Columbia (see Fig.S17) (Rsq = 0.613, $p<0.001$).

This area is outside of the region where Yupik and Inuit settlements are attested; however, genetic admixture with the Chukchi will have reduced the genetic distance between the Naukan Yupik and Chukotka and thus will also reduce the geographic distance needed to provide the best match between genetics and geography. Given that from the perspective of geographic distances the territory of origin we reconstruct lies to the east of Alaska, we can conclude that the origin of the Naukan Yupik was neither in Chukotka nor in Alaska, but somewhere further east. This conclusion should be taken with caution however, since we did not consider how strongly this result could be affected by genetic drift and admixture, both processes shown here to have played a role in the demographic history of the Naukan Yupik.

Our results support an origin of the Naukan Yupik in North America and their subsequent back-migration to Siberia. Furthermore, the dates of reciprocal admixture estimated for the Naukan Yupik and the Chukchi of ca. 1,500 ya are in good agreement with the time of the first appearance of the Neo-Yupik-Inuit culture in Siberia. Yet given that the inferred date of admixture is close to the limit of the resolution available for the Chukchi and the Naukan Yupik (Fig.S18I) we cannot rule out the possibility that the date of admixture is actually older.

S3E. General comments

For many of the Siberian populations, we can identify and distinguish signals of admixture events shared between populations or population subgroups (e.g. the signal of European admixture shared by all the Turkic-speaking groups) from local differential gene flow (e.g. the recent Yakut admixture which affected some, but not all, Even subgroups). We observe what might be the genetic impact of the powerful Xiongnu empire of ca. 2,000 years ago on the surrounding populations. The Xiongnu empire was centred around today's Mongolia, but stretched far beyond its current borders. The early dates of EAsian admixture that we infer for the populations of the Altai and Sayan Mountains (Altaians and Tuvans) as well as the populations which trace their origin to lake Baikal (Buryats, Yakuts, and ultimately Dolgans) might well be the genetic legacy of this powerful empire, rather than reflecting the impact of the much more recent Mongol empire under Gengis Khan and his successors. Similar early EAsian admixture that might reflect Xiongnu influence is also observed in all the Tungusic-speaking populations; the fact that this ancestry is shared by all the Tungusic peoples suggests that the gene flow must have happened before Evens and Evenks (and Oroqen) diverged and expanded across Siberia. In contrast, the Mongol empire founded by Gengis Khan,

which lasted from 1206 to 1368, and was the largest empire in the world, stretching from central Europe to the Sea of Japan and from southern Siberia to India and Indochina, appears to have had a genetic impact only on the Buryats - a population in immediate proximity to Mongolia. This is consistent with the idea that the Mongol empire was maintained via appointed local viceroys with little if any physical presence of the conquerors. In fact, Russian territories were in subjection to the Golden Horde (part of the Mongol empire and its successors) for nearly 300 years, yet no evidence of this was detected in Y chromosome analyses of Russians (Balanovsky et al. 2008), and the absence of this signal is confirmed here with dense autosomal data (see Text S1D for the analyses and discussion of the admixture in Russians). Interestingly, it was suggested that the expansion of the Yakuts from South Siberia (Tokarev & Gurvich 1956; Crubézy et al. 2010) was precipitated by the advancing armies of Genghis Khan (Pakendorf et al. 2006). Our results are consistent with this hypothesis: unlike the Turkic-speaking populations of southern Siberia (as well as the Mongolic-speaking Buryats), the Turkic speakers of northern Siberia show a signal of earlier EAsian gene flow (possibly associated with the Xiongnu), but not of the later EAsian gene flow that could be associated with the Mongol expansion under Genghis Khan. The same pattern is observed in all the Evenk and Even subgroups. Since some researchers suggest that the separation of the Evenks and Evens from the South Tungusic-speakers (e.g. Hezhen) occurred only ca. 800 years ago (Tugolukov 1980), it is possible that the invading Mongolian armies also forced the Tungusic expansion north and across Siberia (as the vast territorries south and north of the Amur river were conquered and became part of the Mongol empire).

References

Achilli A, Perego UA, Lancioni H, Olivieri A, Gandini F, Hooshiar Kashani B, Battaglia V, Grugni V, Angerhofer N, Rogers MP, et al. 2013. Reconciling migration models to the Americas with the variation of North American native mitogenomes. Proc Natl Acad Sci U S A. 110:14308–14313.

Alekseeva TI, editor. 2002. Vostochnye slavjane: antropologicheskaja i etnicheskaja istorija [Eastern Slavs: anthropological and ethnic history]. Moscow: Nauchnyj mir.

Allentoft ME, Sikora M, Sjögren K-G, Rasmussen S, Rasmussen M, Stenderup J, Damgaard PB, Schroeder H, Ahlström T, Vinner L, et al. 2015. Population genomics of Bronze Age Eurasia. Nature. 522:167–172.

Andrés AM, Clark AG, Shimmin L, Boerwinkle E, Sing CF, Hixson JE. 2007. Understanding the accuracy of statistical haplotype inference with sequence data of known phase. Genet Epidemiol. 31:659–671.

Antropova VV, Kuznetsova VG. 1956. Chukchi. In: Levin MG, Potapov LP, editors. Narody Sibiri [Peoples of Siberia]. Moscow: Publisher of the Academy of Sciences of the USSR.

Balanovsky O, Rootsi S, Pshenichnov A, Kivisild T, Churnosov M, Evseeva I, Pocheshkhova E, Boldyreva M, Yankovsky N, Balanovska E, Villems R. 2008. Two Sources of the Russian Patrilineal Heritage in Their Eurasian Context. Am J Hum Genet. 82:236–250.

Barfield TJ. 2001. The shadow empires: imperial state formation along the Chinese-Nomad frontier. In: Alcock S, D'Altroy TN, Morrison KD, Sinopoli CM, editors. Empires: Perspectives from Archaeology and History. Cambridge: Cambridge University Press; p. 10–41.

Brisbin A, Bryc K, Byrnes J, Zakharia F, Omberg L, Degenhardt J, Reynolds A, Ostrer H, Mezey JG, Bustamante CD. 2012. PCAdmix: Principal Components-Based Assignment of Ancestry Along Each Chromosome in Individuals with Admixed Ancestry from Two or More Populations. Hum Biol. 84:343–364.

Browning SR, Browning BL. 2007. Rapid and accurate haplotype phasing and missing-data inference for whole-genome association studies by use of localized haplotype clustering. Am J Hum Genet. 81:1084–1097.

Cheboksarov NN, Bruk SI, Its RF, Stratanovich GG, editors. 1965. Narody Vostochnoj Asii [The Peoples of East Asia]. Moscow: Publisher of the Academy of Sciences of the USSR.

Crubézy E, Amory S, Keyser C, Bouakaze C, Bodner M, Gibert M, Röck A, Parson W, Alexeev A, Ludes B. 2010. Human evolution in Siberia: from frozen bodies to ancient DNA. BMC Evol Biol. 10:25.

Delaneau O, Coulonges C, Zagury J-F. 2008. Shape-IT: new rapid and accurate algorithm for haplotype inference. BMC Bioinformatics. 9:540.

Derenko M, Malyarchuk B, Denisova GA, Wozniak M, Dambueva I, Dorzhu C, Luzina F, Miścicka-Sliwka D, Zakharov I. 2006. Contrasting patterns of Y-chromosome variation in South Siberian populations from Baikal and Altai-Sayan regions. Hum Genet. 118:591–604.

Derenko MV, Grzybowski T, Malyarchuk BA, Dambueva IK, Denisova GA, Czarny J, Dorzhu CM,

Kakpakov VT, Miścicka-Sliwka D, Woźniak M, Zakharov IA. 2003. Diversity of mitochondrial DNA lineages in South Siberia. Ann Hum Genet. 67:391–411.

Derevianko AP, Clark GH. 1965. New Data on the Archaeology of the Middle Amur. Arct Anthropol. 3:136–141.

Dikov NN. 1977. Arheologicheskije pamjatniki Kamchatki, Chukotki i verhnej Kolymy [Archaeological Sites of Kamchatka, Chukotka and the Upper Kolyma]. Moscow: Nauka.

Dikov NN, Clark GH. 1965. The Stone Age of Kamchatka and the Chukchi Peninsula in the Light of New Archaeological Data. Arctic Anthropology. 3:10–25.

Dixon EJ. 2013. Arrows and atl atls: a guide to the archeology of Beringia. Anchorage: United States Department of the Interior.

Dolgih BO. 1957. Proishozhdenie nganasanov [Origins of the Nganasan]. In: Sibirskij Etnograficheskij Sbornik [Siberian Ethnographic Collection]. Vol. 1. Moscow: Academy of Sciences of the USSR.

Duggan AT, Whitten M, Wiebe V, Crawford M, Butthof A, Spitsyn V, Makarov S, Novgorodov I, Osakovsky V, Pakendorf B. 2013. Investigating the Prehistory of Tungusic Peoples of Siberia and the Amur-Ussuri Region with Complete mtDNA Genome Sequences and Y-chromosomal Markers. PLoS ONE. 8:e83570.

Goebel T, Slobodin SB, Waters MR. 2010. New dates from Ushki-1, Kamchatka, confirm 13,000 cal BP age for earliest Paleolithic occupation. J Archaeol Sci. 37:2640–2649.

González-Ruiz M, Santos C, Jordana X, Simón M, Lalueza-Fox C, Gigli E, Aluja MP, Malgosa A. 2012. Tracing the Origin of the East-West Population Admixture in the Altai Region (Central Asia). PLoS ONE. 7:e48904.

Haak W, Lazaridis I, Patterson N, Rohland N, Mallick S, Llamas B, Brandt G, Nordenfelt S, Harney E, Stewardson K, et al. 2015. Massive migration from the steppe was a source for Indo-European languages in Europe. Nature. 522:207–211.

Helimskij E. 2000. Komparativistika, Uralistika. Lekcii i Statji [Comparativism, Uralistics. Lectures and Articles]. Moscow: Jazyki Slavjanskoj Kultury [Languages of the Slavic Culture].

Hoffecker JF, Elias SA, O'Rourke DH. 2014. Out of Beringia? Science. 343:979–980.

International HapMap Consortium, Frazer KA, Ballinger DG, Cox DR, Hinds DA, Stuve LL, Gibbs RA, Belmont JW, Boudreau A, Hardenbol P, et al. 2007. A second generation human haplotype map of over 3.1 million SNPs. Nature. 449:851–861.

Ivanov SV, Levin MG, Smoljak AV. 1956. Nanaicy[Nanai]. In: Levin MG, Potapov LP, editors. Narody Sibiri [Peoples of Siberia]. Moscow: Publisher of the Academy of Sciences of the USSR.

Keyser-Tracqui C, Crubezy E, Ludes B. 2003. Nuclear and Mitochondrial DNA Analysis of a 2,000-Year-Old Necropolis in the Egyin Gol Valley of Mongolia. Am J Hum Genet. 73:247–260.

Khrunin AV, Khokhrin DV, Filippova IN, Esko T, Nelis M, Bebyakova NA, Bolotova NL, Klovins J, Nikitina-Zake L, Rehnström K, et al. 2013. A genome-wide analysis of populations from

European Russia reveals a new pole of genetic diversity in northern Europe. PloS One. 8:e58552.

Kim HJ. 2013. The Huns, Rome and the Birth of Europe. Cambridge : New York: Cambridge University Press.

Konovalov P. 1976. Khunnu v Zabaikal’e [The Xiongnu in the Trans-Baikal Region]. Ulan-Ude: Nauka.

Lao O, Lu TT, Nothnagel M, Junge O, Freitag-Wolf S, Caliebe A, Balascakova M, Bertranpetit J, Bindoff LA, Comas D, et al. 2008. Correlation between genetic and geographic structure in Europe. Curr Biol CB. 18:1241–1248.

Larsen LB, Vinther BM, Briffa KR, Melvin TM, Clausen HB, Jones PD, Siggaard-Andersen M-L, Hammer CU, Eronen M, Grudd H, et al. 2008. New ice core evidence for a volcanic cause of the A.D. 536 dust veil. Geophys Res Lett. 35:L04708.

Levin MG. 1956. Evens. In: Levin MG, Potapov LP, editors. Narody Sibiri [Peoples of Siberia]. Moscow: Publisher of the Academy of Sciences of the USSR.

Li C, Li H, Cui Y, Xie C, Cai D, Li W, Mair VH, Xu Z, Zhang Q, Abuduresule I, et al. 2010. Evidence that a West-East admixed population lived in the Tarim Basin as early as the early Bronze Age. BMC Biol. 8:15.

López Herráez D, Bauchet M, Tang K, Theunert C, Pugach I, Li J, Nandineni MR, Gross A, Scholz M, Stoneking M. 2009. Genetic Variation and Recent Positive Selection in Worldwide Human Populations: Evidence from Nearly 1 Million SNPs. PLoS ONE. 4:e7888.

McGhee R. 2001. Ancient People of the Arctic. Reprint ed. Vancouver, BC: University of Washington Press.

Menovschikov GA. 1956. Eskimosy [Eskimo people]. In: Levin MG, Potapov LP, editors. Narody Sibiri [Peoples of Siberia]. Moscow: Publisher of the Academy of Sciences of the USSR.

Miniaev S. 2001. Art and Archaeology of the Xiongnu: New Discoveries in Russia. Circle of Inner Asia Art Newsletter 14:3–9.

Mooder KP, Schurr TG, Bamforth FJ, Bazaliiski VI, Savel’ev NA. 2006. Population affinities of Neolithic Siberians: a snapshot from prehistoric Lake Baikal. Am J Phys Anthropol. 129:349–361.

Naumov IV. 2006. The History of Siberia. London: Routledge

Nikolaev RV. 2004. Prehistoric cultural links along the Yenisei: Revelations from a bronze idol. In: Vajda EJ, editor. Languages and Prehistory in Central Siberia. [Amsterdam]: John Benjamins Publishing Company; p. 257–262.

Novembre J, Johnson T, Bryc K, Kutalik Z, Boyko AR, Auton A, Indap A, King KS, Bergmann S, Nelson MR, et al. 2008. Genes mirror geography within Europe. Nature. 456:98–101.

Pakendorf B, Morar B, Tarskaia LA, Kayser M, Soodyall H, Rodewald A, Stoneking M. 2002. Y-chromosomal evidence for a strong reduction in male population size of Yakuts. Hum Genet. 110:198–200.

Pakendorf B, Novgorodov IN, Osakovskij VL, Danilova AP, Protod’jakonov AP, Stoneking M. 2006. Investigating the effects of prehistoric migrations in Siberia: genetic variation and the origins of Yakuts. Hum Genet. 120:334–353.

Pakendorf B, Novgorodov IN, Osakovskij VL, Stoneking M. 2007. Mating patterns amongst Siberian reindeer herders: inferences from mtDNA and Y-chromosomal analyses. Am J Phys Anthropol. 133:1013–1027.

Palamara PF, Lencz T, Darvasi A, Pe’er I. 2012. Length Distributions of Identity by Descent Reveal Fine-Scale Demographic History. Am J Hum Genet. 91:809–822.

Patterson NJ, Moorjani P, Luo Y, Mallick S, Rohland N, Zhan Y, Genschoreck T, Webster T, Reich D. 2012. Ancient Admixture in Human History. Genetics. 192(3):1065-93

Pickrell JK, Pritchard JK. 2012. Inference of Population Splits and Mixtures from Genome-Wide Allele Frequency Data. PLoS Genet. 8:e1002967.

Pletneva LM, editor. 1987. Smena kultur i migraciii v Zapadnoj Sibiri [Replacement of cultures and migrations in Western Siberia] Tomsk: Izdatelstvo TGU [Tomsk State University Publisher]

Popov AA. 1956a. Dolgans. In: Levin MG, Potapov LP, editors. Narody Sibiri [Peoples of Siberia]. Moscow: Publisher of the Academy of Sciences of the USSR.

Popov AA. 1956b. Nganasans. In: Levin MG, Potapov LP, editors. Narody Sibiri [Peoples of Siberia]. Moscow: Publisher of the Academy of Sciences of the USSR.

Popov AN, Tabarev AV. 2008. Neolithic Cultures of the Russian Far East. Turk Acad Sci J Archaeol. 11:21–34.

Pugach I, Matveyev R, Wollstein A, Kayser M, Stoneking M. 2011. Dating the age of admixture via wavelet transform analysis of genome-wide data. Genome Biol. 12:R19.

Raghavan M, DeGiorgio M, Albrechtsen A, Moltke I, Skoglund P, Korneliussen TS, Grønnow B, Appelt M, Gulløv HC, Friesen TM, et al. 2014. The genetic prehistory of the New World Arctic. Science. 345:1255832.

Raghavan M, Skoglund P, Graf KE, Metspalu M, Albrechtsen A, Moltke I, Rasmussen S, Stafford Jr TW, Orlando L, Metspalu E, et al. 2014. Upper Palaeolithic Siberian genome reveals dual ancestry of Native Americans. Nature. 505:87–91.

Rainey F, Ralph E. 1959. Radiocarbon Dating in the Arctic. American Antiquity. 24:365–374.

Rasmussen M, Li Y, Lindgreen S, Pedersen JS, Albrechtsen A, Moltke I, Metspalu M, Metspalu E, Kivisild T, Gupta R, et al. 2010. Ancient human genome sequence of an extinct Palaeo-Eskimo. Nature. 463:757–762.

Reich D, Patterson N, Campbell D, Tandon A, Mazieres S, Ray N, Parra MV, Rojas W, Duque C, Mesa N, et al. 2012a. Reconstructing Native American population history. Nature. 488:370–374.

Reich D, Patterson N, Campbell D, Tandon A, Mazieres S, Ray N, Parra MV, Rojas W, Duque C, Mesa N, et al. 2012b. Reconstructing Native American population history. Nature. 488:370–374.

Rudenko S. 1962. Kultura hunnov i noinulinkskie kurgany [Xiongnu Culture and Noin Ula kurgans]. Leningrad: Publisher of the Academy of Sciences of the USSR.

Savinov DG, Podolskiy ML, editors. 2006. Okunevskij Sbornik: Kultura i ee okruzhenie 2 [Okunev collection: Culture and its surroundings 2]. Saint Petersburg: SPbGU [Publisher of the Saint Petersburg State University].

Sinor D. 1990. The Cambridge History of Early Inner Asia. Cambridge: Cambridge University Press.

Sokolova LA. 2007. Okunev cultural tradition in the stratigraphic aspect. Archaeol Ethnol Anthropol Eurasia. 30:41–51.

Solodovnikov KN. 2003. Materialy k antropologii afanasievskoj kultury [Materials to anthropology of Afanasevo culture]. Drevnosti Altaja [Antiquities of Altai]. 10:3–27.

Stapert E. 2013. Contact-induced change in Dolgan: an investigation into the role of linguistic data for the reconstruction of a people's (pre)history. PhD Dissertation. Utrecht: LOT.

Tamm E, Kivisild T, Reidla M, Metspalu M, Smith DG, Mulligan CJ, Bravi CM, Rickards O, Martinez-Labarga C, Khusnutdinova EK, et al. 2007. Beringian Standstill and Spread of Native American Founders. PLoS ONE. 2:e829.

Tang H, Coram M, Wang P, Zhu X, Risch N. 2006. Reconstructing genetic ancestry blocks in admixed individuals. Am J Hum Genet. 79:1–12.

Taskin V. 1968. Materialy po istorii siunnu. (Po kitaiskim istochnikam) [Materials on the history of Xiongnu. (According to Chinese sources)]. Moscow: Nauka.

Tokarev SA, Gurvich IS. 1956. Yakuty [Yakuts]. In: Levin MG, Potapov LP, editors. Narody Sibiri [Peoples of Siberia]. Moscow: Publisher of the Academy of Sciences of the USSR.

Tugolukov VA. 1980. Ėtničeskie korni tungusov [Ethnic roots of the Tungus people]. In: Gurvich IS, editor. Ėtnogenez Narodov Severa [Ethnogenesis of the Peoples of the North]. Moscow: Nauka; p. 152–176.

Tumen D. 2008. Anthropology of Archaeological populations from Inner Asia. Mong J Anthropol Ethnol. 4:162–183.

Vajda EJ. 2009. The Languages of Siberia. Language and Linguistics Compass. 3:424–440.

Vasilevič G. 1969. Ėvenki. Istoriko-ėtnografičeskie očerki (XVIII-načalo XX v.) [Evenks. Historico-ethnographic sketches (XVIII-beg of the XXC)]. Leningrad: Nauka

Vasilevich GM. 1956. Evenks. In: Levin MG, Potapov LP, editors. Narody Sibiri [Peoples of Siberia]. Moscow: Publisher of the Academy of Sciences of the USSR.

Vasil'ev SA, Kuzmin YV, Orlova LA, Dementiev VN. 2002. Radiocarbon-based chronology of the Paleolithic in Siberia and its relevance to the peopling of the New World. Radiocarbon. 44:503–530.

Vasiliev VI. 1974. K probleme etnogeneza severosamodijskih narodov [On the question of ethnogenesis of the Samoyedic peoples of the North]. In: Gurvich IS, editor. Socialnaja organizacija

i kultura narodov severa [Social organization and culture of the peoples of the North]. Moscow: Nauka; p. 133–175.

Weber AW, Bettinger R. 2010. Middle Holocene hunter-gatherers of Cis-Baikal, Siberia: An overview for the new century. J Anthropol Archaeol. 29:491–506.

Zlojutro M, Tarskaia LA, Sorensen M, Snodgrass JJ, Leonard WR, Crawford MH. 2009. Coalescent simulations of Yakut mtDNA variation suggest small founding population. Am J Phys Anthropol. 139:474–482.

Table S1 **Populations included in the study**

Populations are grouped by linguistic affiliation

population	subgroup	N	subsistence	provenance	latitude and longitude coords
Turkic					
Altaians		12	pastoralist (horse/cattle)	Rasmussen et al 2010	51.57N 85.59E (Gorno-Altaisk)
Tuvans		16	pastoralist (horse/cattle)	Rasmussen et al 2010	51.43N 94.27E (Kyzyl)
Yakuts			pastoralist (horse/cattle)		
	CEPH	24		Li et al 2008	62.98N 129.50E
	Verhoyansk	8		this study	68.40N 136.14E
	not specified	13		Reich et al 2012	
Dolgans			H-G/ reindeer herder		
	Taimyr	10		this study	69.21N 86.13E
	Anabar	10		this study	72.48N 113.14E
Tungusic-Manchu					
N.Tungusic					
Evens			H-G/ reindeer herder	this study	
	Sakkyryyr	7			67.48N 130.24E
	Sebjan	10			64.24N 126.16E
	Tompo	10			64.39N 135.57E
	Berezovka	6			67.30N 154.40E
	Kamchatka	5			55.55N 158.41E (Esso)
Evenks			H-G/ reindeer herder	this study	
	Taimyr	5			68.18N

					88.45E (Khantayskoye Lake)
	Stony-Tunguska	6			61.47N 93.35E
Oroqen		10		Li et al 2008	50.43N 126.50E
S.Tungusic					
Hezhen		9		Li et al 2008	47.50N 133.50E
Manchu					
Xibo		9		Li et al 2008	43.50N 81.50E
Mongolic					
Mongolians		10	pastoralist (horse/cattle)	Li et al 2008	45N 111E
Buryats		19	pastoralist (horse/cattle)	Rasmussen et al 2010	51.50N 107.36E
Daur		9		Li et al 2008	48.50N 124.00E
Tu Mongolians		10		Li et al 2008	36N 101E
Uralic					
N.Samoyedic					
Nganasan			H-G		
		10		Rasmussen et al 2010	71.60N 92.49E (Ust-Avam)
		14		Reich et al 2012	n/a
Nenets			reindeer herder		
	Taimyr	6		this study	69.21N 86.13E
	Yamal	6		this study	69.42N 70.36E
	not specified	3		Reich et al 2012	n/a
S.Samoyedic					
Selkup		9	H-G/ reindeer herder	Rasmussen et al 2010	65.42N 82.27E (Krasnoselkup)
Ugric					
Khanty		35		Reich et al	61N

				2012	69E (Khanty-Mansiysk, Ugry)
Chukotko-Kamchatkan					
Chukchi			H-G/ reindeer herder		
		9		Rasmussen et al 2010	64.44N 177.30E (Anadyr)
		19		Reich et al 2012	n/a
Koryaks			H-G/ reindeer herder	Rasmussen et al 2010	55.55N 158.41E (Palana)
Eskimo-Aleut					
Yupik					
Naukan Yupik		16		Reich et al 2012	64.30N -173.01E (Novoe Chaplino)
Inuit					
Greenland				Reich et al 2012	
	east	7			65.38N -37.40E (Tasiliak)
	west	8			64.10N -51.43E (Nuuk)
Aleut					
Aleuts		8		Reich et al 2012	53.40N -166.38E (Unalaska island)
isolate language					
Yukaghirs		13		Reich et al 2012	68.52N 161.27E (upper Kolyma River)
Yeniseic					
Kets		2		Rasmussen et al 2010	62.29N 86.17E (Kellog)
Indo-European					

Slavic					
Russians		25		Li et al 2008	61N<br>40E
Romance					
French		29		Li et al 2008	46N<br>2E
Italian				Li et al 2008	
	Bergamo	13			46N<br>10E
	Tuscan	8			43.48N<br>11.14E
Sino-Tibetan					
Han Chinese		44		Li et al 2008	32.27N<br>114.00E
Japonic					
Japanese		28		Li et al 2008	38N<br>138E

Table S2. **Results of f3 test for admixture**

f3(C; A, B)	f3	SE	Z-score
Mongolians			
Mongolians;Nenets,Han	-0.00203283	7.56683E-005	-26.865
Mongolians;Han,Dolgans	-0.0016677	6.57616E-005	-25.3597
Mongolians;Han,Nganasan	-0.00205561	8.15037E-005	-25.2211
Mongolians;Khanty,Han	-0.00194514	7.73164E-005	-25.1582
Mongolians;Han,Evens	-0.00177316	0.000070515	-25.1458
Mongolians;Han,Yakuts	-0.00148198	6.25567E-005	-23.6902
Mongolians;Evenks,Han	-0.00190545	8.14375E-005	-23.3976
Mongolians;Evens_Kamchatka,Han	-0.00180648	7.99792E-005	-22.5869
Mongolians;Selkup,Han	-0.00185174	8.36972E-005	-22.1243
Mongolians;Han,Buryats	-0.00124601	0.000063368	-19.6631
Mongolians;Chukchi,Han	-0.00150967	7.70974E-005	-19.5813
Mongolians;Tuvans,Han	-0.00128528	6.68877E-005	-19.2154
Mongolians;Khanty,Japanese	-0.00155012	8.21946E-005	-18.8591
Mongolians;Russians,Han	-0.00192452	0.000102687	-18.7416
Mongolians;Altaians,Han	-0.00129863	6.94591E-005	-18.6963
Mongolians;Russians,Japanese	-0.00198237	0.000106664	-18.5853
Mongolians;Nenets,Japanese	-0.00149648	8.20408E-005	-18.2407
Mongolians;French,Japanese	-0.00199481	0.000112454	-17.7389
Mongolians;Aleutian,Han	-0.00171328	9.66813E-005	-17.7209
Mongolians;French,Han	-0.00188307	0.000108547	-17.3479
Mongolians;Italy,Japanese	-0.0019752	0.000114607	-17.2346
Mongolians;Han,Greenland_Inuits	-0.0015429	9.08236E-005	-16.9879
Mongolians;Koryaks,Han	-0.00151785	9.05881E-005	-16.7555
Mongolians;Han,Kets	-0.00192594	0.000115468	-16.6794
Mongolians;Italy,Han	-0.0018147	0.000110797	-16.3786
Mongolians;Selkup,Japanese	-0.00142815	8.91079E-005	-16.0272
Mongolians;Aleutian,Japanese	-0.00162987	0.000101934	-15.9895
Mongolians;Naukan_Yupik,Han	-0.00144171	0.000092451	-15.5943
Mongolians;Nganasan,Japanese	-0.00132159	9.03759E-005	-14.6232
Mongolians;Dolgans,Japanese	-0.00105656	7.31569E-005	-14.4424
Mongolians;Japanese,Yakuts	-0.000947684	6.76492E-005	-14.0088
Mongolians;Altaians,Japanese	-0.00102939	7.48561E-005	-13.7516
Mongolians;Evens_Kamchatka,Tu	-0.00122229	9.46059E-005	-12.9198
Mongolians;Evenks,Japanese	-0.00112849	8.80864E-005	-12.8112
Mongolians;Evenks,Tu	-0.00122121	9.64571E-005	-12.6607
Mongolians;Tu,Evens	-0.00108609	8.59093E-005	-12.6423
Mongolians;Japanese,Evens	-0.00098192	0.000078891	-12.4465
Mongolians;Tu,Nganasan	-0.00123442	0.000100951	-12.2279
Mongolians;French,Hezhen	-0.00154933	0.000127924	-12.1113
Mongolians;Kets,Japanese	-0.00150607	0.000125018	-12.0468
Mongolians;Japanese,Buryats	-0.000817357	6.82116E-005	-11.9827
Mongolians;Tuvans,Japanese	-0.000855717	7.16284E-005	-11.9466
Mongolians;Hezhen,Russians	-0.00137954	0.000123323	-11.1864
Mongolians;Italy,Hezhen	-0.00146874	0.00013178	-11.1454
Mongolians;Evens_Kamchatka,Japanese	-0.000911516	8.99939E-005	-10.1286
Mongolians;Greenland_Inuits,Japanese	-0.00101713	0.000101462	-10.0247
Mongolians;Tu,Dolgans	-0.000804938	0.000082479	-9.75931
Mongolians;Italy,Oroqen	-0.00134387	0.000140163	-9.58796
Mongolians;French,Oroqen	-0.00129371	0.000137547	-9.40564
Mongolians;Oroqen,Han	-0.000704691	7.59584E-005	-9.27733
Mongolians;Tu,Yakuts	-0.000683798	7.81151E-005	-8.75372

Mongolians;Chukchi,Japanese	-0.000771071	8.83725E-005	-8.72523
Mongolians;Aleutian,Hezhen	-0.000986496	0.00011622	-8.4882
Mongolians;Tu,Nenets	-0.0007909	9.50468E-005	-8.32117
Mongolians;Russians,Daur	-0.0010172	0.000122783	-8.28453
Mongolians;French,Daur	-0.00109001	0.000132857	-8.20437
Mongolians;Tu,Chukchi	-0.000785463	9.59923E-005	-8.18256
Mongolians;Italy,Daur	-0.00106667	0.000135255	-7.88635
Mongolians;Tu,Buryats	-0.000577541	7.38921E-005	-7.81601
Mongolians;Russians,Oroqen	-0.000996154	0.000129938	-7.66639
Mongolians;San,Japanese	-0.000973248	0.000131143	-7.42128
Mongolians;Oroqen,San	-0.00110598	0.000152776	-7.2392
Mongolians;Tu,Oroqen	-0.000599674	8.30777E-005	-7.21823
Mongolians;Aleutian,Daur	-0.000829078	0.00011521	-7.19627
Mongolians;Koryaks,Japanese	-0.000708176	9.85686E-005	-7.1846
Mongolians;Naukan_Yupik,Japanese	-0.000723799	0.000101859	-7.10589
Mongolians;Han,San	-0.000868338	0.000122565	-7.08474
Mongolians;Italy,Evens_Kamchatka	-0.00109967	0.00016207	-6.78519
Mongolians;Evens_Kamchatka,French	-0.00105881	0.000160365	-6.60254
Mongolians;Tu,Koryaks	-0.000766038	0.000116219	-6.59132
Mongolians;Hezhen,San	-0.000973257	0.000155205	-6.2708
Mongolians;Tu,Tuvans	-0.000480562	7.96363E-005	-6.03445
Mongolians;Xibo,Nganasan	-0.000603146	0.000102442	-5.8877
Mongolians;Evens_Kamchatka,San	-0.00106162	0.000187429	-5.66409
Mongolians;Tu,Naukan_Yupik	-0.000637049	0.000112914	-5.64189
Mongolians;Daur,San	-0.000804923	0.000148127	-5.43402
Mongolians;Aleutian,Oroqen	-0.000657193	0.000125696	-5.22843
Mongolians;Tu,Selkup	-0.000496559	0.000102453	-4.84672
Mongolians;Oroqen,Japanese	-0.000373939	7.75205E-005	-4.82375
Mongolians;Tu,Khanty	-0.000452478	9.73107E-005	-4.64983
Mongolians;Khanty,Hezhen	-0.000465668	0.00010139	-4.59285
Mongolians;Nenets,Xibo	-0.000435343	9.48764E-005	-4.58853
Mongolians;Khanty,Daur	-0.000447952	0.000097856	-4.57766
Mongolians;Tu,Greenland_Inuits	-0.00046398	0.000110374	-4.20371
Mongolians;Evens_Kamchatka,Xibo	-0.000370526	9.99465E-005	-3.70725
Mongolians;Han,Daur	-0.000279092	7.60835E-005	-3.66823
Mongolians;Tu,Altaians	-0.000304437	8.35248E-005	-3.64487
Mongolians;Tu,Kets	-0.000487472	0.000137411	-3.54754
Mongolians;Xibo,Evens	-0.000304361	8.94783E-005	-3.4015
Mongolians;Evenks,Xibo	-0.000337556	9.93035E-005	-3.39924
Mongolians;Xibo,Buryats	-0.000259739	7.76769E-005	-3.34384
Mongolians;Evenks,Italy	-0.000525914	0.000161345	-3.25955
Mongolians;Tu,Daur	-0.000262449	8.21467E-005	-3.19489
Mongolians;Daur,Greenland_Inuits	-0.000336688	0.000109379	-3.07818

Tuvans

Tuvans;Evenks,French	-0.00351823	0.000132461	-26.5606
Tuvans;Evenks,Italy	-0.00349685	0.000133588	-26.1763
Tuvans;Evens_Kamchatka,French	-0.00352153	0.000138618	-25.4045
Tuvans;Italy,Evens_Kamchatka	-0.00344188	0.000138419	-24.8657
Tuvans;French,Evens	-0.00280722	0.000116858	-24.0224
Tuvans;Italy,Evens	-0.00274746	0.000117941	-23.2951
Tuvans;Evenks,Russians	-0.00279317	0.000122864	-22.7338
Tuvans;Evens_Kamchatka,Russians	-0.00278437	0.000129177	-21.5547
Tuvans;Italy,Nganasan	-0.00292947	0.000138987	-21.0773
Tuvans;French,Nganasan	-0.00285078	0.000139116	-20.4922
Tuvans;Russians,Evens	-0.00215977	0.000109195	-19.7791

Tuvans;French,Oroqen	-0.00249565	0.000127756	-19.5346
Tuvans;Russians,Oroqen	-0.00215705	0.000117628	-18.3379
Tuvans;Italy,Oroqen	-0.0024253	0.000132671	-18.2805
Tuvans;French,Hezhen	-0.00209101	0.000132875	-15.7367
Tuvans;Russians,Nganasan	-0.00201938	0.000131374	-15.3712
Tuvans;Hezhen,Russians	-0.00188018	0.000124547	-15.0962
Tuvans;Russians,Japanese	-0.00166071	0.000111466	-14.8989
Tuvans;French,Japanese	-0.00171419	0.000120901	-14.1785
Tuvans;Italy,Hezhen	-0.00188992	0.000135434	-13.9545
Tuvans;Evenks,San	-0.00207509	0.000153857	-13.4871
Tuvans;Evenks,Aleutian	-0.00161465	0.000122294	-13.203
Tuvans;Italy,Japanese	-0.00157407	0.000123312	-12.7649
Tuvans;Russians,Daur	-0.00154407	0.000125179	-12.3349
Tuvans;French,Daur	-0.00165793	0.000136472	-12.1485
Tuvans;San,Nganasan	-0.00191945	0.00016131	-11.8991
Tuvans;Evens_Kamchatka,San	-0.0019772	0.000172489	-11.4627
Tuvans;Italy,Daur	-0.00151408	0.000137762	-10.9906
Tuvans;Russians,Han	-0.0011733	0.000115588	-10.1506
Tuvans;Aleutian,Oroqen	-0.00117208	0.00011621	-10.0859
Tuvans;San,Evens	-0.00138588	0.000138232	-10.0257
Tuvans;Evens_Kamchatka,Aleutian	-0.00123093	0.000127534	-9.65182
Tuvans;Aleutian,Evens	-0.00102204	0.000106484	-9.59808
Tuvans;Nenets,Han	-0.000772016	8.27826E-005	-9.32581
Tuvans;French,Han	-0.0011729	0.000125862	-9.31889
Tuvans;Aleutian,Nganasan	-0.00113424	0.000126345	-8.97729
Tuvans;Nenets,Japanese	-0.000665226	8.31508E-005	-8.00023
Tuvans;Italy,Han	-0.000984016	0.000126241	-7.79472
Tuvans;Khanty,Japanese	-0.000670809	8.69862E-005	-7.71167
Tuvans;Khanty,Han	-0.000636269	8.72601E-005	-7.29164
Tuvans;Selkup,Han	-0.000615378	8.89051E-005	-6.92174
Tuvans;Selkup,Japanese	-0.000621345	9.02605E-005	-6.88391
Tuvans;Aleutian,Hezhen	-0.000841124	0.000122282	-6.87856
Tuvans;Aleutian,Daur	-0.000709945	0.000118949	-5.96848
Tuvans;Aleutian,Japanese	-0.0006622	0.00011155	-5.93637
Tuvans;Oroqen,San	-0.000760781	0.000142135	-5.35252
Tuvans;Han,Nganasan	-0.000458496	8.98503E-005	-5.10289
Tuvans;Italy,Koryaks	-0.000813264	0.000165402	-4.91689
Tuvans;Tu,Nganasan	-0.000442015	9.98403E-005	-4.42722
Tuvans;Mongolians,Russians	-0.000534055	0.000120857	-4.41889
Tuvans;Kets,Japanese	-0.000543921	0.00012361	-4.40029
Tuvans;Khanty,Daur	-0.000417179	9.53389E-005	-4.37575
Tuvans;Mongolians,French	-0.000575102	0.000131962	-4.35808
Tuvans;Han,Kets	-0.000534226	0.000124328	-4.29692
Tuvans;Khanty,Hezhen	-0.000408656	9.87608E-005	-4.13783
Tuvans;French,Koryaks	-0.000681166	0.000165925	-4.10526
Tuvans;Tu,Nenets	-0.000334802	9.04636E-005	-3.70096
Tuvans;Italy,Mongolians	-0.000454592	0.000133176	-3.41346
Tuvans;Selkup,Daur	-0.00032485	9.70553E-005	-3.34706

Khanty

Khanty;French,Nganasan	-0.00309992	0.000131899	-23.5023
Khanty;Italy,Nganasan	-0.00296911	0.000137866	-21.5362
Khanty;Evenks,French	-0.00276222	0.000136118	-20.2929
Khanty;Evens_Kamchatka,French	-0.0025904	0.000137743	-18.806
Khanty;Evenks,Italy	-0.00253134	0.000141532	-17.8853
Khanty;Russians,Nganasan	-0.00215548	0.000123171	-17.5

Khanty;Italy,Evens_Kamchatka	-0.00230126	0.000143217	-16.0684
Khanty;French,Evens	-0.00192615	0.000124298	-15.4963
Khanty;Evenks,Russians	-0.00192413	0.000126481	-15.2127
Khanty;Evens_Kamchatka,Russians	-0.00174022	0.000125799	-13.8333
Khanty;Italy,Evens	-0.0016569	0.000129311	-12.8133
Khanty;Russians,Evens	-0.00116568	0.000115684	-10.0764
Khanty;French,Koryaks	-0.00100941	0.000145117	-6.9558
Khanty;Italy,Koryaks	-0.000932012	0.000149789	-6.22217
Khanty;French,Chukchi	-0.000761716	0.000131463	-5.79414
Khanty;Italy,Chukchi	-0.000636771	0.000136046	-4.68057
Khanty;French,Oroqen	-0.000612089	0.000133443	-4.5869

Nenets

Nenets;French,Nganasan	-0.00524027	0.000122877	-42.6463
Nenets;Russians,Nganasan	-0.00465283	0.000111236	-41.8286
Nenets;Italy,Nganasan	-0.00512914	0.000125712	-40.8008
Nenets;Evenks,French	-0.00429488	0.00014028	-30.6164
Nenets;Evenks,Russians	-0.00381379	0.000129984	-29.3404
Nenets;Aleutian,Nganasan	-0.00318487	0.000111437	-28.58
Nenets;Evenks,Italy	-0.00408368	0.000145663	-28.0352
Nenets;Khanty,Nganasan	-0.00198011	8.57176E-005	-23.1004
Nenets;Evens_Kamchatka,French	-0.00325128	0.000152623	-21.3027
Nenets;Evens_Kamchatka,Russians	-0.0027581	0.000139876	-19.7182
Nenets;Italy,Evens_Kamchatka	-0.00298182	0.000158093	-18.8612
Nenets;French,Evens	-0.00257113	0.000136933	-18.7765
Nenets;Russians,Evens	-0.00216766	0.000126229	-17.1725
Nenets;Italy,Evens	-0.00232156	0.000142821	-16.2551
Nenets;Evenks,Aleutian	-0.00205245	0.000131108	-15.6547
Nenets;San,Nganasan	-0.00241856	0.000157139	-15.3912
Nenets;Evenks,Khanty	-0.00137243	8.99127E-005	-15.264
Nenets;Khanty,Evens	-0.00048475	8.75049E-005	-5.53969
Nenets;Evenks,San	-0.000961374	0.00017473	-5.50207
Nenets;Evens_Kamchatka,Khanty	-0.000500646	0.00010012	-5.00045
Nenets;Evens_Kamchatka,Aleutian	-0.000621837	0.00014076	-4.41772
Nenets;Selkup,Nganasan	-0.000478683	0.000111974	-4.27495
Nenets;French,Oroqen	-0.000569196	0.000157751	-3.60818
Nenets;Aleutian,Evens	-0.000447105	0.000125848	-3.55274
Nenets;Russians,Oroqen	-0.000474564	0.000147409	-3.21936
Nenets;French,Koryaks	-0.000556638	0.000177262	-3.1402

Altaians

Altaians;French,Evens	-0.00500369	0.000117878	-42.4479
Altaians;Evens_Kamchatka,French	-0.00565037	0.000135286	-41.7661
Altaians;Italy,Evens	-0.00496285	0.000120292	-41.2567
Altaians;Italy,Evens_Kamchatka	-0.00558964	0.000136665	-40.9004
Altaians;Evenks,French	-0.00543329	0.000133716	-40.6331
Altaians;Evenks,Italy	-0.00543083	0.000135753	-40.0053
Altaians;French,Oroqen	-0.00486176	0.000124785	-38.9612
Altaians;Italy,Oroqen	-0.00481033	0.000129285	-37.2071
Altaians;Russians,Evens	-0.00404102	0.000109668	-36.8476
Altaians;Russians,Japanese	-0.00399195	0.000110044	-36.276
Altaians;Evens_Kamchatka,Russians	-0.00459799	0.000126775	-36.269
Altaians;French,Hezhen	-0.00469486	0.000129505	-36.2523
Altaians;Evenks,Russians	-0.004393	0.000122064	-35.9893
Altaians;Russians,Oroqen	-0.00420793	0.000117228	-35.8952
Altaians;French,Nganasan	-0.00480721	0.000136257	-35.2806

Altaians;French,Japanese	-0.00436066	0.000123873	-35.2025
Altaians;Italy,Nganasan	-0.00490482	0.000140398	-34.935
Altaians;Hezhen,Russians	-0.0041688	0.000120744	-34.526
Altaians;Italy,Japanese	-0.00423946	0.000124837	-33.96
Altaians;Italy,Hezhen	-0.00451268	0.000132921	-33.9502
Altaians;Russians,Han	-0.00366486	0.000112513	-32.5728
Altaians;French,Han	-0.00397968	0.000124304	-32.0157
Altaians;French,Daur	-0.00427533	0.000136268	-31.3745
Altaians;Russians,Daur	-0.00384625	0.000122998	-31.271
Altaians;Italy,Han	-0.00380972	0.000124512	-30.5971
Altaians;Italy,Daur	-0.0041504	0.000139274	-29.8002
Altaians;Russians,Nganasan	-0.00366058	0.000132179	-27.6941
Altaians;Mongolians,French	-0.00339524	0.000127479	-26.6338
Altaians;Mongolians,Russians	-0.00303897	0.000115467	-26.319
Altaians;Italy,Mongolians	-0.00329365	0.000129296	-25.4738
Altaians;French,Yakuts	-0.00273741	0.000110574	-24.7564
Altaians;Xibo,Russians	-0.00290007	0.000118185	-24.5384
Altaians;Xibo,French	-0.00319801	0.000133248	-24.0004
Altaians;Italy,Yakuts	-0.00269055	0.00011225	-23.9693
Altaians;Aleutian,Hezhen	-0.00262792	0.00011488	-22.8753
Altaians;Aleutian,Oroqen	-0.00272113	0.000119645	-22.7434
Altaians;Italy,Xibo	-0.00307438	0.00013547	-22.6942
Altaians;Aleutian,Japanese	-0.00249161	0.000111971	-22.2524
Altaians;Tu,Russians	-0.00252214	0.000114104	-22.1038
Altaians;Russians,Yakuts	-0.00219702	0.000100461	-21.8693
Altaians;Italy,Koryaks	-0.00348184	0.000160449	-21.7005
Altaians;Tu,French	-0.00271871	0.00012554	-21.6561
Altaians;Italy,Chukchi	-0.00302674	0.000140569	-21.532
Altaians;Evenks,Aleutian	-0.00271266	0.000127741	-21.2355
Altaians;Aleutian,Daur	-0.00251029	0.000118909	-21.111
Altaians;French,Chukchi	-0.00292327	0.000139393	-20.9714
Altaians;Aleutian,Evens	-0.00240146	0.00011493	-20.895
Altaians;French,Koryaks	-0.00333082	0.000161036	-20.6836
Altaians;Aleutian,Han	-0.00230578	0.000114191	-20.1922
Altaians;Italy,Tu	-0.0025307	0.000127991	-19.7725
Altaians;Evens_Kamchatka,Aleutian	-0.00254272	0.000131816	-19.2899
Altaians;French,Buryats	-0.00234523	0.000123419	-19.0022
Altaians;Russians,Buryats	-0.00204242	0.000113046	-18.0671
Altaians;Italy,Buryats	-0.00225501	0.000125699	-17.9398
Altaians;French,Dolgans	-0.00216107	0.000122458	-17.6474
Altaians;Italy,Dolgans	-0.00216399	0.000125284	-17.2727
Altaians;Aleutian,Nganasan	-0.00227361	0.000131721	-17.2608
Altaians;Evenks,San	-0.00293266	0.000171259	-17.1241
Altaians;San,Evens	-0.00252486	0.000149584	-16.8792
Altaians;Evens_Kamchatka,San	-0.00304855	0.000181448	-16.8012
Altaians;Mongolians,Aleutian	-0.00189113	0.000113439	-16.6709
Altaians;San,Nganasan	-0.00281838	0.000171099	-16.4723
Altaians;Khanty,Han	-0.00142292	9.14215E-005	-15.5644
Altaians;Russians,Koryaks	-0.00238961	0.000153839	-15.5332
Altaians;Aleutian,Xibo	-0.00178091	0.000119003	-14.9653
Altaians;Russians,Chukchi	-0.00193878	0.000135384	-14.3206
Altaians;Italy,Tuvans	-0.00171226	0.000121613	-14.0796
Altaians;Khanty,Japanese	-0.00129714	9.21853E-005	-14.0711
Altaians;Tu,Aleutian	-0.001549	0.000110372	-14.0344
Altaians;Italy,Naukan_Yupik	-0.00223335	0.000160011	-13.9574
Altaians;Tuvans,French	-0.00169334	0.000121922	-13.8887

Altaians;Russians,Dolgans	-0.00154918	0.000113536	-13.6448
Altaians;Oroqen,San	-0.0020694	0.000152087	-13.6067
Altaians;Aleutian,Yakuts	-0.00136869	0.000101982	-13.4208
Altaians;Naukan_Yupik,French	-0.00210288	0.000160579	-13.0956
Altaians;Tuvans,Russians	-0.00137812	0.000111251	-12.3874
Altaians;Aleutian,Buryats	-0.00121766	0.00010681	-11.4002
Altaians;Khanty,Daur	-0.00101445	9.76469E-005	-10.389
Altaians;Khanty,Hezhen	-0.000992374	9.83124E-005	-10.0941
Altaians;Selkup,Han	-0.00105486	0.000104842	-10.0614
Altaians;Tu,Khanty	-0.000924459	9.61424E-005	-9.61551
Altaians;Hezhen,San	-0.00151416	0.000160884	-9.4115
Altaians;San,Yakuts	-0.00119366	0.00012856	-9.28489
Altaians;Koryaks,San	-0.00174034	0.000197413	-8.81571
Altaians;Daur,San	-0.00138562	0.000159328	-8.69667
Altaians;Khanty,Xibo	-0.00081402	9.46432E-005	-8.60093
Altaians;Mongolians,Khanty	-0.000776418	9.07658E-005	-8.55408
Altaians;Selkup,Japanese	-0.000900512	0.000107266	-8.39512
Altaians;Aleutian,Tuvans	-0.000876287	0.00010484	-8.35836
Altaians;Naukan_Yupik,Russians	-0.00118128	0.000148577	-7.95062
Altaians;Chukchi,San	-0.00136385	0.000175433	-7.77421
Altaians;Aleutian,Dolgans	-0.00080562	0.000109464	-7.3597
Altaians;Han,Kets	-0.000993628	0.000142809	-6.95772
Altaians;Nenets,Han	-0.000655228	0.000095421	-6.86671
Altaians;Tu,Selkup	-0.000693875	0.000108913	-6.37093
Altaians;Dolgans,San	-0.000854115	0.000139009	-6.14431
Altaians;Kets,Japanese	-0.000843004	0.000143379	-5.87955
Altaians;San,Buryats	-0.000742083	0.000133053	-5.57737
Altaians;Selkup,Daur	-0.000574954	0.000106297	-5.40895
Altaians;Xibo,Selkup	-0.000583874	0.000110119	-5.30222
Altaians;Khanty,Oroqen	-0.000531569	0.000100766	-5.27528
Altaians;Mongolians,San	-0.000790618	0.000156113	-5.06438
Altaians;Tuvans,San	-0.000635852	0.000131458	-4.83693
Altaians;Mongolians,Selkup	-0.000501753	0.000104878	-4.78417
Altaians;San,Japanese	-0.000734474	0.000165585	-4.43563
Altaians;Hezhen,Selkup	-0.000479999	0.000113389	-4.23321
Altaians;Tu,Nenets	-0.000407495	0.000101035	-4.03322
Altaians;Nenets,Japanese	-0.000388119	9.79856E-005	-3.96098
Altaians;Xibo,Kets	-0.000570184	0.000146337	-3.89639
Altaians;Naukan_Yupik,San	-0.000773558	0.000203128	-3.80824
Altaians;Tu,Kets	-0.000549359	0.000147531	-3.72369
Altaians;Xibo,San	-0.000514806	0.000155595	-3.30863

Russians

Russians;French,Nganasan	-0.00239342	8.98763E-005	-26.6301
Russians;French,Chukchi	-0.00223128	8.40083E-005	-26.5602
Russians;French,Evens	-0.00220945	8.47828E-005	-26.0602
Russians;Evens_Kamchatka,French	-0.00229917	9.23615E-005	-24.8931
Russians;Nenets,French	-0.00180598	7.26092E-005	-24.8727
Russians;Evenks,French	-0.00228708	9.23236E-005	-24.7724
Russians;French,Dolgans	-0.00185867	7.57343E-005	-24.5419
Russians;Naukan_Yupik,French	-0.00216838	8.95659E-005	-24.2099
Russians;Khanty,French	-0.00144898	6.01923E-005	-24.0725
Russians;French,Yakuts	-0.00178718	7.59756E-005	-23.5231
Russians;French,Koryaks	-0.002188	9.34876E-005	-23.4041
Russians;Italy,Khanty	-0.00144658	6.50019E-005	-22.2544
Russians;Italy,Nganasan	-0.00226021	0.000101742	-22.215

Russians;Italy,Nenets	-0.0017839	8.05844E-005	-22.1371
Russians;French,Oroqen	-0.00190062	8.62346E-005	-22.0401
Russians;Italy,Chukchi	-0.00210393	9.86646E-005	-21.3241
Russians;Tuvans,French	-0.00156201	7.36328E-005	-21.2135
Russians;French,Selkup	-0.00145558	6.94871E-005	-20.9474
Russians;Italy,Evens	-0.0019378	9.42549E-005	-20.5591
Russians;Italy,Koryaks	-0.0021082	0.000104387	-20.1959
Russians;French,Daur	-0.00167587	8.30573E-005	-20.1772
Russians;Evenks,Italy	-0.00205379	0.000101919	-20.1513
Russians;French,Hezhen	-0.00177284	8.82081E-005	-20.0984
Russians;French,Buryats	-0.0015496	7.71543E-005	-20.0845
Russians;Italy,Naukan_Yupik	-0.00206803	0.0001042	-19.8468
Russians;Italy,Evens_Kamchatka	-0.00200761	0.000101227	-19.8327
Russians;French,Greenland_Inuits	-0.00143428	7.54856E-005	-19.0007
Russians;Italy,Selkup	-0.00152998	8.07796E-005	-18.9402
Russians;Italy,Dolgans	-0.00163077	8.68376E-005	-18.7796
Russians;Mongolians,French	-0.00160306	8.59181E-005	-18.658
Russians;French,Han	-0.00156161	8.37818E-005	-18.639
Russians;French,Japanese	-0.00161549	8.67964E-005	-18.6124
Russians;Xibo,French	-0.00154472	8.50306E-005	-18.1667
Russians;French,Altaians	-0.00124678	7.03659E-005	-17.7186
Russians;Tu,French	-0.00144335	8.36223E-005	-17.2604
Russians;Italy,Yakuts	-0.0015095	8.86679E-005	-17.0242
Russians;French,Kets	-0.00156912	9.58763E-005	-16.3661
Russians;Italy,Oroqen	-0.00161837	9.92689E-005	-16.3029
Russians;Italy,Tuvans	-0.00135011	8.55012E-005	-15.7905
Russians;Italy,Kets	-0.00165444	0.000109986	-15.0423
Russians;Italy,Greenland_Inuits	-0.00130865	8.92408E-005	-14.6643
Russians;Italy,Hezhen	-0.00135985	9.72532E-005	-13.9826
Russians;Italy,Buryats	-0.00122856	8.92671E-005	-13.7627
Russians;Italy,Daur	-0.00132012	9.87876E-005	-13.3632
Russians;Italy,Mongolians	-0.00127065	9.76097E-005	-13.0176
Russians;Italy,Altaians	-0.00101597	7.95118E-005	-12.7775
Russians;Italy,Japanese	-0.00126347	0.000100482	-12.5741
Russians;Italy,Xibo	-0.00119028	9.79618E-005	-12.1505
Russians;Italy,Han	-0.00116083	9.82607E-005	-11.8138
Russians;Italy,Tu	-0.00102453	9.58969E-005	-10.6837
Russians;Aleutian,French	-0.00024049	5.59612E-005	-4.29744
Russians;Italy,Aleutian	-0.00024742	6.42547E-005	-3.85061

Selkup

Selkup;French,Nganasan	-0.00376065	0.000172447	-21.8075
Selkup;Italy,Nganasan	-0.00355303	0.000176013	-20.1861
Selkup;Evenks,French	-0.00354267	0.000180771	-19.5976
Selkup;Evenks,Italy	-0.00323498	0.000183299	-17.6487
Selkup;Russians,Nganasan	-0.00282281	0.000162526	-17.3683
Selkup;Evenks,Russians	-0.00271117	0.000169759	-15.9707
Selkup;Evens_Kamchatka,French	-0.00290262	0.000182971	-15.8639
Selkup;Italy,Evens_Kamchatka	-0.00253667	0.000185811	-13.6519
Selkup;French,Evens	-0.00216477	0.000173614	-12.4688
Selkup;Evens_Kamchatka,Russians	-0.00205904	0.000173744	-11.851
Selkup;Italy,Evens	-0.00181871	0.000177957	-10.2199
Selkup;Russians,Evens	-0.00141089	0.000164499	-8.57689
Selkup;French,Koryaks	-0.00103138	0.000196547	-5.24748
Selkup;Aleutian,Nganasan	-0.000845734	0.000169505	-4.98944
Selkup;Italy,Koryaks	-0.000877175	0.000199008	-4.40774

Selkup;French,Chukchi	-0.000717014	0.000177969	-4.02887
Selkup;French,Oroqen	-0.000753277	0.000188582	-3.99442
<u>Dolgans</u>			
Dolgans;Evenks,French	-0.00305008	0.000131632	-23.1713
Dolgans;Evenks,Italy	-0.00304469	0.000133787	-22.7578
Dolgans;Evenks,Russians	-0.00262167	0.000123828	-21.172
Dolgans;Italy,Nganasan	-0.00264946	0.000130253	-20.3408
Dolgans;French,Nganasan	-0.00255478	0.000131376	-19.4462
Dolgans;Evens_Kamchatka,French	-0.0024279	0.000144206	-16.8364
Dolgans;Russians,Nganasan	-0.00202003	0.000123287	-16.3848
Dolgans;French,Evens	-0.00195231	0.000119217	-16.3761
Dolgans;Italy,Evens_Kamchatka	-0.00236425	0.000145208	-16.2818
Dolgans;Italy,Evens	-0.00190854	0.000120102	-15.891
Dolgans;Evens_Kamchatka,Russians	-0.0019874	0.000135476	-14.6697
Dolgans;Russians,Evens	-0.00160152	0.000113194	-14.1484
Dolgans;Evenks,Aleutian	-0.0016849	0.000125357	-13.4408
Dolgans;San,Nganasan	-0.0018729	0.000139851	-13.3921
Dolgans;Evenks,San	-0.0018564	0.000142929	-12.9883
Dolgans;Aleutian,Nganasan	-0.00137662	0.000121513	-11.329
Dolgans;Evens_Kamchatka,San	-0.00113303	0.000159648	-7.09706
Dolgans;Aleutian,Evens	-0.000705521	0.000107883	-6.5397
Dolgans;San,Evens	-0.000780428	0.000124613	-6.26281
Dolgans;Evens_Kamchatka,Aleutian	-0.000675698	0.000128465	-5.25977
Dolgans;Tu,Nganasan	-0.000324232	9.42151E-005	-3.4414
Dolgans;Han,Nganasan	-0.000282669	0.000089836	-3.1465
<u>*Kets*</u>			
Kets;French,Nganasan	-0.00439225	0.00035552	-12.3545
Kets;Italy,Nganasan	-0.00417373	0.000359764	-11.6013
Kets;Evenks,French	-0.00382046	0.000354984	-10.7623
Kets;Russians,Nganasan	-0.00356796	0.000350667	-10.1748
Kets;Evenks,Italy	-0.00350187	0.000361328	-9.69165
Kets;Evens_Kamchatka,French	-0.00337529	0.000365348	-9.23855
Kets;Evenks,Russians	-0.00310251	0.000349908	-8.86666
Kets;Italy,Evens_Kamchatka	-0.00299842	0.000372451	-8.05052
Kets;French,Evens	-0.00274594	0.000351175	-7.81929
Kets;Evens_Kamchatka,Russians	-0.00264524	0.000360788	-7.33184
Kets;Italy,Evens	-0.00238897	0.000359943	-6.63708
Kets;Russians,Evens	-0.00210561	0.000347658	-6.05656
Kets;Aleutian,Nganasan	-0.00188745	0.000353678	-5.33662
Kets;French,Oroqen	-0.00134175	0.000359999	-3.72709
Kets;French,Koryaks	-0.00137474	0.000381217	-3.60618
Kets;French,Chukchi	-0.00125287	0.000354226	-3.53693
Kets;Evenks,Aleutian	-0.00112862	0.000355409	-3.17557
Kets;Italy,Koryaks	-0.00120963	0.000384575	-3.14536
Yakuts			
Yakuts;French,Evens	-0.00119612	0.000100753	-11.8717
Yakuts;Italy,Evens	-0.00120214	0.000101858	-11.8022
Yakuts;Evenks,Italy	-0.00115999	0.00012594	-9.21072
Yakuts;Evenks,French	-0.0011156	0.000125233	-8.90815
Yakuts;Russians,Evens	-0.000773846	9.56299E-005	-8.09209
Yakuts;Evens_Kamchatka,French	-0.000904377	0.000132075	-6.84744
Yakuts;Italy,Evens_Kamchatka	-0.000890508	0.000133602	-6.66539
Yakuts;Evenks,Russians	-0.000615702	0.000117497	-5.24016

Yakuts;Italy,Nganasan	-0.000570857	0.000130823	-4.36358
Yakuts;French,Nganasan	-0.000426387	0.000130525	-3.2667
Yakuts;Evens_Kamchatka,Russians	-0.000392393	0.000125418	-3.12868

Buryats

Buryats;Italy,Evens_Kamchatka	-0.00237122	0.000142658	-16.6217
Buryats;Evens_Kamchatka,French	-0.00234173	0.000142807	-16.3979
Buryats;Evenks,Italy	-0.00204779	0.0001464	-13.9876
Buryats;Evenks,French	-0.00196002	0.000145853	-13.4384
Buryats;Italy,Evens	-0.00158609	0.000132643	-11.9576
Buryats;French,Evens	-0.0015367	0.000130697	-11.7577
Buryats;Evens_Kamchatka,Russians	-0.00159217	0.000135429	-11.7565
Buryats;French,Oroqen	-0.00148975	0.000131407	-11.3369
Buryats;Italy,Oroqen	-0.00152855	0.00013658	-11.1916
Buryats;French,Hezhen	-0.00123661	0.00013174	-9.38676
Buryats;Russians,Oroqen	-0.00113874	0.000124719	-9.13045
Buryats;Evenks,Russians	-0.00122255	0.000136525	-8.95477
Buryats;Italy,Hezhen	-0.00114466	0.000133976	-8.54378
Buryats;Hezhen,Russians	-0.00101337	0.000124376	-8.14763
Buryats;Evens_Kamchatka,San	-0.00134306	0.000165071	-8.13624
Buryats;Italy,Nganasan	-0.00121194	0.000155517	-7.79296
Buryats;Russians,Evens	-0.000876853	0.000122761	-7.14274
Buryats;Evenks,San	-0.00106255	0.000155984	-6.81189
Buryats;French,Nganasan	-0.0010241	0.000155021	-6.60624
Buryats;French,Japanese	-0.000787614	0.000122381	-6.43577
Buryats;Russians,Japanese	-0.000721728	0.000113294	-6.37039
Buryats;Italy,Japanese	-0.00075664	0.000124798	-6.06294
Buryats;French,Daur	-0.000756392	0.00013184	-5.73722
Buryats;Italy,Daur	-0.000721686	0.000134836	-5.35233
Buryats;Russians,Daur	-0.00063013	0.000121271	-5.19606
Buryats;San,Evens	-0.000661026	0.000135635	-4.87355
Buryats;San,Nganasan	-0.00063843	0.00016282	-3.92108
Buryats;Aleutian,Oroqen	-0.000476701	0.000122018	-3.9068

Xibo

Xibo;Khanty,Han	-0.00169896	0.000084764	-20.0434
Xibo;Han,Evens	-0.00145705	7.57716E-005	-19.2295
Xibo;Han,Dolgans	-0.00141934	7.49014E-005	-18.9495
Xibo;Nenets,Han	-0.00158574	0.000084269	-18.8176
Xibo;Han,Yakuts	-0.00125051	6.95594E-005	-17.9777
Xibo;Evenks,Han	-0.00155614	8.69645E-005	-17.894
Xibo;Selkup,Han	-0.00156104	8.97214E-005	-17.3988
Xibo;Russians,Han	-0.00185484	0.000111079	-16.6984
Xibo;Russians,Japanese	-0.00193665	0.000117743	-16.4481
Xibo;Han,Nganasan	-0.00144072	8.96567E-005	-16.0693
Xibo;French,Japanese	-0.00200741	0.000125738	-15.9651
Xibo;French,Han	-0.00187172	0.000117724	-15.8993
Xibo;Evens_Kamchatka,Han	-0.00142421	8.96329E-005	-15.8894
Xibo;Italy,Japanese	-0.00200984	0.000127654	-15.7444
Xibo;Italy,Han	-0.00182539	0.000119291	-15.3019
Xibo;Chukchi,Han	-0.00126031	8.35679E-005	-15.0812
Xibo;Aleutian,Han	-0.00161492	0.000107295	-15.0513
Xibo;Altaians,Han	-0.00109005	7.24658E-005	-15.0423
Xibo;Tuvans,Han	-0.00104333	6.96101E-005	-14.9883
Xibo;Khanty,Japanese	-0.00132789	9.36488E-005	-14.1795
Xibo;Han,Buryats	-0.000974522	0.000069172	-14.0884

Xibo;Aleutian,Japanese	-0.00155547	0.000114499	-13.585
Xibo;Han,Greenland_Inuits	-0.00120301	9.26877E-005	-12.9791
Xibo;Koryaks,Han	-0.00124389	9.77001E-005	-12.7317
Xibo;Han,Kets	-0.0015135	0.000119566	-12.6582
Xibo;Naukan_Yupik,Han	-0.00116637	9.76308E-005	-11.9467
Xibo;Nenets,Japanese	-0.00107334	9.25015E-005	-11.6035
Xibo;Selkup,Japanese	-0.00116141	0.0001002	-11.5909
Xibo;Altaians,Japanese	-0.000844768	7.82975E-005	-10.7892
Xibo;Japanese,Yakuts	-0.000740174	7.19493E-005	-10.2874
Xibo;Dolgans,Japanese	-0.000832162	8.12279E-005	-10.2448
Xibo;French,Hezhen	-0.00136267	0.000142506	-9.56221
Xibo;Italy,Hezhen	-0.00130413	0.000144027	-9.05477
Xibo;Kets,Japanese	-0.00111759	0.000126029	-8.86768
Xibo;Evenks,Japanese	-0.000803146	0.000091442	-8.78311
Xibo;Tuvans,Japanese	-0.00063773	7.48392E-005	-8.52134
Xibo;Japanese,Evens	-0.000689767	8.12279E-005	-8.49175
Xibo;Hezhen,Russians	-0.00113456	0.000135617	-8.36591
Xibo;Evenks,Tu	-0.000835857	0.000102841	-8.12767
Xibo;Tu,Evens	-0.000733929	9.25367E-005	-7.93122
Xibo;Japanese,Buryats	-0.000569826	7.44769E-005	-7.65104
Xibo;Evens_Kamchatka,Tu	-0.000803958	0.000105211	-7.64137
Xibo;Nganasan,Japanese	-0.000730647	9.69403E-005	-7.53709
Xibo;Oroqen,Han	-0.00058099	7.76102E-005	-7.48601
Xibo;San,Japanese	-0.00106444	0.000143023	-7.44239
Xibo;Han,San	-0.000935571	0.000131026	-7.14035
Xibo;Greenland_Inuits,Japanese	-0.000701189	0.000100581	-6.97141
Xibo;Italy,Oroqen	-0.00102882	0.000155651	-6.60982
Xibo;French,Oroqen	-0.000956626	0.000154053	-6.20971
Xibo;Chukchi,Japanese	-0.000545665	9.02186E-005	-6.04825
Xibo;Tu,Dolgans	-0.000520526	9.00418E-005	-5.78094
Xibo;Evens_Kamchatka,Japanese	-0.000553198	9.65789E-005	-5.72793
Xibo;French,Daur	-0.00084568	0.000149525	-5.65578
Xibo;Italy,Daur	-0.000844374	0.00015191	-5.55839
Xibo;Tu,Nganasan	-0.000583468	0.000107783	-5.41335
Xibo;Aleutian,Hezhen	-0.000712832	0.000132338	-5.38643
Xibo;Hezhen,San	-0.000865185	0.000161298	-5.3639
Xibo;Russians,Daur	-0.000714538	0.000139046	-5.13887
Xibo;Oroqen,San	-0.000847469	0.000165517	-5.12013
Xibo;Tu,Yakuts	-0.000416279	8.42456E-005	-4.94125
Xibo;Tu,Oroqen	-0.000439918	9.03839E-005	-4.86722
Xibo;Tu,Chukchi	-0.000500047	0.000105848	-4.72419
Xibo;Naukan_Yupik,Japanese	-0.00047241	0.000103037	-4.58488
Xibo;Koryaks,Japanese	-0.000458172	0.000103911	-4.40926
Xibo;Daur,San	-0.000639175	0.000153804	-4.15577
Xibo;Russians,Oroqen	-0.000600736	0.000147214	-4.08069
Xibo;Aleutian,Daur	-0.00049774	0.000130354	-3.81836
Xibo;Tu,Koryaks	-0.000456025	0.000123324	-3.69778
Xibo;Han,Daur	-0.000248151	7.42987E-005	-3.3399
Xibo;Oroqen,Japanese	-0.000274193	8.24512E-005	-3.32552
Xibo;Tu,Buryats	-0.000270001	8.15191E-005	-3.31212

Hezhen

Hezhen;Evens_Kamchatka,Han	-0.0014781	9.81642E-005	-15.0574
Hezhen;Evenks,Han	-0.0013163	9.48334E-005	-13.8802
Hezhen;Han,Evens	-0.00103743	8.64267E-005	-12.0036
Hezhen;Han,Nganasan	-0.00111614	0.000101505	-10.9959

Hezhen;Evens_Kamchatka,Japanese	-0.000812419	0.000103958	-7.81491
Hezhen;Evenks,Japanese	-0.000768643	9.99446E-005	-7.69069
Hezhen;Evens_Kamchatka,Tu	-0.000810732	0.000113253	-7.15857
Hezhen;Koryaks,Han	-0.000648662	0.000110339	-5.87882
Hezhen;Nganasan,Japanese	-0.000611404	0.000106502	-5.74079
Hezhen;Nenets,Han	-0.000530905	0.000100068	-5.30543
Hezhen;Japanese,Evens	-0.000475484	9.16484E-005	-5.18814
Hezhen;Han,Dolgans	-0.000468022	9.16898E-005	-5.10441
Hezhen;Evenks,Tu	-0.000548907	0.000110126	-4.98434
Hezhen;Chukchi,Han	-0.00050388	0.00010127	-4.97563

Oroqen

Oroqen;Han,Nganasan	-0.00174975	9.60477E-005	-18.2175
Oroqen;Evenks,Han	-0.00164901	0.000092895	-17.7514
Oroqen;Evens_Kamchatka,Han	-0.00147007	0.00010276	-14.3059
Oroqen;Nganasan,Japanese	-0.00134647	9.77793E-005	-13.7705
Oroqen;Evenks,Japanese	-0.00120281	9.31593E-005	-12.9113
Oroqen;Han,Evens	-0.00102297	8.82952E-005	-11.5858
Oroqen;Evenks,Tu	-0.0010698	0.000102456	-10.4416
Oroqen;Tu,Nganasan	-0.00103357	0.000107226	-9.63915
Oroqen;Evens_Kamchatka,Tu	-0.000990893	0.00010936	-9.06083
Oroqen;Evens_Kamchatka,Japanese	-0.000905858	0.000103021	-8.79293
Oroqen;Xibo,Nganasan	-0.000890019	0.000110581	-8.04856
Oroqen;Daur,Nganasan	-0.000719853	0.000108349	-6.64384
Oroqen;Evenks,Xibo	-0.000673859	0.000102598	-6.56795
Oroqen;Japanese,Evens	-0.000562487	8.85823E-005	-6.34988
Oroqen;Evens_Kamchatka,Xibo	-0.000626854	0.000107648	-5.82321
Oroqen;Evenks,Daur	-0.00054652	0.000105468	-5.18187
Oroqen;Tu,Evens	-0.000440924	9.59997E-005	-4.59297
Oroqen;Evenks,Mongolians	-0.000448256	0.000101042	-4.43634
Oroqen;Hezhen,Nganasan	-0.000473594	0.000108859	-4.35052
Oroqen;Mongolians,Nganasan	-0.000398827	0.000104955	-3.79998
Oroqen;Evens_Kamchatka,Mongolians	-0.000368281	0.000106648	-3.45323
Oroqen;Han,Dolgans	-0.000298909	8.80698E-005	-3.394

Tu

Tu;Russians,Han	-0.00189832	0.000102046	-18.6026
Tu;French,Han	-0.00201658	0.000108586	-18.5712
Tu;Italy,Han	-0.00203462	0.00011173	-18.2101
Tu;Khanty,Han	-0.00125407	8.03701E-005	-15.6037
Tu;Aleutian,Han	-0.00151238	0.000103752	-14.5769
Tu;Italy,Japanese	-0.00173125	0.00012273	-14.1061
Tu;French,Japanese	-0.00166445	0.000119785	-13.8953
Tu;Russians,Japanese	-0.00149231	0.000112197	-13.3008
Tu;Han,San	-0.00150348	0.000120411	-12.4863
Tu;Selkup,Han	-0.00111659	8.97997E-005	-12.4342
Tu;Nenets,Han	-0.00100333	8.46572E-005	-11.8517
Tu;Han,Kets	-0.00119987	0.000115521	-10.3866
Tu;Altaians,Han	-0.000755601	7.28338E-005	-10.3743
Tu;Han,Greenland_Inuits	-0.000840328	9.40677E-005	-8.93322
Tu;Aleutian,Japanese	-0.000965106	0.000114601	-8.42147
Tu;Han,Dolgans	-0.000624166	0.000074196	-8.4124
Tu;San,Japanese	-0.00114453	0.000139271	-8.21795
Tu;Tuvans,Han	-0.000566121	0.000071022	-7.97106
Tu;Han,Yakuts	-0.000559589	7.26014E-005	-7.70768
Tu;Han,Nganasan	-0.000582603	9.22247E-005	-6.31721

Tu;Han,Buryats	-0.000429874	6.99872E-005	-6.14218
Tu;Naukan_Yupik,Han	-0.000566068	0.00010032	-5.64264
Tu;Han,Evens	-0.000448471	8.01795E-005	-5.59333
Tu;Chukchi,Han	-0.000485613	8.72877E-005	-5.56336
Tu;French,Hezhen	-0.000786863	0.000144312	-5.4525
Tu;Koryaks,Han	-0.00051322	9.62678E-005	-5.33117
Tu;Italy,Hezhen	-0.000792693	0.000149666	-5.29641
Tu;Evenks,Han	-0.000445639	8.84945E-005	-5.03578
Tu;Hezhen,San	-0.000712432	0.000152988	-4.6568
Tu;Khanty,Japanese	-0.000395183	9.51868E-005	-4.15165
Tu;Evens_Kamchatka,Han	-0.000345606	8.97518E-005	-3.85069
Tu;Hezhen,Russians	-0.000457376	0.000138296	-3.30722
Tu;Daur,San	-0.000492759	0.000153318	-3.21396

Daur

Daur;Han,Evens	-0.00122169	8.26582E-005	-14.7801
Daur;Evens_Kamchatka,Han	-0.00139477	9.72095E-005	-14.3481
Daur;Evenks,Han	-0.00133068	9.39265E-005	-14.1672
Daur;Han,Nganasan	-0.00125808	9.57517E-005	-13.139
Daur;Han,Dolgans	-0.000833024	8.39423E-005	-9.92378
Daur;Nenets,Han	-0.000823566	9.31407E-005	-8.84216
Daur;Evenks,Japanese	-0.000816536	9.52008E-005	-8.57699
Daur;Japanese,Evens	-0.000693271	0.000083837	-8.26927
Daur;Nganasan,Japanese	-0.000786865	9.91835E-005	-7.93343
Daur;Evens_Kamchatka,Japanese	-0.000762617	9.64379E-005	-7.90786
Daur;Han,Yakuts	-0.000610182	7.78407E-005	-7.83886
Daur;Evens_Kamchatka,Tu	-0.000827216	0.000108728	-7.60813
Daur;Chukchi,Han	-0.000619889	9.45654E-005	-6.55514
Daur;Evenks,Tu	-0.000663086	0.000105916	-6.26048
Daur;Koryaks,Han	-0.000652599	0.000108797	-5.99834
Daur;Tu,Evens	-0.000551272	9.42695E-005	-5.84783
Daur;Nenets,Japanese	-0.000550029	9.49755E-005	-5.79128
Daur;Dolgans,Japanese	-0.000484704	8.63683E-005	-5.61207
Daur;Han,Kets	-0.000753124	0.000135168	-5.57175
Daur;Selkup,Han	-0.00055447	0.00010015	-5.53641
Daur;Khanty,Han	-0.000483033	9.44892E-005	-5.11204
Daur;Naukan_Yupik,Han	-0.000483638	0.000108139	-4.47237
Daur;Kets,Japanese	-0.000596073	0.000135871	-4.38705
Daur;Japanese,Yakuts	-0.000338699	7.88999E-005	-4.29276
Daur;Tu,Nganasan	-0.000453525	0.000111575	-4.06477
Daur;Selkup,Japanese	-0.000393692	0.000104057	-3.78342
Daur;Han,Buryats	-0.000288074	7.80766E-005	-3.68963
Daur;Khanty,Japanese	-0.000350826	9.58862E-005	-3.65878
Daur;Tuvans,Han	-0.000263942	8.27072E-005	-3.19129

French

French;Italy,Russians	-0.000222021	3.55334E-005	-6.24824
French;Italy,Selkup	-0.000296423	6.48196E-005	-4.57305
French;Italy,Aleutian	-0.000228951	5.35804E-005	-4.27303
French;Italy,Khanty	-0.000219616	5.98821E-005	-3.66747
French;Italy,Kets	-0.000307332	9.20913E-005	-3.33726

Greenland_Inuits

Greenland_Inuits;Italy,Naukan_Yupik	-0.00170939	0.000192151	-8.89606
Greenland_Inuits;Naukan_Yupik,French	-0.00168411	0.000193324	-8.71133
Greenland_Inuits;Naukan_Yupik,Russians	-0.000950008	0.00018426	-5.15579

Chukchi

Chukchi;Naukan_Yupik,Koryaks	-0.0009769	0.000118556	-8.24002
Chukchi;Koryaks,Greenland_Inuits	-0.000975519	0.000132622	-7.35561

Table S3. **Results of the AHG test**

	A/B,C	B/C,A	A/C,B
Tungusic speakers:			
Evenks_StonyTunguska	0.0143	-0.0168	-0.0026
Evenks_Taimyr	0.01	-0.0091	0.0007
Evens_Sakkyryyr	-0.1127	0.1285	0.0159
Evens_Tompo	-0.044	0.0494	0.0054
Evens_Sebjan	-0.1473	0.1676	0.0203
Eastern Evens (Evens_Kamchatka and Evens Berezovka)	-0.0016	-0.0072	-0.0088
Oroqen	0.001	0.2375	0.2386
Hezhen	-0.0864	0.3882	0.3018
	A/B,C	B/C,A	A/C,B
Turkic speakers:			
Yakuts_CEPH	-0.002	0.0076	0.0055
Yakuts_Verhoyansk	0.0026	-0.0111	-0.0084
Dolgans_Anabar	-0.0304	0.0054	-0.025
Dolgans_Taimyr	-0.0302	0.0077	-0.0224

	A/B,C	B/C,A	A/C,B									
Samoyedic speakers												
Selkup and Nenets_Yamal	-0.0065	-0.1461	-0.1526									
	A/B,C	B/C,A	A/C,B	A/B,D	B/D,A	A/D,B	A/D,C	D/C,A	A/C,D	B/C,D	D/C,B	D/B,C
South Siberia												
Buryats	0.0008	0.0019	0.0027									
Tuvans	0.0009	0.006	0.007	0.0026	-0.00221	0.0004	0.008	0.0082	0.0162	0.0136	-0.0065	0.0071
Altaians	0.0042	-0.007	-0.0027	-0.0161	0.0037	-0.0125	-0.0131	-0.0106	0.0025	0.0186	-0.0098	0.0088

* Eastern Evens are pooled because of small sample sizes

To work out the order of admixture events in populations with more than three sources of ancestry, we consider each trio of ancestries separately. For example, for the Altaians we first try to resolve the ordering of A,B and C ancestries. However the results of the three covariance tests for all possible configurations of A,B and C are inconclusive, hence this ordering cannot be resolved. The next trio we consider is A,B and D. Here, the smallest covariance is observed between the ratio of B/D and A, we can therefore conclude that A is the most recent of these three ancestries. The next trio is A,D and C. For this trio the smallest absolute value of covariance is observed between A/C and D, hence A ancestry must have mixed with C ancestry before mixing with D ancestry. The covariance tests for the last trio B,C and D are again inconclusive, yet the only possible configuration for the entire admixture graph given these results is (BD)(AC).

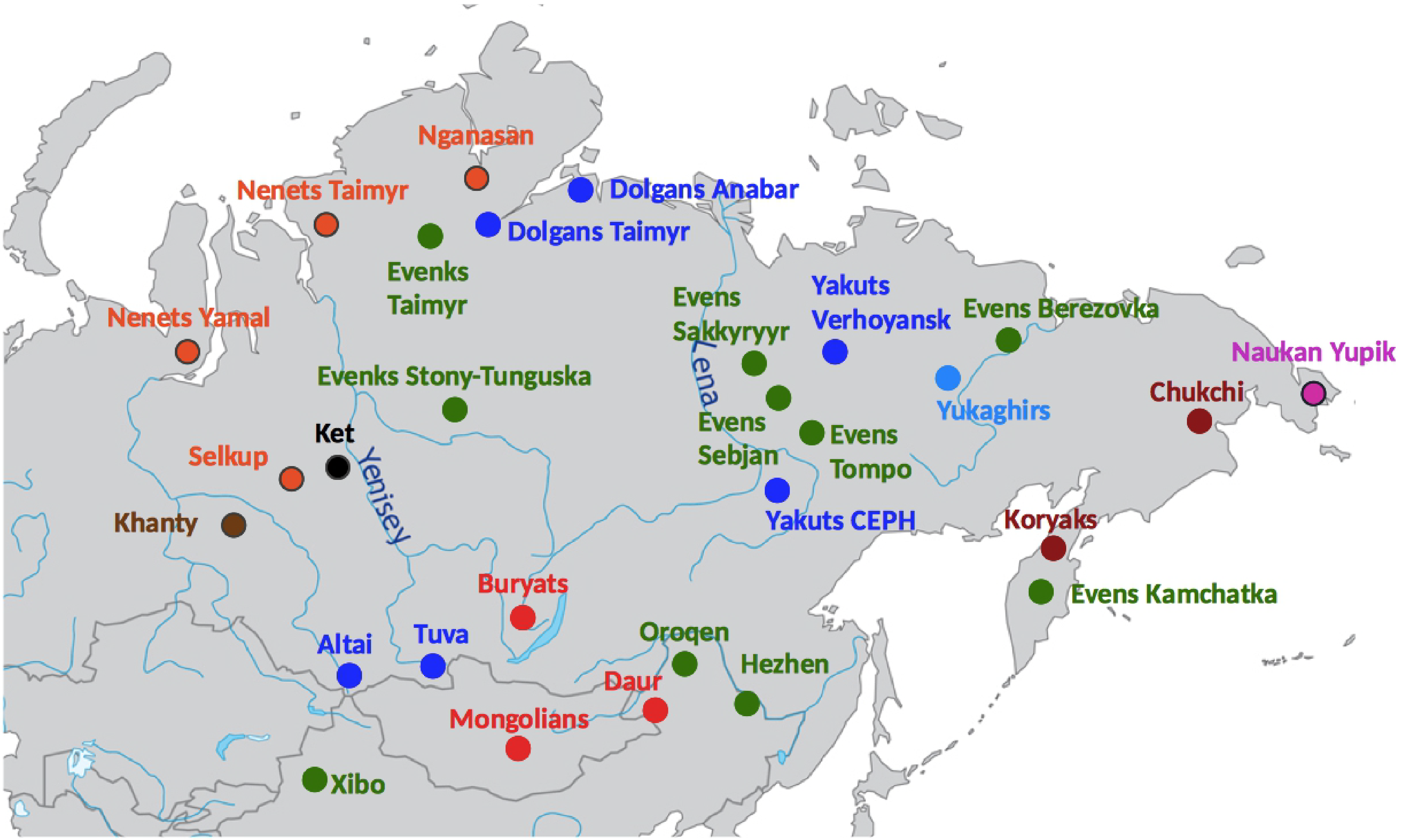

FIGURE S1. Sampling locations for the Siberian populations included in the analyses. The names of the populations are coloured according to their linguistic affiliation as follows: red = Mongolic, blue = Turkic, green = Tungusic, brown = Ugric, orange = Samoyedic, black = Yenisseic, azure = Yukaghirs, maroon = Chukotko-Kamchatkan, pink = Aleut-Yupik-Inuit.

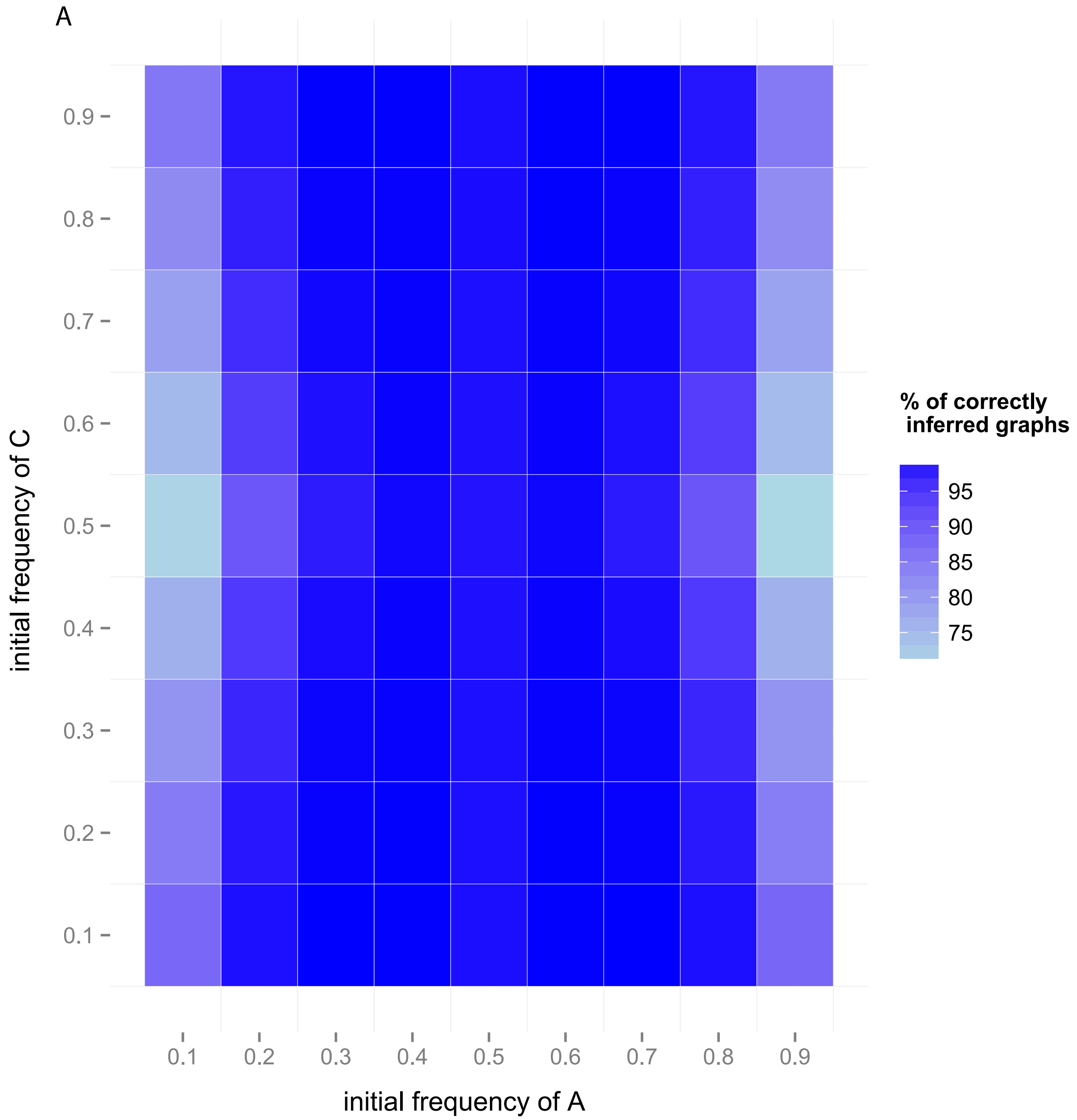

A
0.9
0.8
0.7
0.6
0.5
0.4
0.3
0.2
0.1
initial frequency of C
0.1
0.2
0.3
0.4
0.5
0.6
0.7
0.8
0.9
initial frequency of A
% of correctly inferred graphs
95
90
85
80
75

B

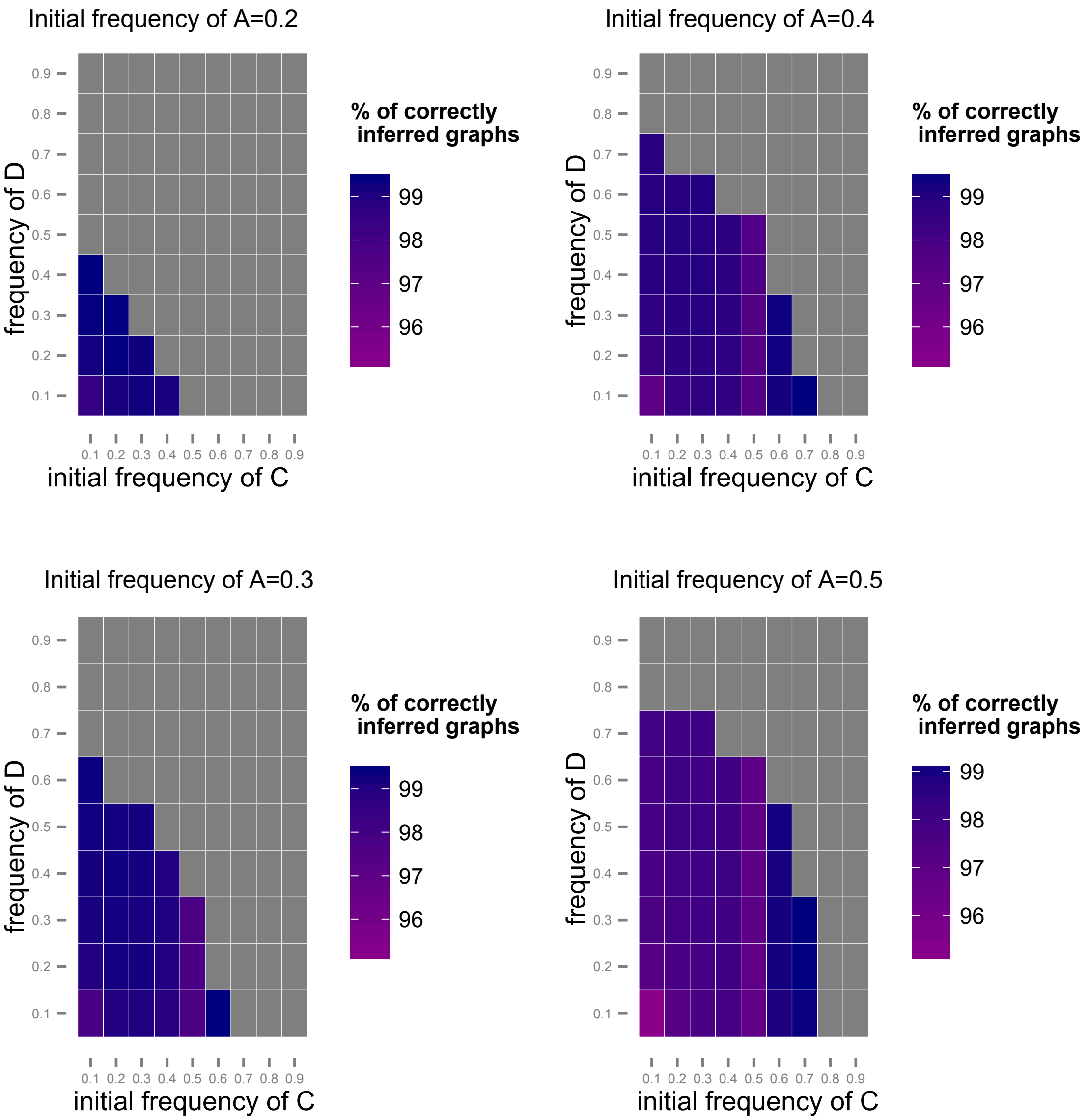

Initial frequency of A=0.2
Initial frequency of A=0.4
Initial frequency of A=0.3
Initial frequency of A=0.5
frequency of D
initial frequency of C
% of correctly inferred graphs
99
98
97
96
0.1
0.2
0.3
0.4
0.5
0.6
0.7
0.8
0.9

C

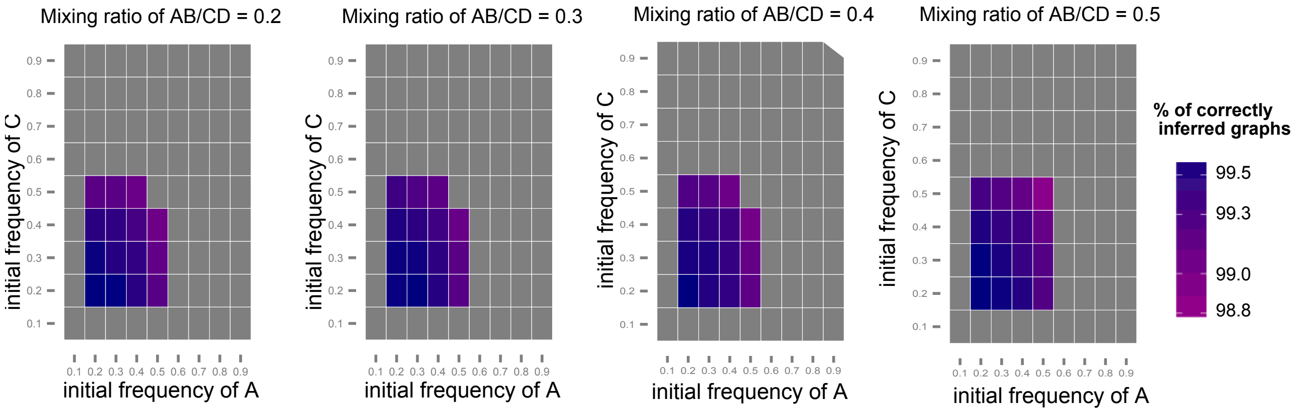

FIGURE S2. Results of simulations to validate the Admixture History Graph method. (A) The simulated history is ((AB)C), where ancestries A and B were already present in a population at the time of admixture with C. Each rectangle summarizes the results for 100,000 simulations performed for a given set of A and C values, and the colour intensity reflects the number of simulations in which the tree was inferred correctly. The lowest value of 70% correctly inferred graphs is observed when the final frequency of A (or B) is below 10%, and the frequency of C is around 50%. (B) The simulated history is (((AB)C)D), where an already admixed population with two ancestry components A and B first admixes with C, which is then followed by another admixture event with D. Based on the simulation results of ((AB)C) admixture histories (previous panel), the scenarios where the final frequency of A (or B) dropped to below 10% were excluded. In each panel, each rectangle summarizes the results for 100,000 simulations performed for a given set of C and D values, with the value of A given in the panel's title. The color intensity reflects the number of simulations in which the tree was inferred correctly. Grey rectangles denote impossible combinations of A, C and D. For example, if the initial frequency of A is 0.2, that of C 0.5, and that of D 0.2, then addition of C would reduce the frequency of A to 0.1, while subsequent influx of ancestry D would bring its final frequency to 0.08 (see formulae given in Figure 2), which is too low given the criteria we set for these simulations. (C) The simulated history is ((AB)(CD)), where admixture occurs between two already admixed population. In each panel, each rectangle summarizes the results for 100,000 simulations performed for a given set of A and C values, with the value given in the panel's title denoting the mixing ratio between the AB and CD populations. The color intensity reflects the number of simulations in which the tree was inferred correctly, and grey rectangles denote impossible combinations of A, C and D.

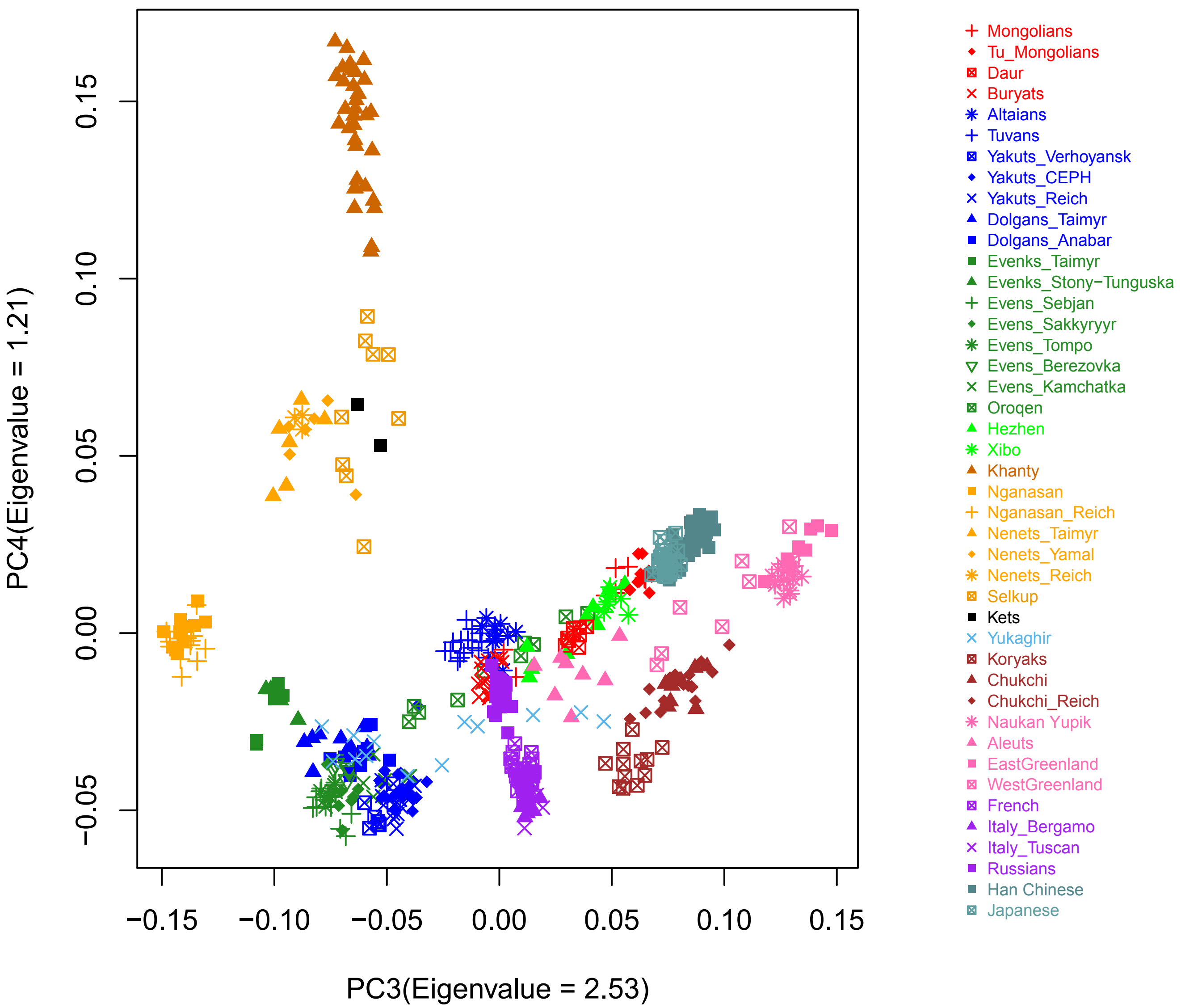

FIGURE S3. Results of the PC analysis: PC3 vs PC4. Each coloured label represents an individual, and individuals are coloured according to their linguistic affiliation, see legend to Figure 1 of the main text for details.

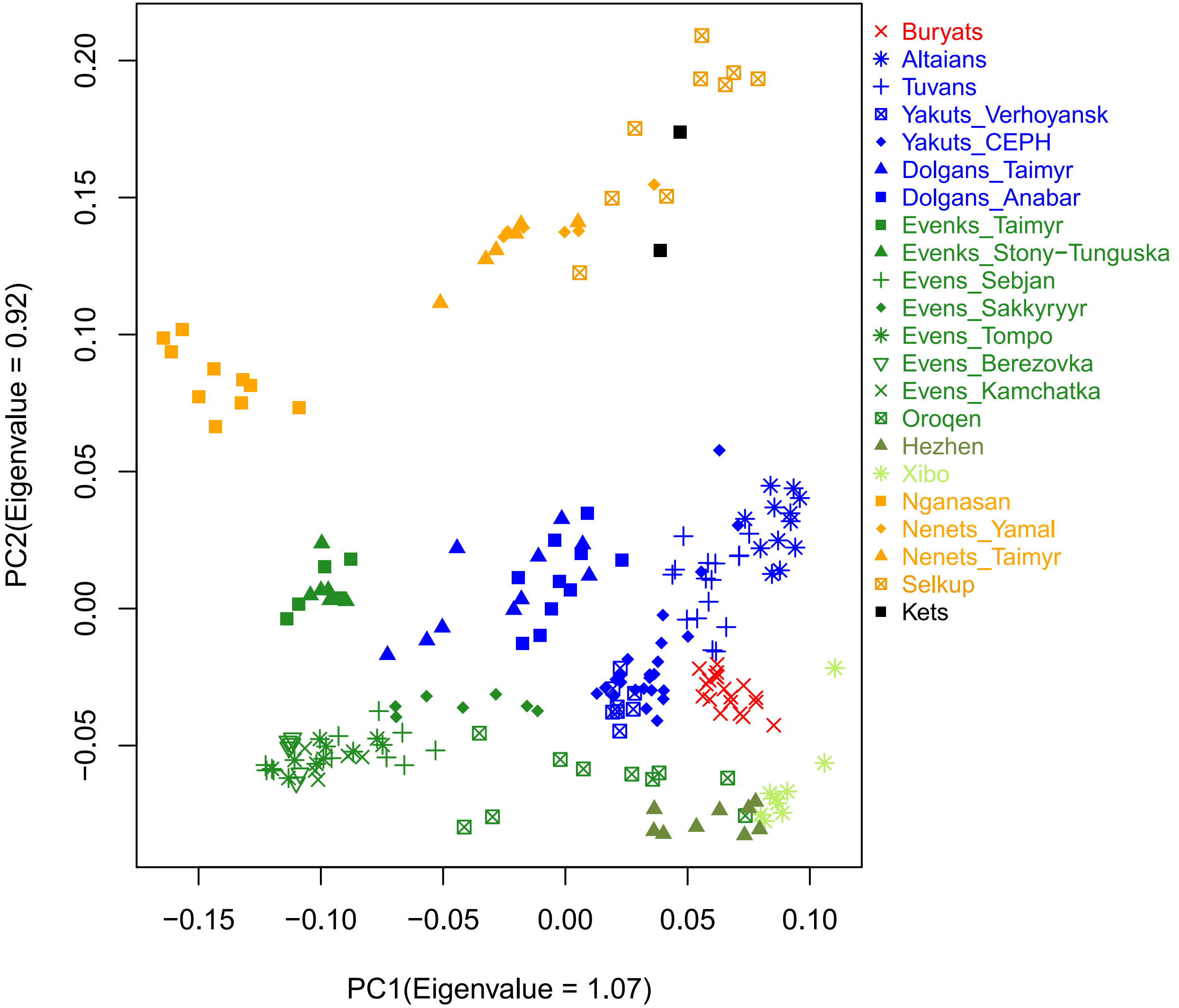

FIGURE S4. Results of the PC analysis for a subset of the dataset. To better understand the relationships between closely related Siberian populations, the PC analysis was performed excluding those populations driving the main axes of variation in the PCA for the entire dataset (all non-Siberian populations as well as the populations of Chukotka and Kamchatka , Khanty and Kets). The heavily admixed Yukaghirs were also excluded from this analysis.

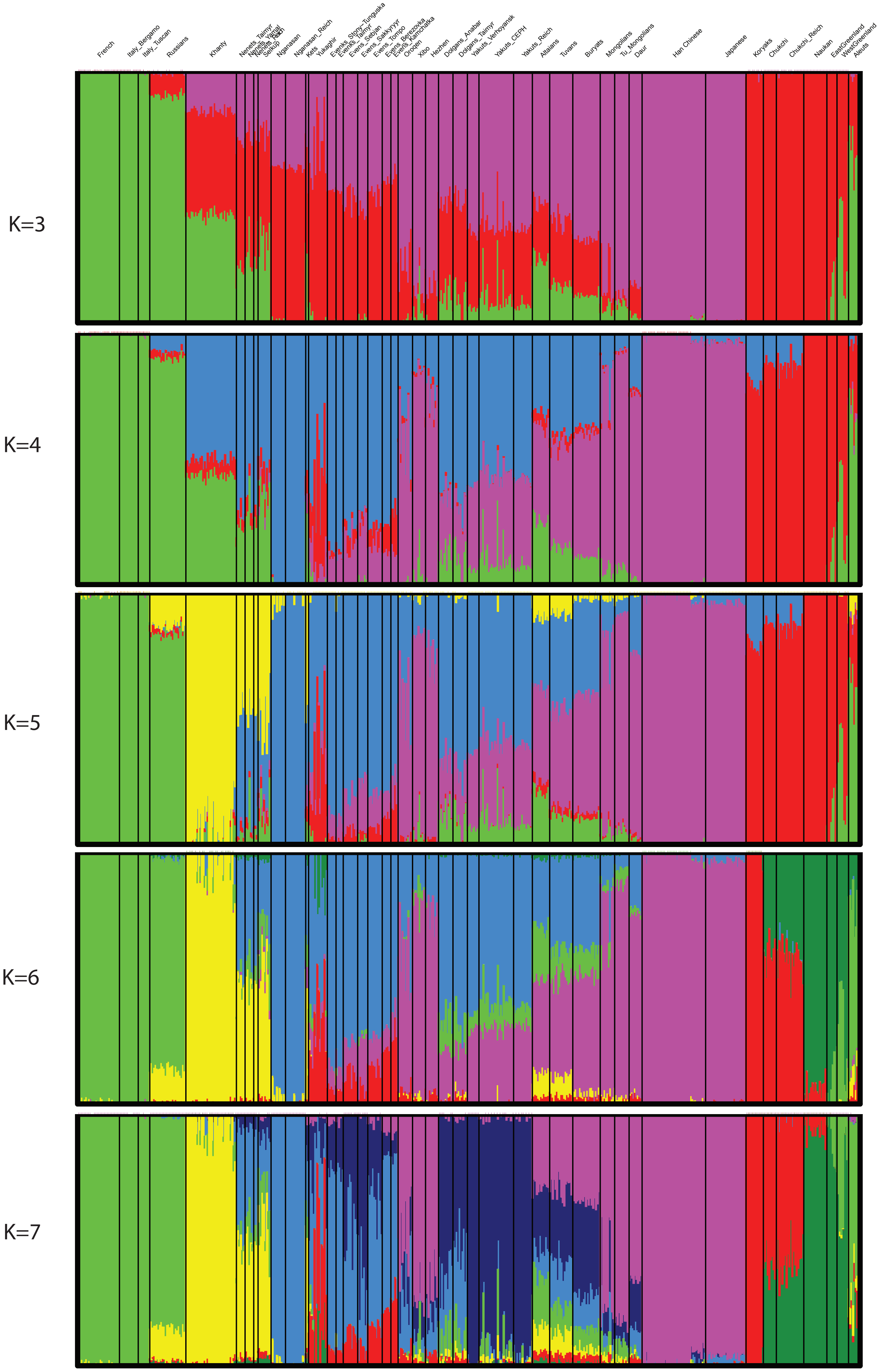

FIGURE S5. Results of the ADMIXTURE analysis for K = 3 through K = 7.

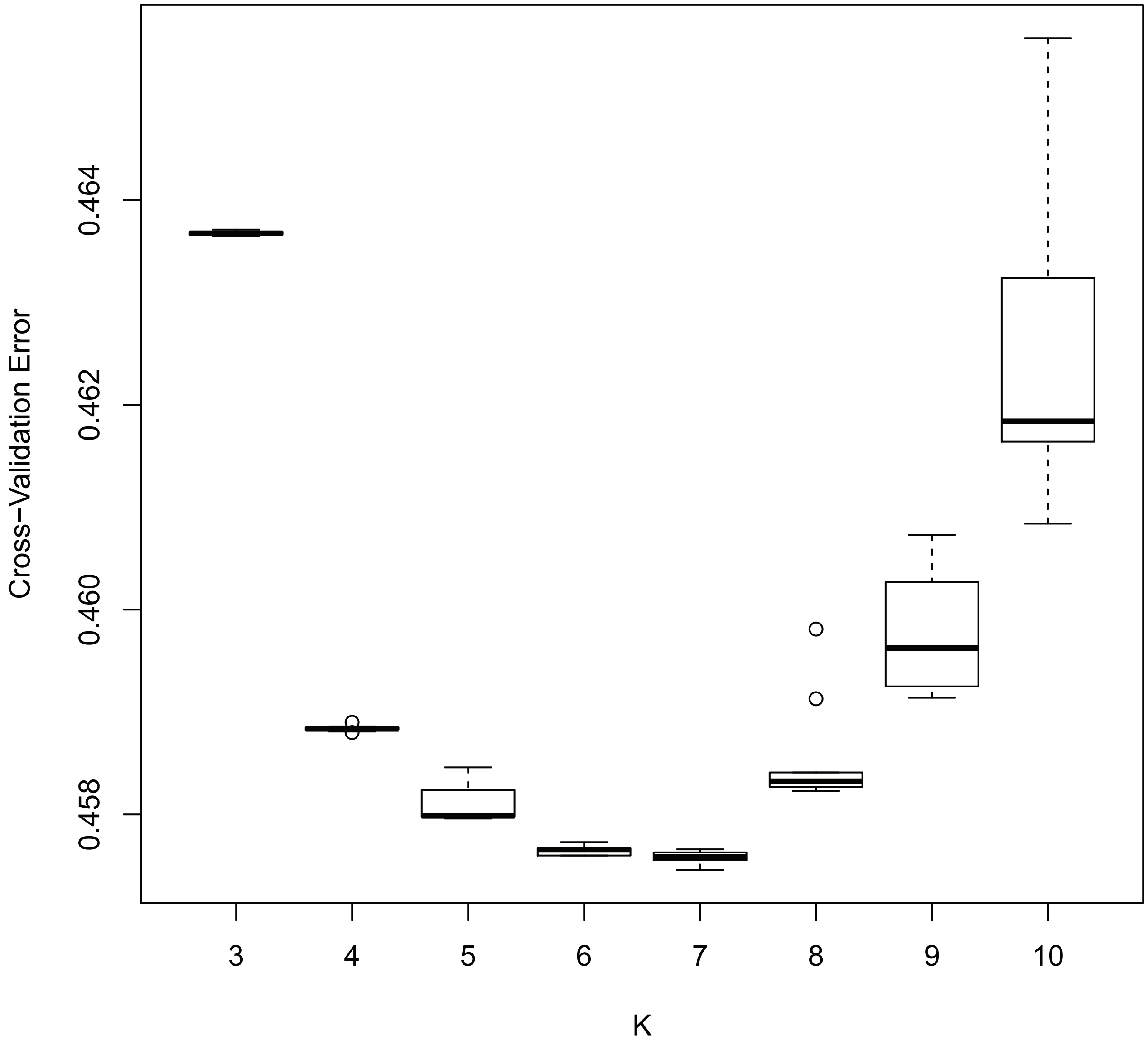

FIGURE S6. Estimated cross-validation error for the ADMIXTURE runs for K=3 through K=10.

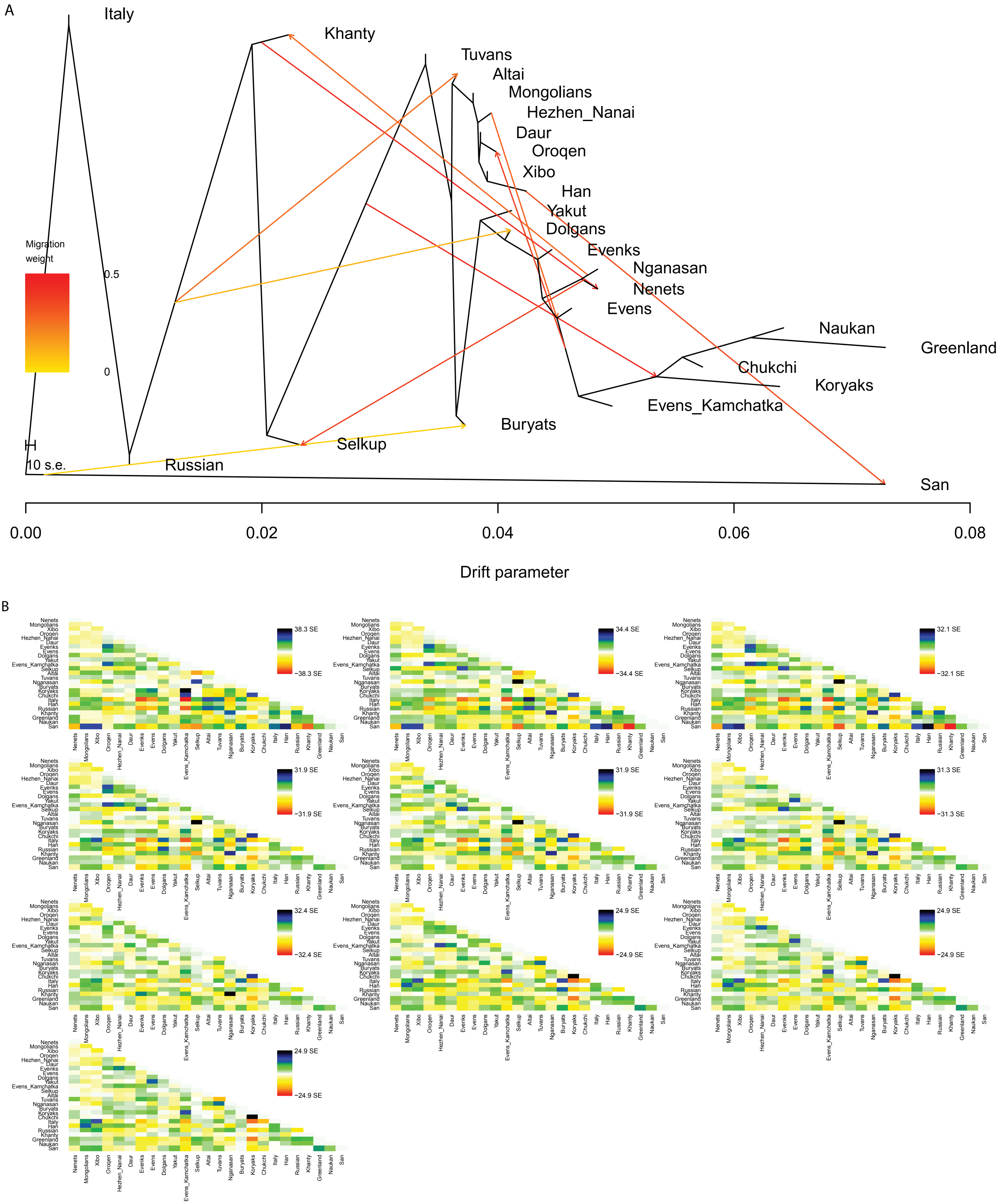

FIGURE S7. TreeMix analysis (A) TreeMix inferred maximum-likelihood tree with ten migration edges. (B) Residual fit from maximum likelihood trees with 1 to 10 migration edges. Positive residuals indicate pairs of populations where the model underestimates the observed covariance, and thus a migration edge between these populations might improve the fit. Negative residuals indicate pairs of populations where the observed covariance is overestimated, suggesting that these populations are being forced too close together due to unmodeled migration somewhere in the tree.

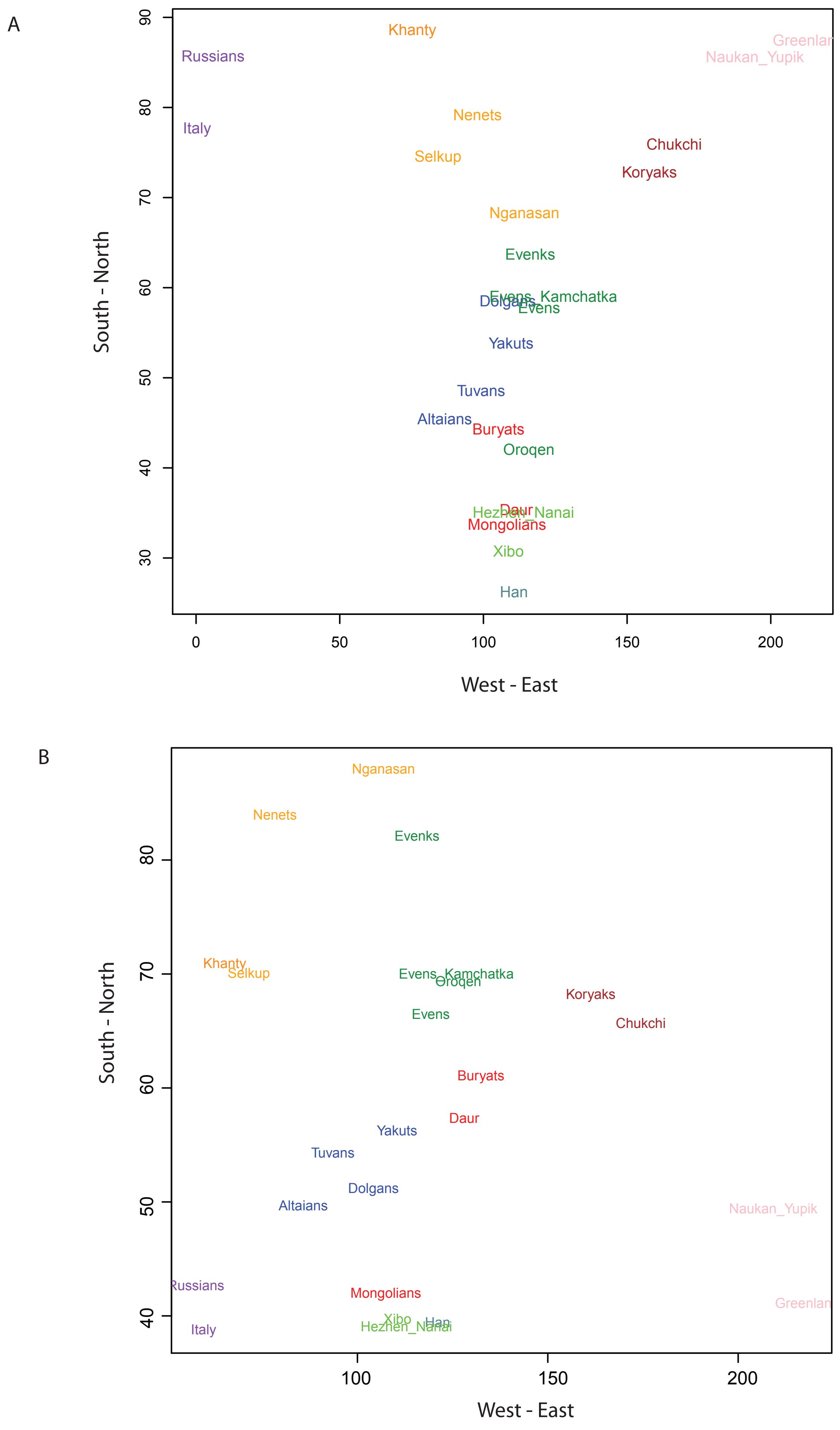

FIGURE S8. SpaceMix analysis (A) A geogenetic map inferred with SpaceMix under a model "Target", which allows populations to find their own location (i.e. migrate), but does not allow admixture. (B) A geogenetic map inferred with SpaceMix under a model "Source and Target", which allows populations to find their own location and to draw admixture (i.e. a model which allows both migration and admixture).

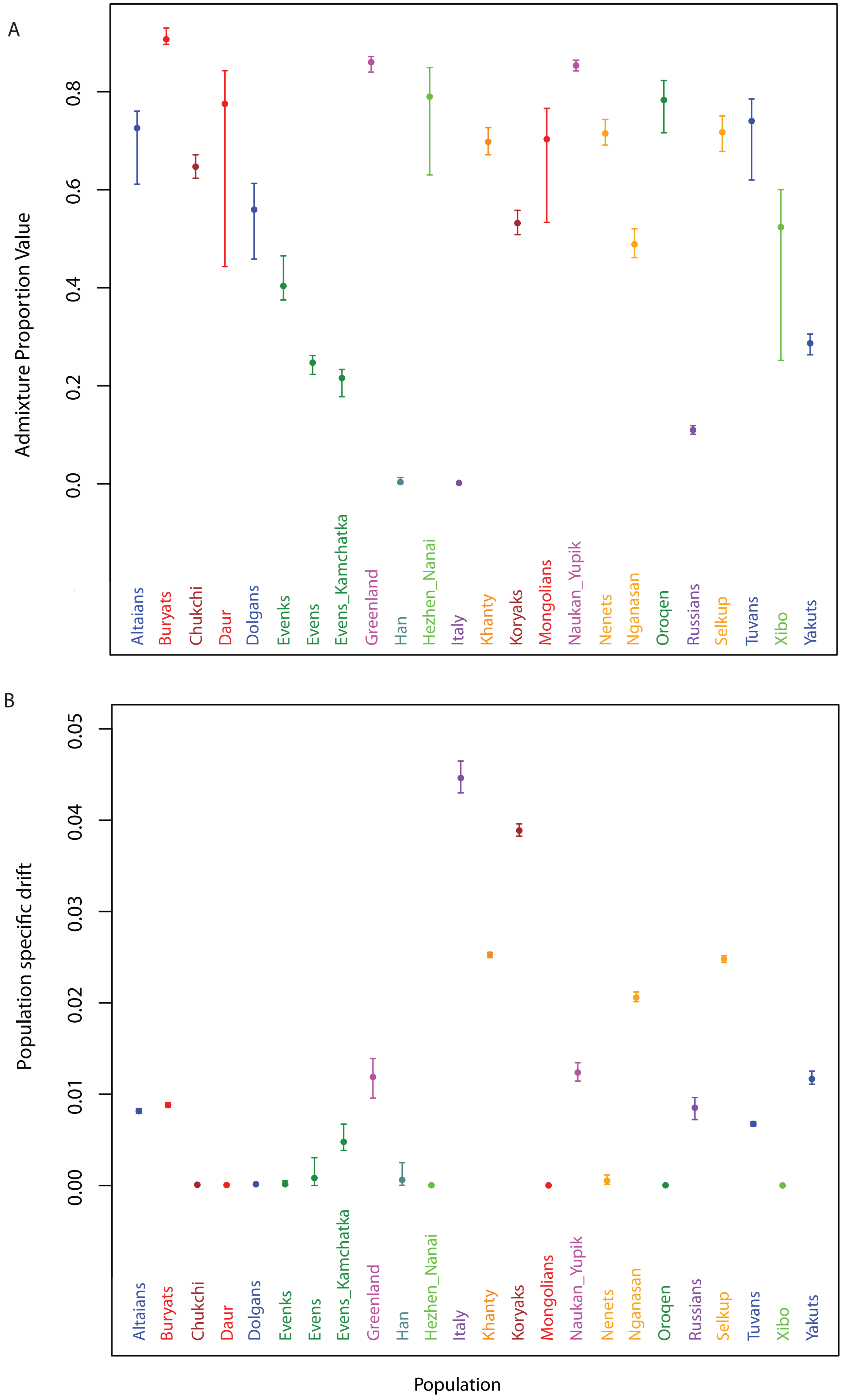

FIGURE S9. Admixture and drift estimates from SpaceMix (A) Mean admixture proportions (and 95% CIs) for each population inferred by SpaceMix. (B) Population specific drift parameter (and 95% CIs) inferred by SpaceMix.

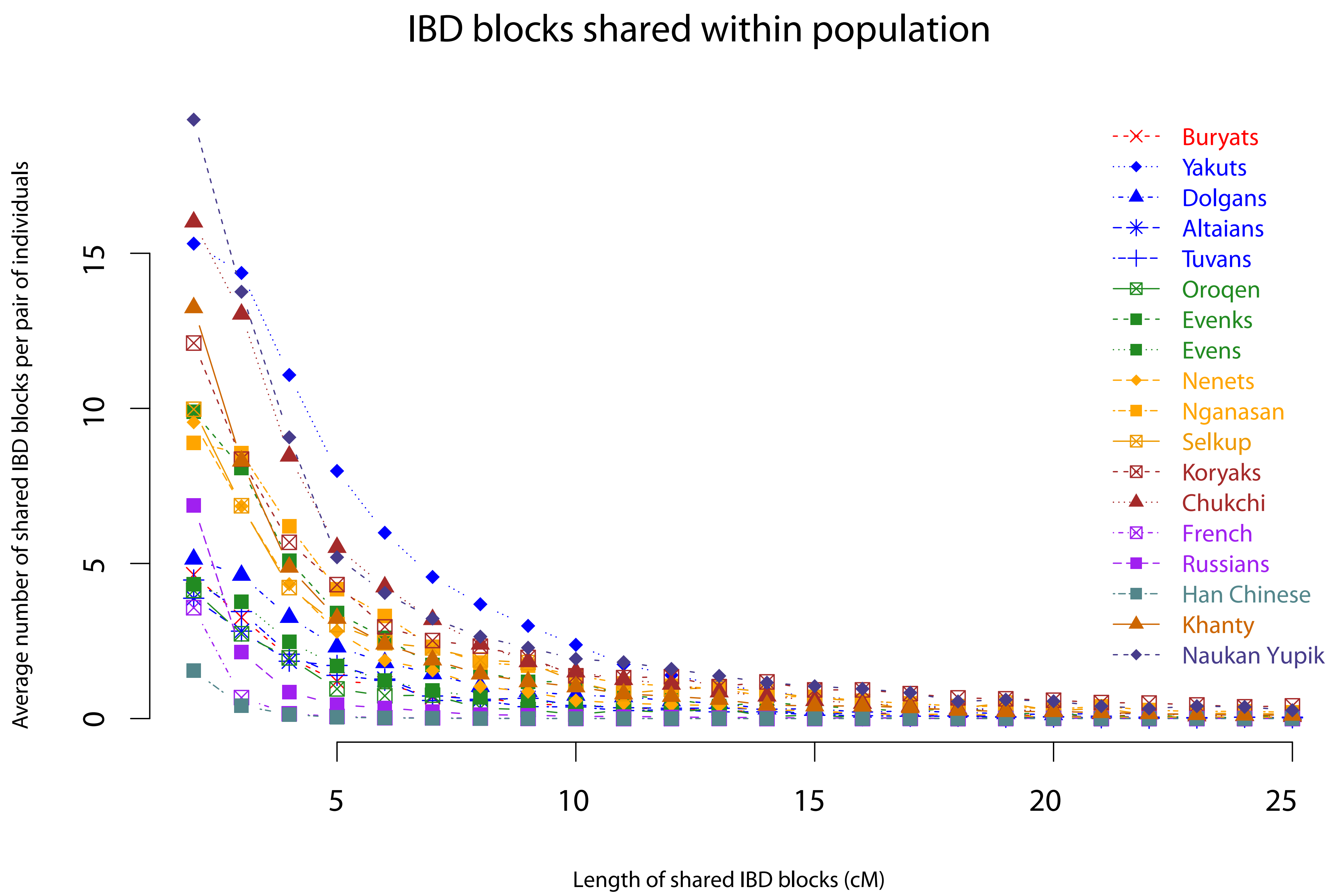

FIGURE S10. Analysis of IBD blocks shared within populations. The average number of IBD blocks shared per pair of individuals belonging to the same population is plotted against the genetic length of such blocks (blocks smaller than 2 cM were excluded from the analysis). Populations are coloured according to their linguistic affiliation, and the colouring scheme is described in the legend to Figure 1 of the main text.

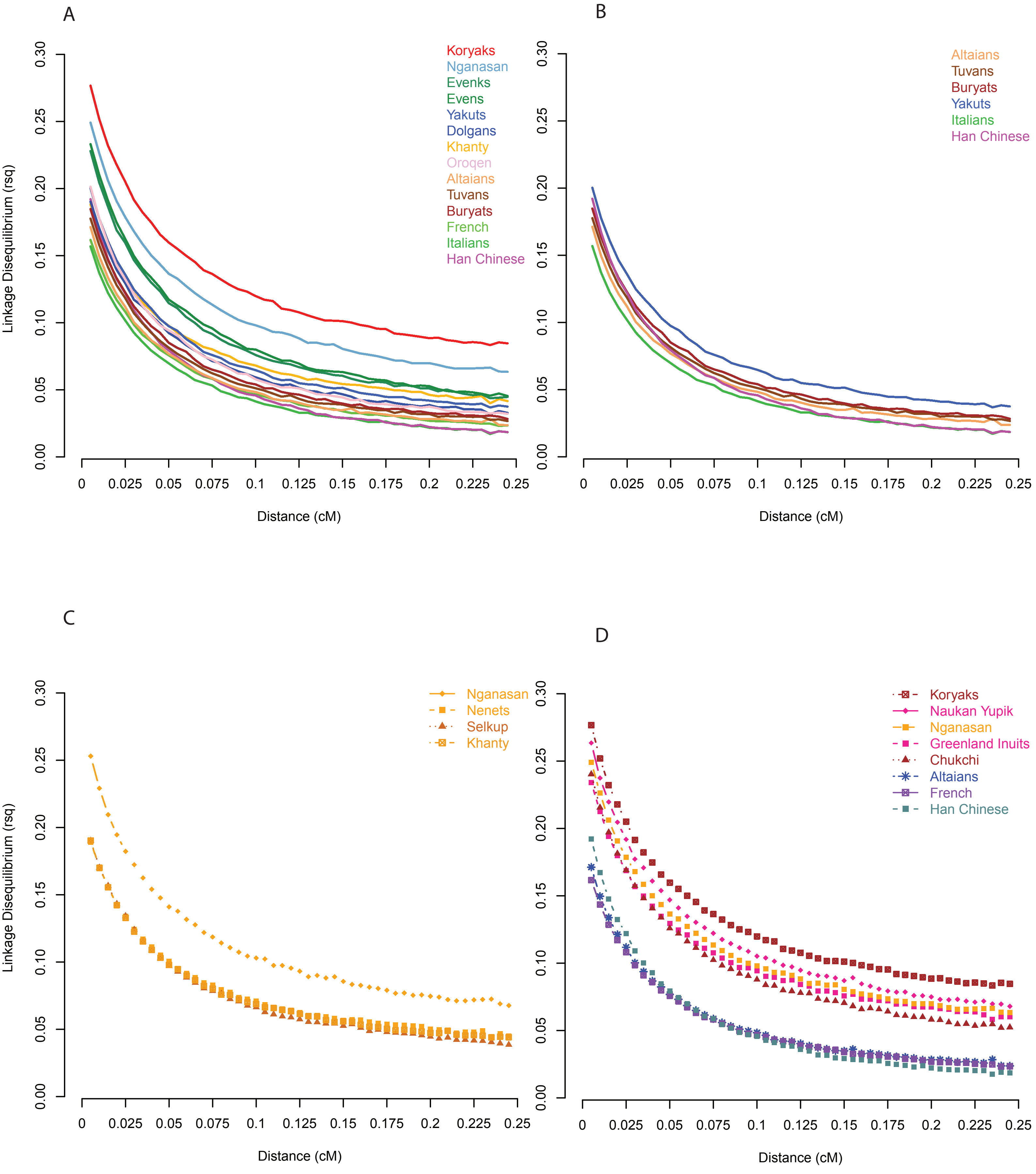

FIGURE S11. LD patterns in populations of Siberia. Linkage disequilibrium (LD) is measured for each population and each pair of SNPs within 50 evenly spaced recombination distance categories. The shortest genetic distances between the SNPs are represented on the left and the largest genetic distances on the right. (A) LD patterns in a representative subset of populations. (B) LD pattern in the Yakuts in comparison to Europeans, East Asians and populations of South Siberia. (C) LD pattern in the Nganasan in comparison to the other Samoyedic-speakers and the Ugric-speaking Khanty. (D) LD pattern in the populations that live on the margins of the Siberian landmass in comparison to Europeans, East Asians and Altaians of South Siberia.

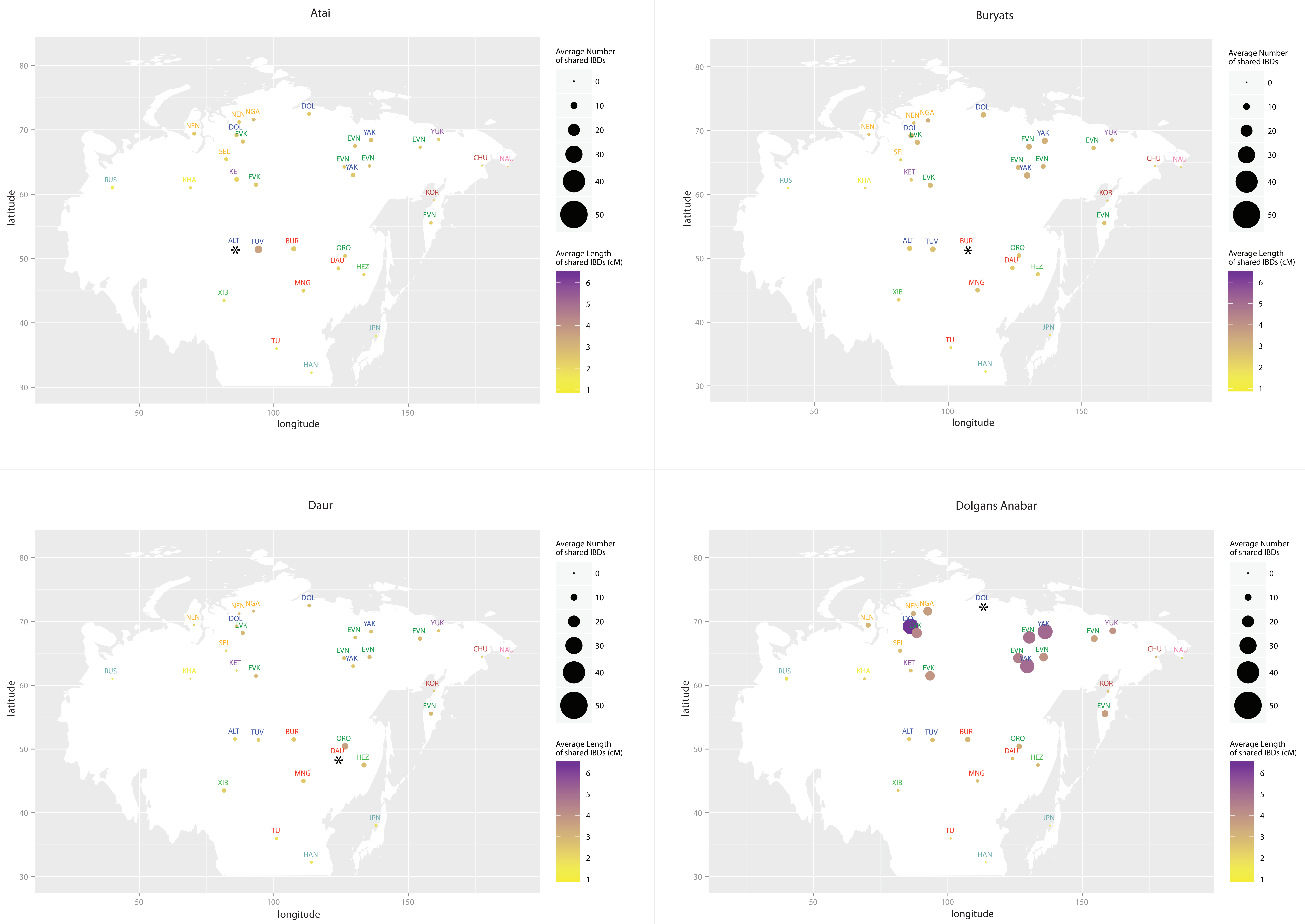
Atai
Buryats
Daur
Dolgans Anabar
latitude
longitude
80
70
60
50
40
30
50
100
150
NEN
NGA
DOL
EVK
SEL
KET
RUS
KHA
EVN
YAK
YUK
CHU
NAU
KOR
ALT
TUV
BUR
ORO
DAU
HEZ
MNG
XIB
TU
JPN
HAN
Average Number
of shared IBDs
0
10
20
30
40
50
Average Length
of shared IBDs (cM)
6
5
4
3
2
1

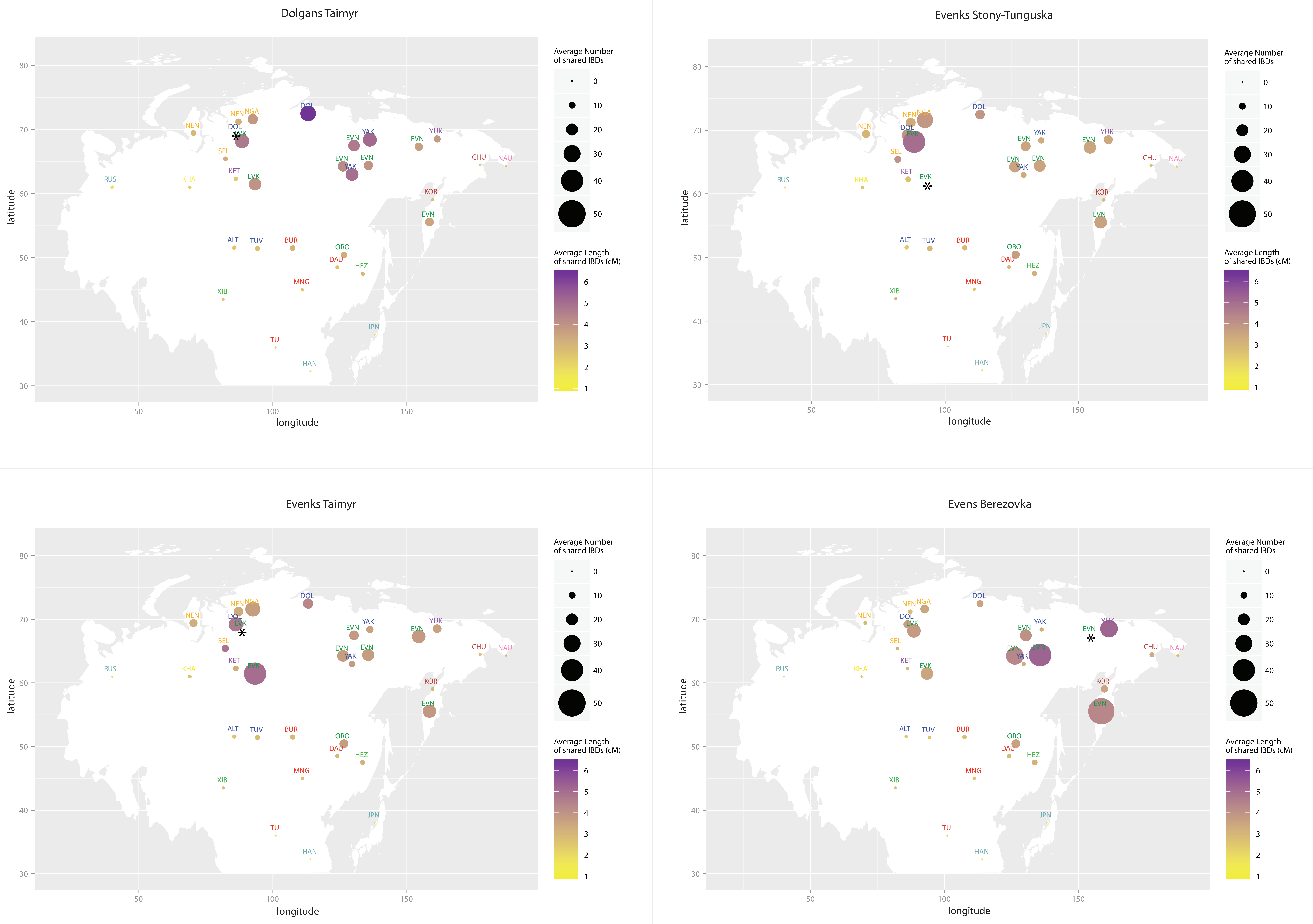

Dolgans Taimyr
Evenks Stony-Tunguska
Evenks Taimyr
Evens Berezovka
latitude
longitude
80
70
60
50
40
30
50
100
150
Average Number of shared IBDs
0
10
20
30
40
50
Average Length of shared IBDs (cM)
6
5
4
3
2
1
NEN
NGA
DOL
EVK
YAK
EVN
YUK
SEL
CHU
NAU
KET
RUS
KHA
KOR
ALT
TUV
BUR
ORO
DAU
HEZ
MNG
XIB
JPN
TU
HAN

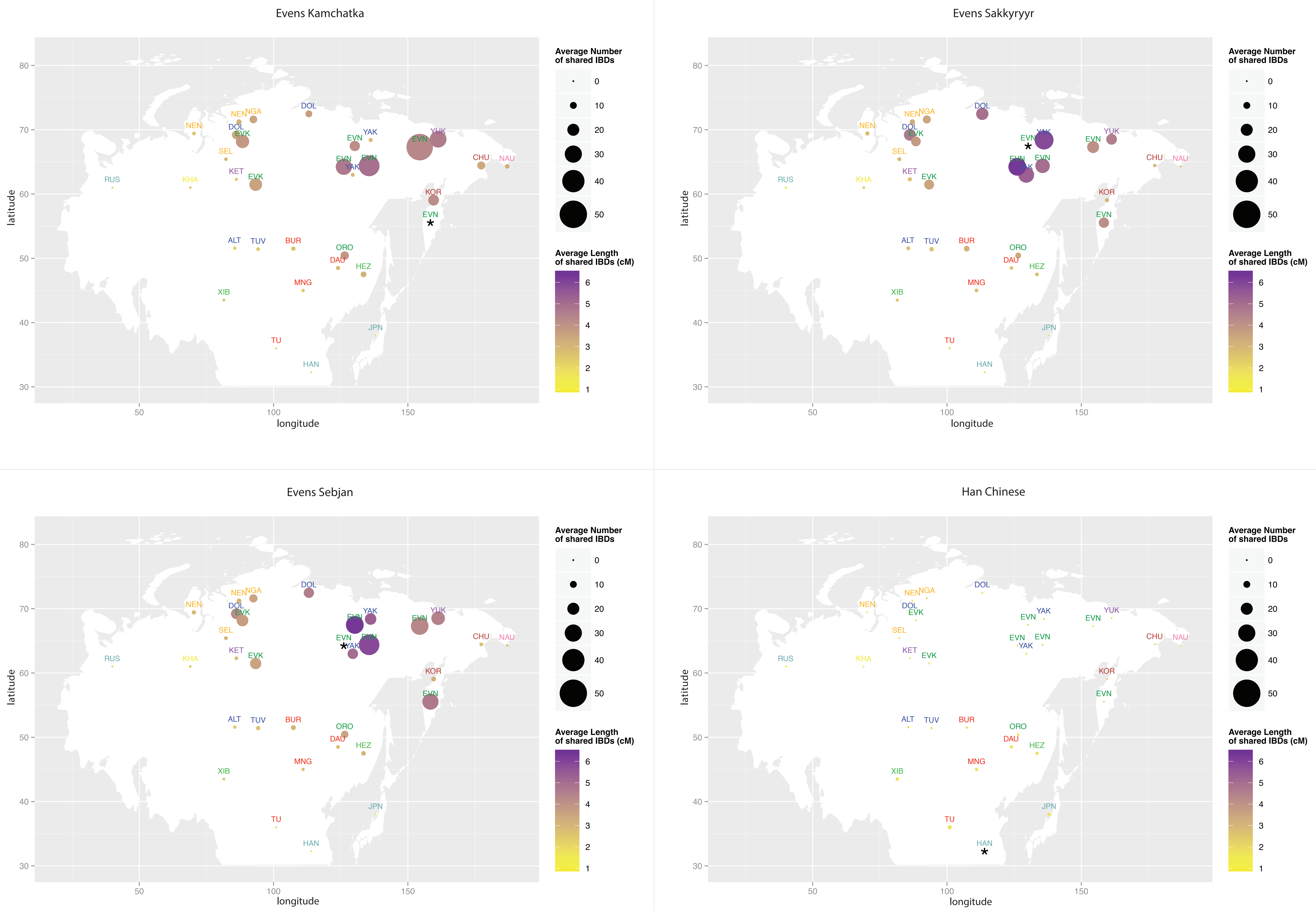

Evens Kamchatka
Evens Sakkyryyr
Evens Sebjan
Han Chinese
latitude
longitude
80
70
60
50
40
30
50
100
150
Average Number of shared IBDs
0
10
20
30
40
50
Average Length of shared IBDs (cM)
6
5
4
3
2
1
RUS
KHA
NEN
NGA
DOL
EVK
SEL
KET
ALT
TUV
BUR
XIB
MNG
TU
HAN
JPN
ORO
DAU
HEZ
EVN
YAK
YUK
CHU
NAU
KOR
*

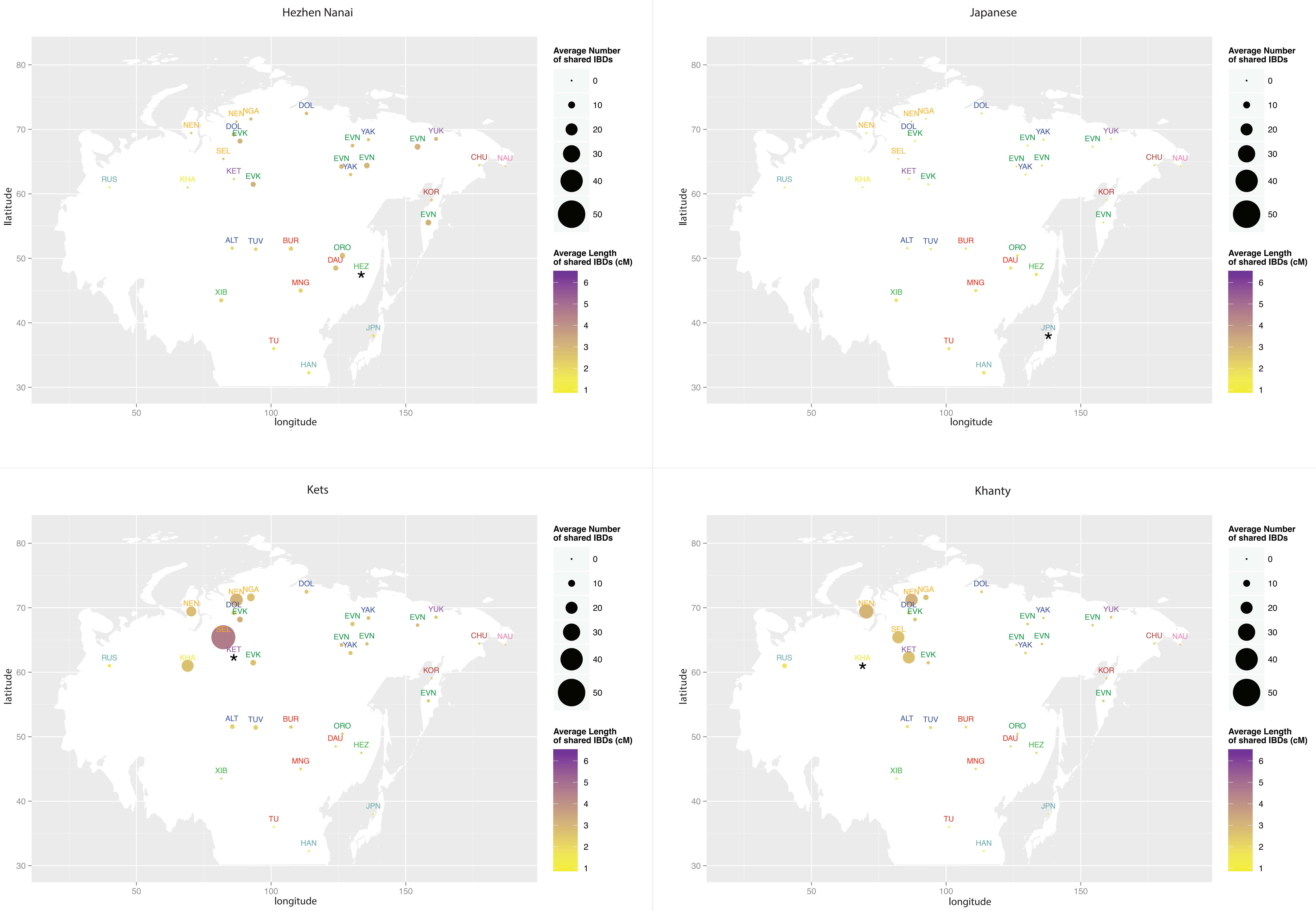
Hezhen Nanai
Japanese
Kets
Khanty
latitude
longitude
80
70
60
50
40
30
50
100
150
NEN
NGA
DOL
EVK
SEL
KET
RUS
KHA
EVN
YAK
YUK
CHU
NAU
KOR
ALT
TUV
BUR
ORO
DAU
HEZ
XIB
MNG
JPN
TU
HAN
Average Number of shared IBDs
0
10
20
30
40
50
Average Length of shared IBDs (cM)
6
5
4
3
2
1

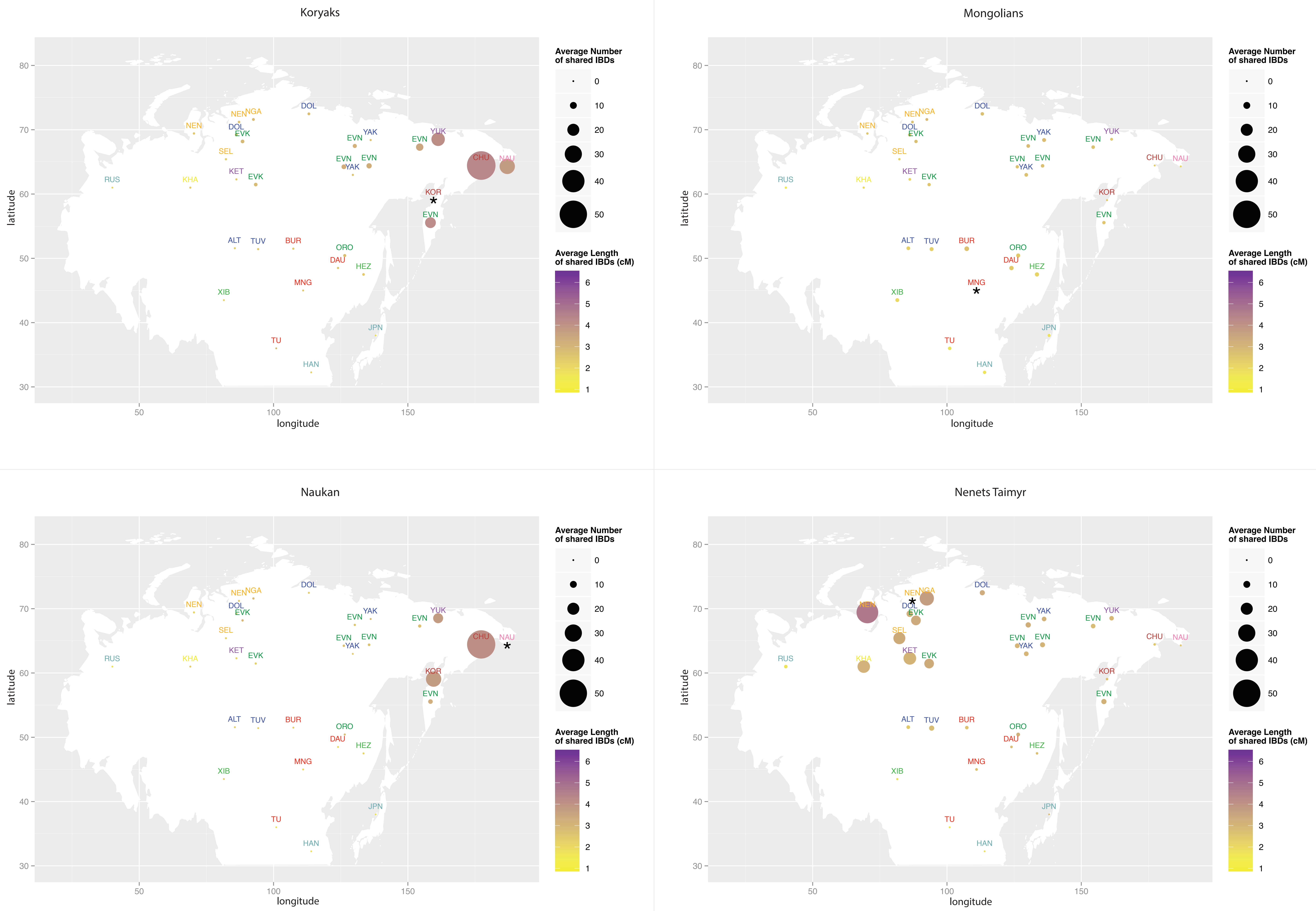
Koryaks
Mongolians
Naukan
Nenets Taimyr
latitude
longitude
80
70
60
50
40
30
50
100
150
Average Number of shared IBDs
0
10
20
30
40
50
Average Length of shared IBDs (cM)
6
5
4
3
2
1
RUS
KHA
NEN
NGA
DOL
EVK
SEL
KET
EVN
YAK
YUK
CHU
NAU
KOR
ALT
TUV
BUR
ORO
DAU
HEZ
XIB
MNG
TU
HAN
JPN

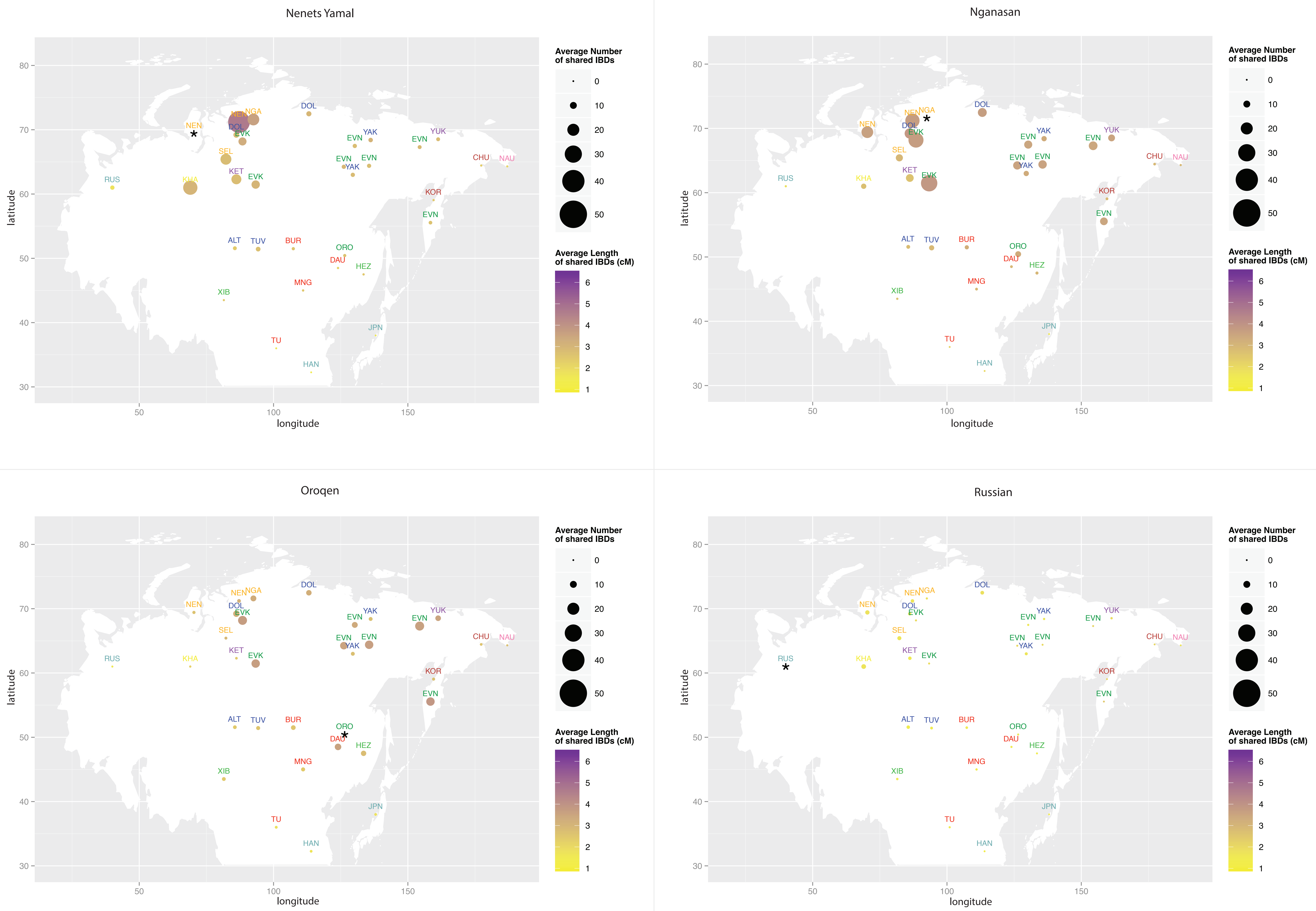
Nenets Yamal
Nganasan
Oroqen
Russian
latitude
longitude
80
70
60
50
40
30
50
100
150
Average Number of shared IBDs
0
10
20
30
40
50
Average Length of shared IBDs (cM)
6
5
4
3
2
1
RUS
KHA
NEN
NGA
DOL
EVK
SEL
KET
EVN
YAK
YUK
CHU
NAU
KOR
ALT
TUV
BUR
ORO
DAU
HEZ
XIB
MNG
JPN
TU
HAN
*

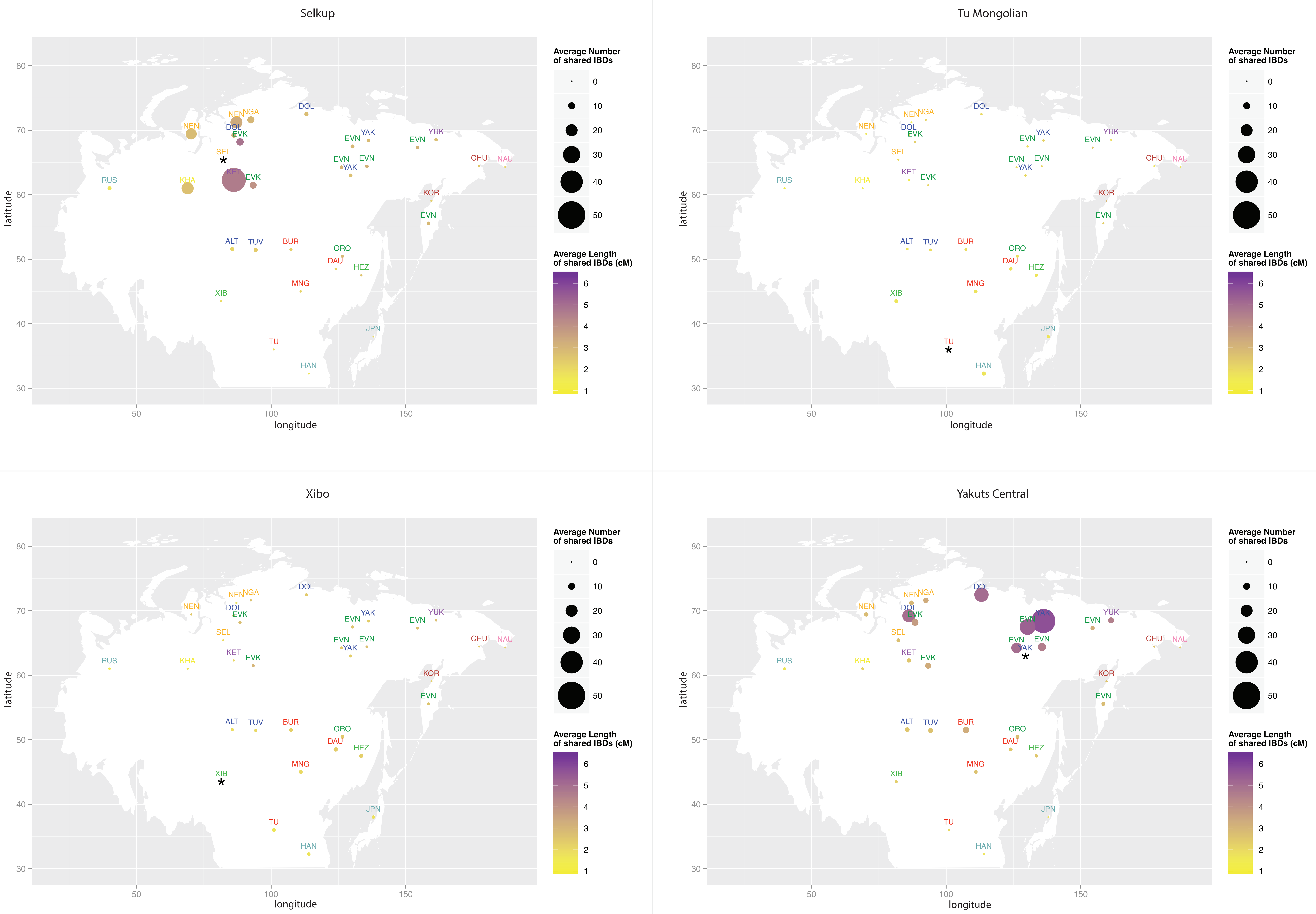
Selkup
Tu Mongolian
Xibo
Yakuts Central
latitude
longitude
80
70
60
50
40
30
50
100
150
Average Number of shared IBDs
0
10
20
30
40
50
Average Length of shared IBDs (cM)
6
5
4
3
2
1
RUS
KHA
NEN
NGA
DOL
EVK
SEL
KET
EVN
YAK
YUK
CHU
NAU
KOR
ALT
TUV
BUR
ORO
DAU
HEZ
MNG
XIB
TU
JPN
HAN

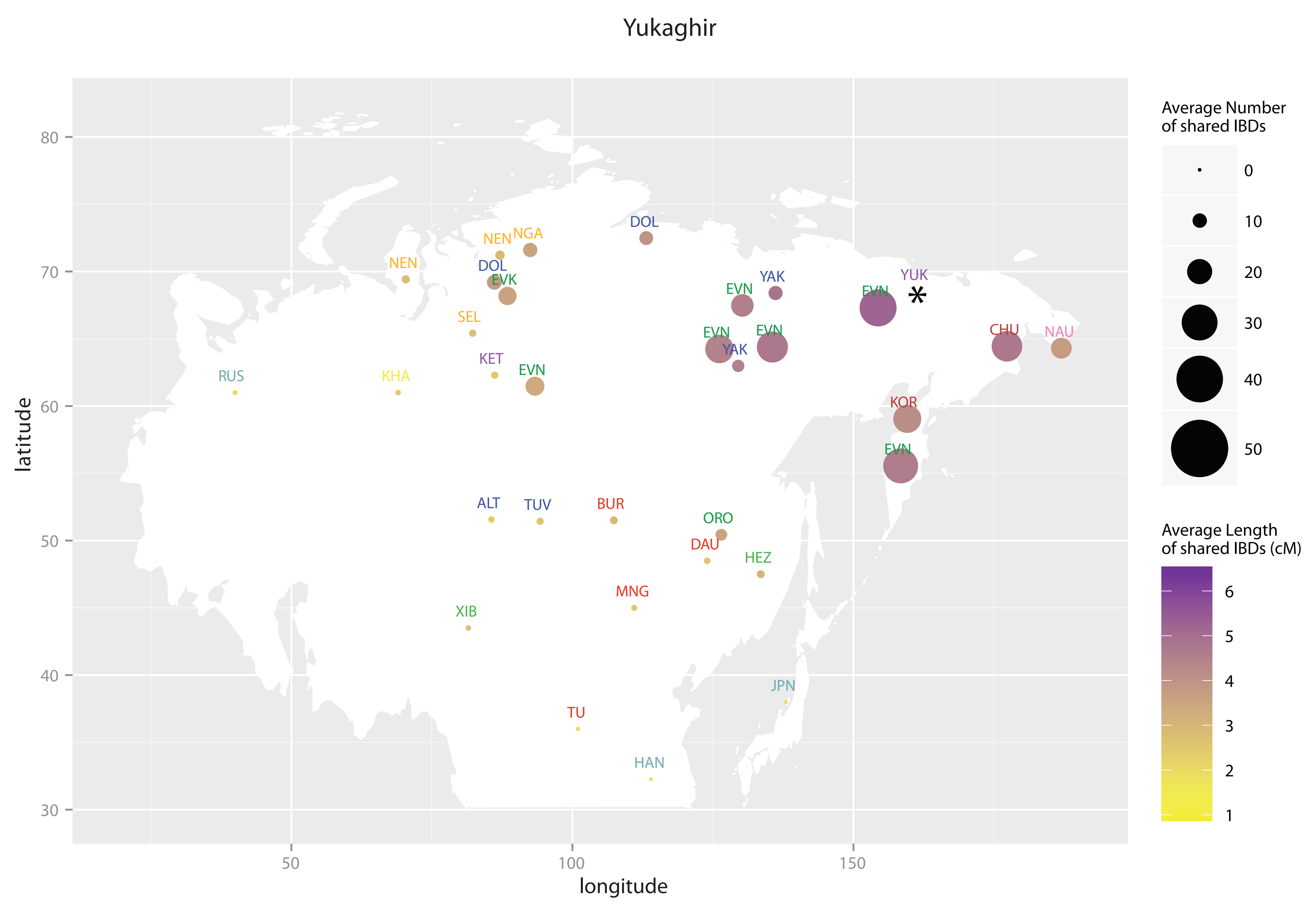

FIGURE S12. Recent relatedness based on IBD blocks. Populations are arranged in alphabetical order. Each data point represents the results for the comparison of the population marked with an asterisk to each of the other populations in the dataset. Data points are placed on the map according to the sampling location of each population (geographic coordinates are listed in Table S1). Population labels are abbreviated to the first 3 letters of the population name, except EVN = Even, EVK = Evenk, MNG = Mongolian, JPN = Japanese. Each label is color coded according to the population's linguistic affiliation as described in the legend to Figure 1 of the main text. The size of each circle is proportional to the mean number of IBD segments shared between the population marked with an asterisk and the population named in the label. The color intensity is proportional to the mean length of such shared IBD segments.

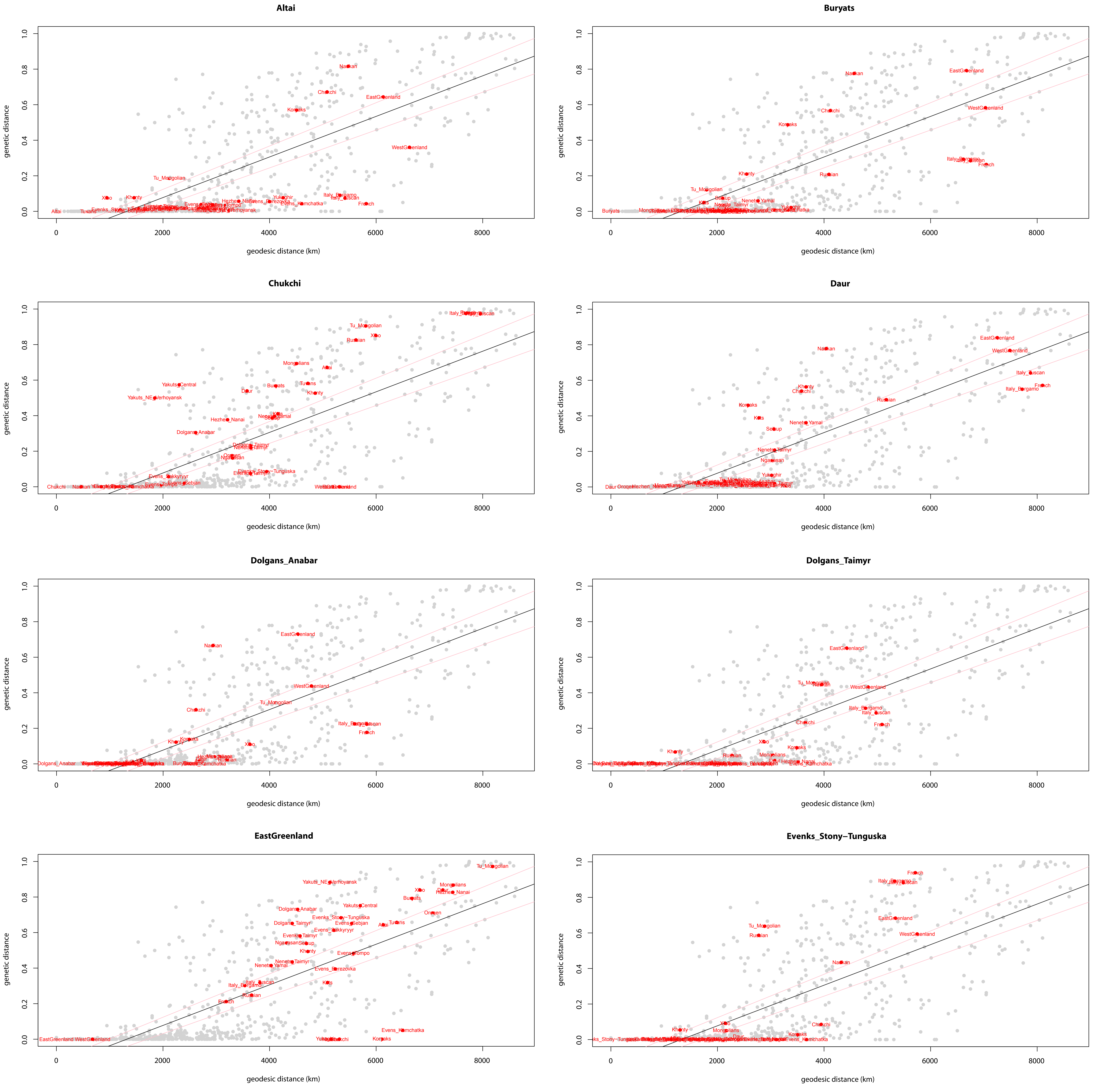
Altai
genetic distance
geodesic distance (km)
0
2000
4000
6000
8000
0.0
0.2
0.4
0.6
0.8
1.0
Naukan
Chukchi
EastGreenland
Koryaks
WestGreenland
Tu_Mongolian
Xibo
Khanty
Yukaghir
Italy_Bergamo
Italy_Tuscan
French
Evens_Kamchatka
Altai
Tuvans
Buryats
Genetic distance plotted against geodesic distance (km) for the following groups:
Chukchi
Naukan
EastGreenland
WestGreenland
Italy_Bergamo
Italy_Tuscan
French
Russian
Khanty
Tu_Mongolian
Nenets_Yamal
Yukaghir
Evens_Kamchatka
Buryats
Mongolians
Tu_Mongolian
Xibo
Russian
Mongolians
Altai
Yakuts_Central
Buryats
Tuvans
Daur
Khanty
Yakuts_NE_Verhoyansk
Hezhen_Nanai
Dolgans_Anabar
Dolgans_Taimyr
Nenets_Taimyr
Ngansan
Evens_Sakkyryyr
Evens_Gebjan
Evens_Tompo
Evenks_Stony_Tunguska
Chukchi
Naukan
Daur
EastGreenland
Naukan
WestGreenland
Italy_Tuscan
French
Italy_Bergamo
Khanty
Chukchi
Koryaks
Russian
Kets
Nenets_Yamal
Selkup
Nenets_Taimyr
Nganasan
Yukaghir
Daur
Oroqen
Hezhen_Nanai
Dolgans_Anabar
EastGreenland
Naukan
WestGreenland
Tu_Mongolian
Chukchi
Italy_Bergamo
Italy_Tuscan
French
Khanty
Koryaks
Xibo
Hezhen_Nanai
Mongolians
Russian
Dolgans_Anabar
Buryats
Evens_Kamchatka
Dolgans_Taimyr
EastGreenland
Tu_Mongolian
Naukan
WestGreenland
Italy_Bergamo
Italy_Tuscan
Chukchi
French
Xibo
Koryaks
Khanty
Russian
Mongolians
Hezhen_Nanai
Evens_Kamchatka
EastGreenland
Tu_Mongolian
Yakuts_NE_Verhoyansk
Xibo
Mongolians
Daur
Hezhen_Nanai
Buryats
Yakuts_Central
Dolgans_Anabar
Oroqen
Evenks_Stony_Tunguska
Dolgans_Taimyr
Evens_Gebjan
Altai
Tuvans
Evens_Sakkyryyr
Evenks_Taimyr
Nganasan
Selkup
Khanty
Evens_Tompo
Nenets_Taimyr
Nenets_Yamal
Evens_Berezovka
Kets
Italy_Tuscan
Italy_Bergamo
Russian
French
Evens_Kamchatka
Koryaks
EastGreenland
WestGreenland
Evenks_Stony_Tunguska
French
Italy_Bergamo
Italy_Tuscan
Tu_Mongolian
Russian
EastGreenland
WestGreenland
Naukan
Xibo
Khanty
Mongolians
Chukchi
Koryaks
Daur
Evens_Kamchatka

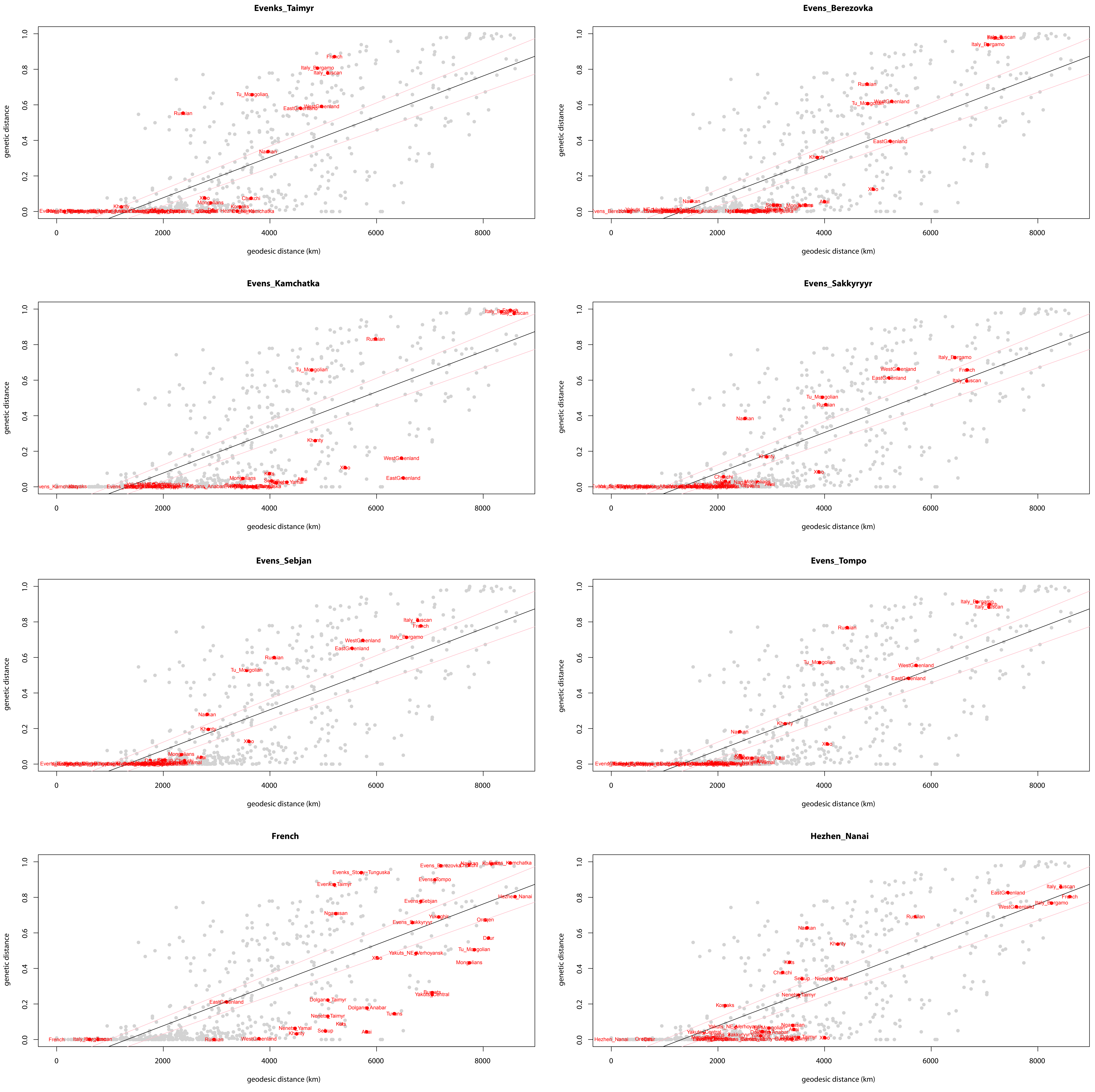

Evenks_Taimyr
Evens_Berezovka
Evens_Kamchatka
Evens_Sakkyryyr
Evens_Sebjan
Evens_Tompo
French
Hezhen_Nanai
genetic distance
geodesic distance (km)
0
2000
4000
6000
8000
0.0
0.2
0.4
0.6
0.8
1.0
French
Italy_Bergamo
Italy_Tuscan
Tu_Mongolian
Russian
WestGreenland
EastGreenland
Nganasan
Khanty
Chukchi
Mongolians
Koryaks
Kets
Selkup
Altai
Tuvans
Yakuts_NE_Verhoyansk
Yakuts_Central
Dolgans_Taimyr
Dolgans_Anabar
Nenets_Taimyr
Nenets_Yamal
Evens_Kamchatka
Evens_Berezovka
Evens_Tompo
Evens_Sebjan
Evens_Sakkyryyr
Evenks_Taimyr
Evenks_Stony_Tunguska
Yukaghir
Oroqen
Daur
Buryats
Hezhen_Nanai

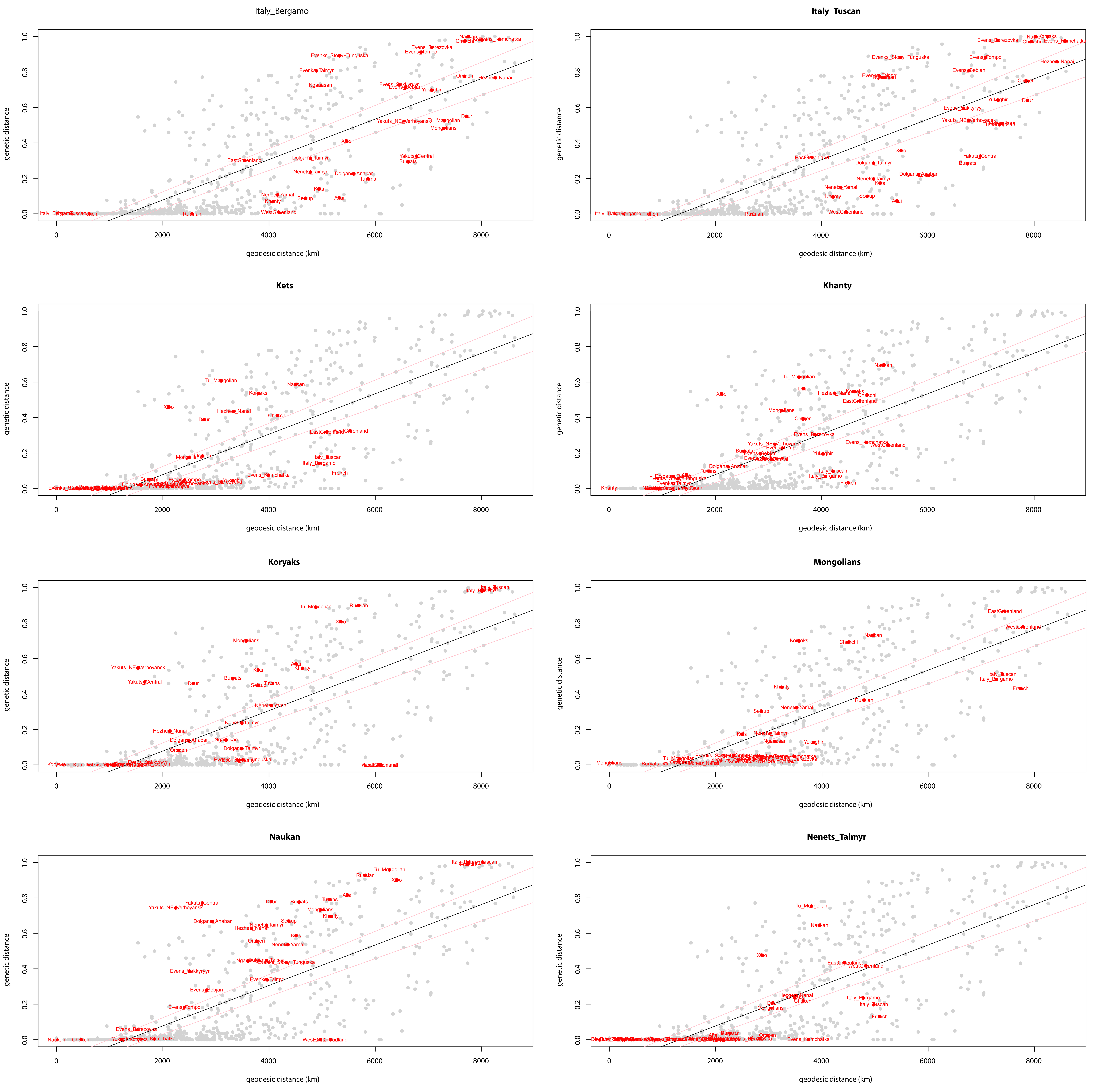
Italy_Bergamo
Italy_Tuscan
Kets
Khanty
Koryaks
Mongolians
Naukan
Nenets_Taimyr
genetic distance
geodesic distance (km)

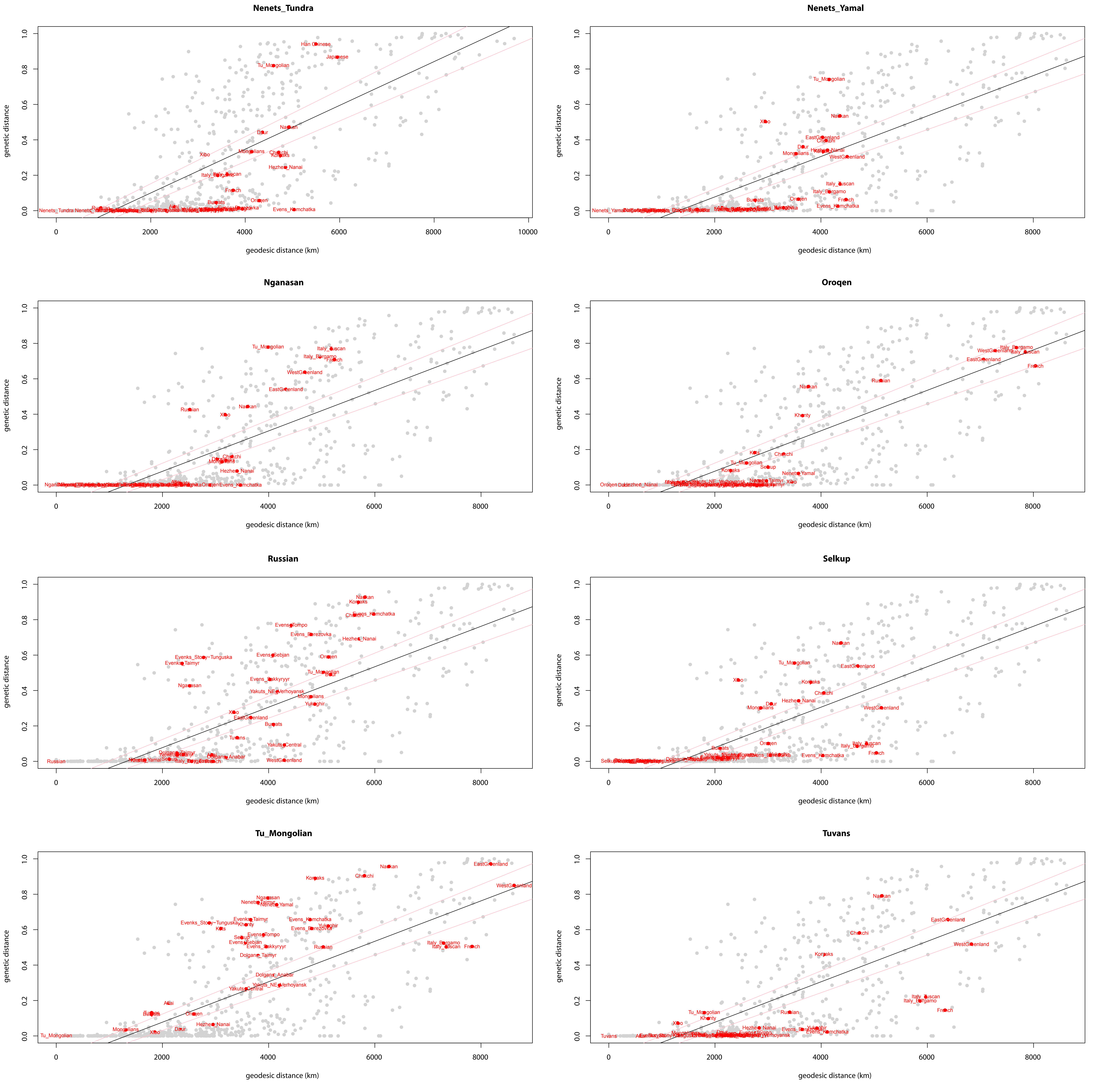
Nenets_Tundra
Han Chinese
Japanese
Tu_Mongolian
Nanai
Daur
Xibo
Mongolians
Chukchi
Koryaks
Hezhen_Nanai
Italy_Tuscan
French
Oroqen
Buryats
Evens_Kamchatka
Nenets_Tundra
Nenets_Yamal
Nganasan
Tu_Mongolian
Xibo
EastGreenland
Chukchi
Daur
Mongolians
WestGreenland
Italy_Tuscan
Italy_Bergamo
Buryats
Oroqen
French
Evens_Kamchatka
Nenets_Yamal
Oroqen
Italy_Bergamo
WestGreenland
Italy_Tuscan
EastGreenland
French
Russian
Nanai
Khanty
Kets
Chukchi
Tu_Mongolian
Selkup
Koryaks
Nenets_Yamal
Xibo
Oroqen
Hezhen_Nanai
Russian
Nanai
Koryaks
Chukchi
Evens_Kamchatka
Evens_Tompo
Evens_Berezovka
Hezhen_Nanai
Evenks_Stony−Tunguska
Evens_Sebjan
Oroqen
Evenks_Taimyr
Tu_Mongolian
Nganasan
Evens_Sakkyryyr
Yakuts_NE_Verhoyansk
Mongolians
Yukaghir
Xibo
EastGreenland
Buryats
Tuvans
Yakuts_Central
Dolgans_Anabar
WestGreenland
Russian
Selkup
Nanai
Tu_Mongolian
EastGreenland
Xibo
Koryaks
Chukchi
Hezhen_Nanai
Daur
Mongolians
WestGreenland
Oroqen
Italy_Tuscan
Italy_Bergamo
French
Buryats
Evens_Kamchatka
Selkup
Tu_Mongolian
EastGreenland
Nanai
Koryaks
Chukchi
Nganasan
Nenets_Tundra
Nenets_Yamal
WestGreenland
Evenks_Stony−Tunguska
Evenks_Taimyr
Evens_Kamchatka
Khanty
Kets
Evens_Berezovka
Yukaghir
Evens_Tompo
Selkup
Evens_Sebjan
Evens_Sakkyryyr
Russian
Italy_Bergamo
Italy_Tuscan
French
Dolgans_Taimyr
Dolgans_Anabar
Yakuts_Central
Yakuts_NE_Verhoyansk
Altai
Buryats
Oroqen
Hezhen_Nanai
Mongolians
Xibo
Daur
Tu_Mongolian
Tuvans
Nanai
EastGreenland
Chukchi
WestGreenland
Koryaks
Italy_Tuscan
Italy_Bergamo
French
Tu_Mongolian
Xibo
Khanty
Russian
Hezhen_Nanai
Evens_Kamchatka
Yukaghir
Tuvans
genetic distance
geodesic distance (km)
0.0
0.2
0.4
0.6
0.8
1.0
0
2000
4000
6000
8000
10000

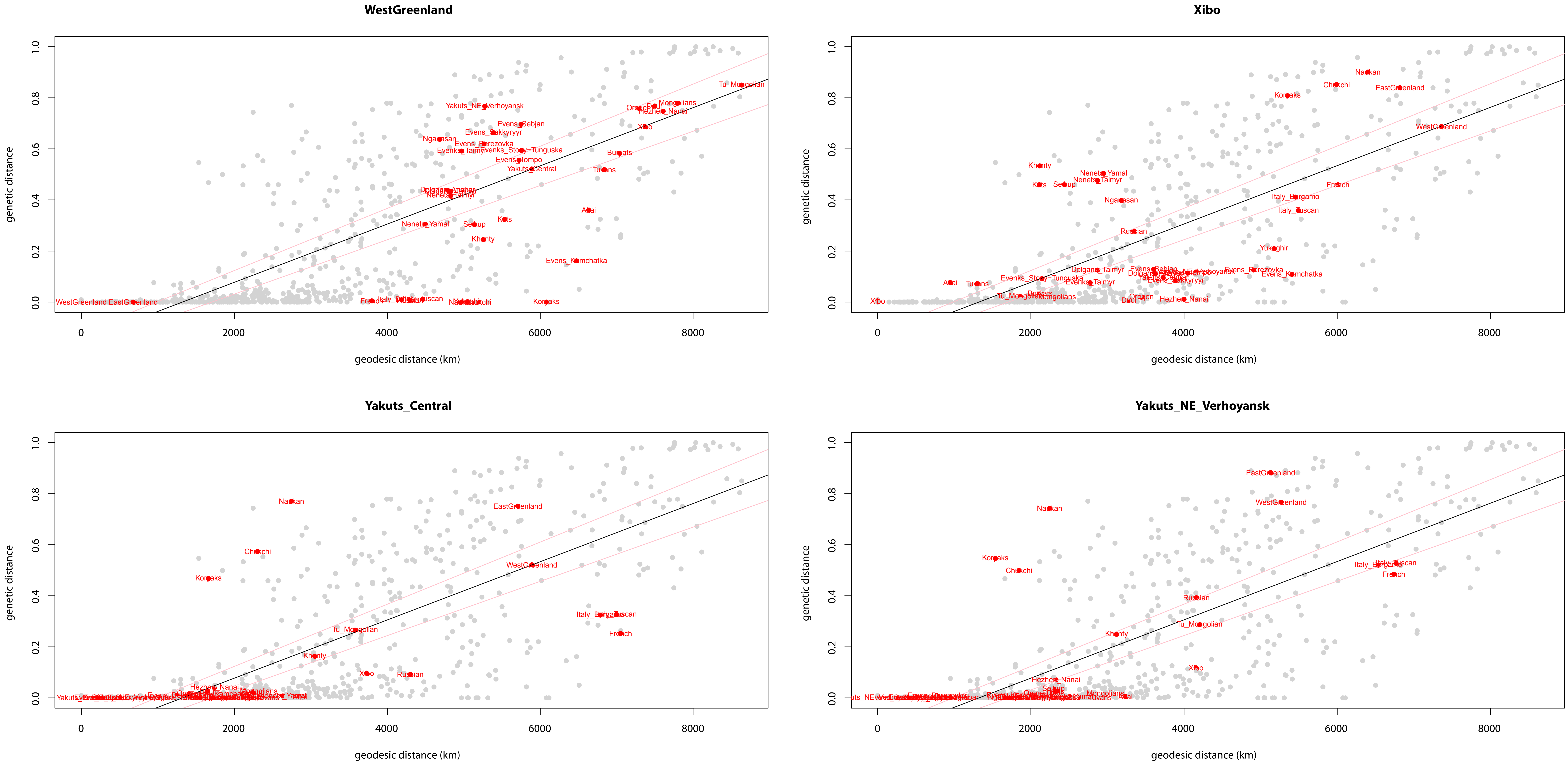

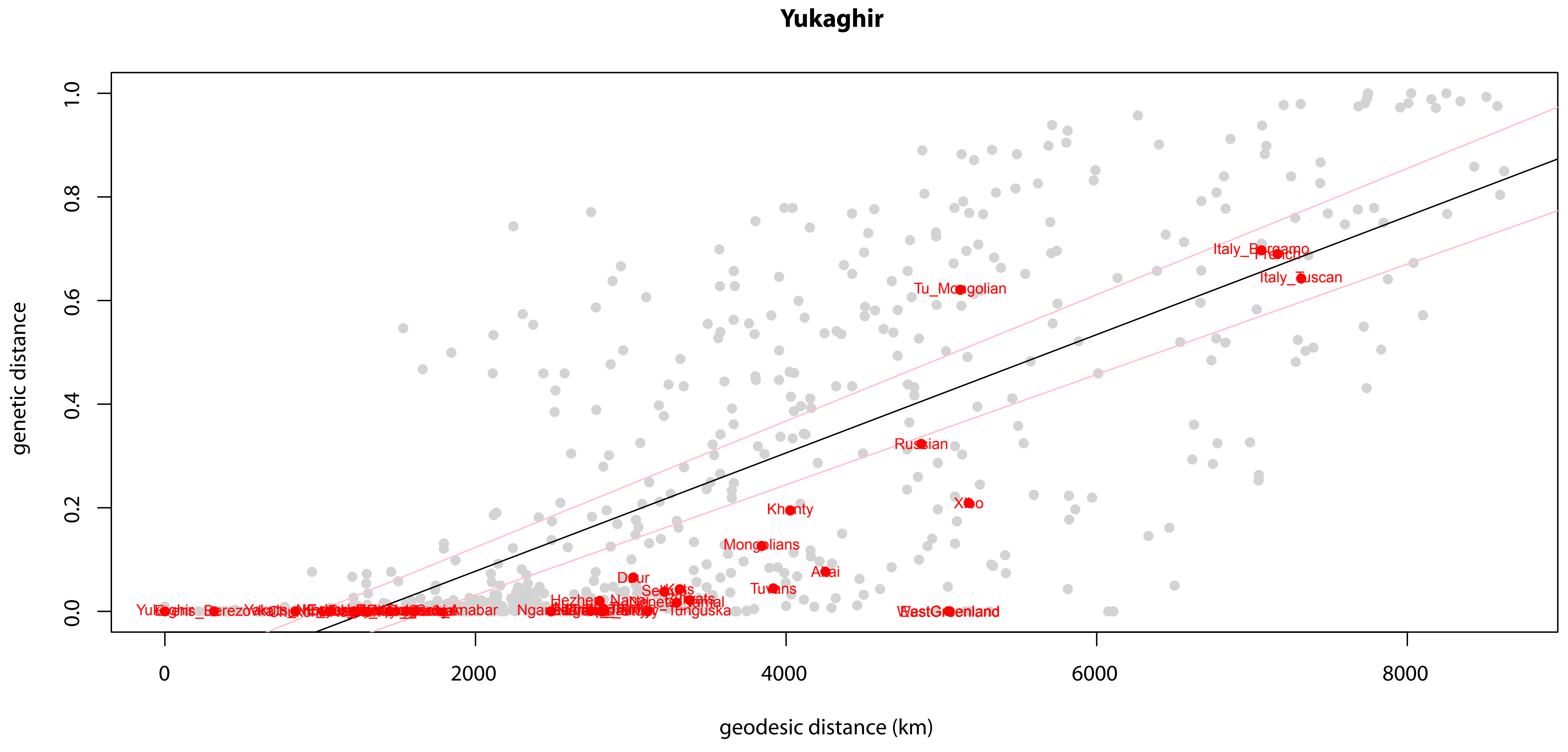

FIGURE S13. Relationship between genetic and geographic distances. Genetic distances were calculated based on the inverse of the IBD-sharing matrix. The regression line is drawn in black and the associated 95% confidence interval in red. In each plot red dots denote pairwise comparisons between the population indicated in the plot's title and every other population, while all other data points are in grey.

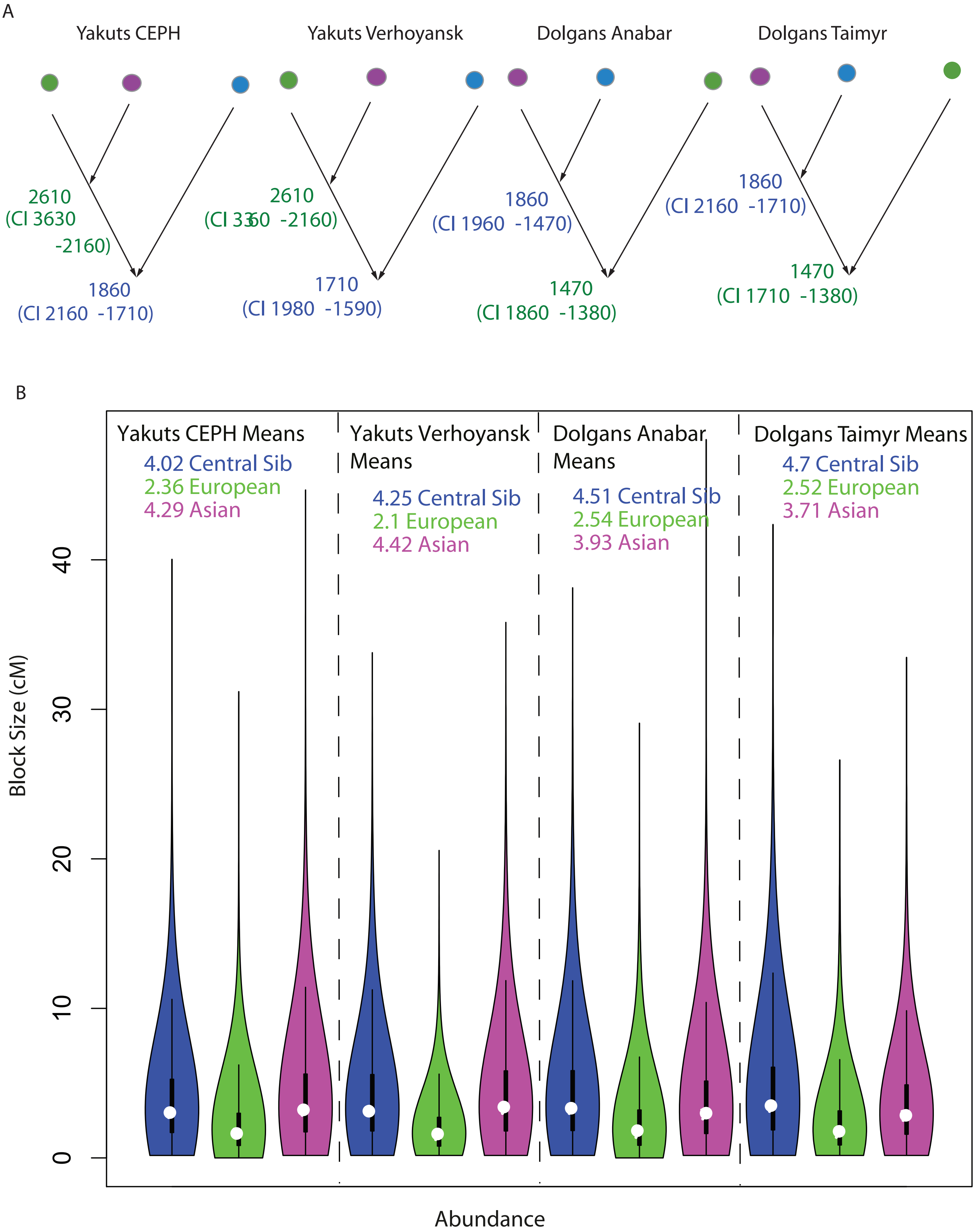

FIGURE S14. Admixture profiles for the northern Siberian Turkic-speaking populations: Yakuts and Dolgans. (A) Admixture history graphs and admixture dates inferred for each population and each admixture episode. Proxy parental populations for the different ancestral components (represented as circles) were as follows: Light Green = Italians, Blue = Nganasan, Pink = Han Chinese. (B) Cumulative distribution of all ancestry blocks. For each population the plot captures the total abundance of blocks of each ancestry (x axis) of different genetic lengths in cM (y axis); the average width of the blocks of each ancestry and variance around the mean are also shown.

A

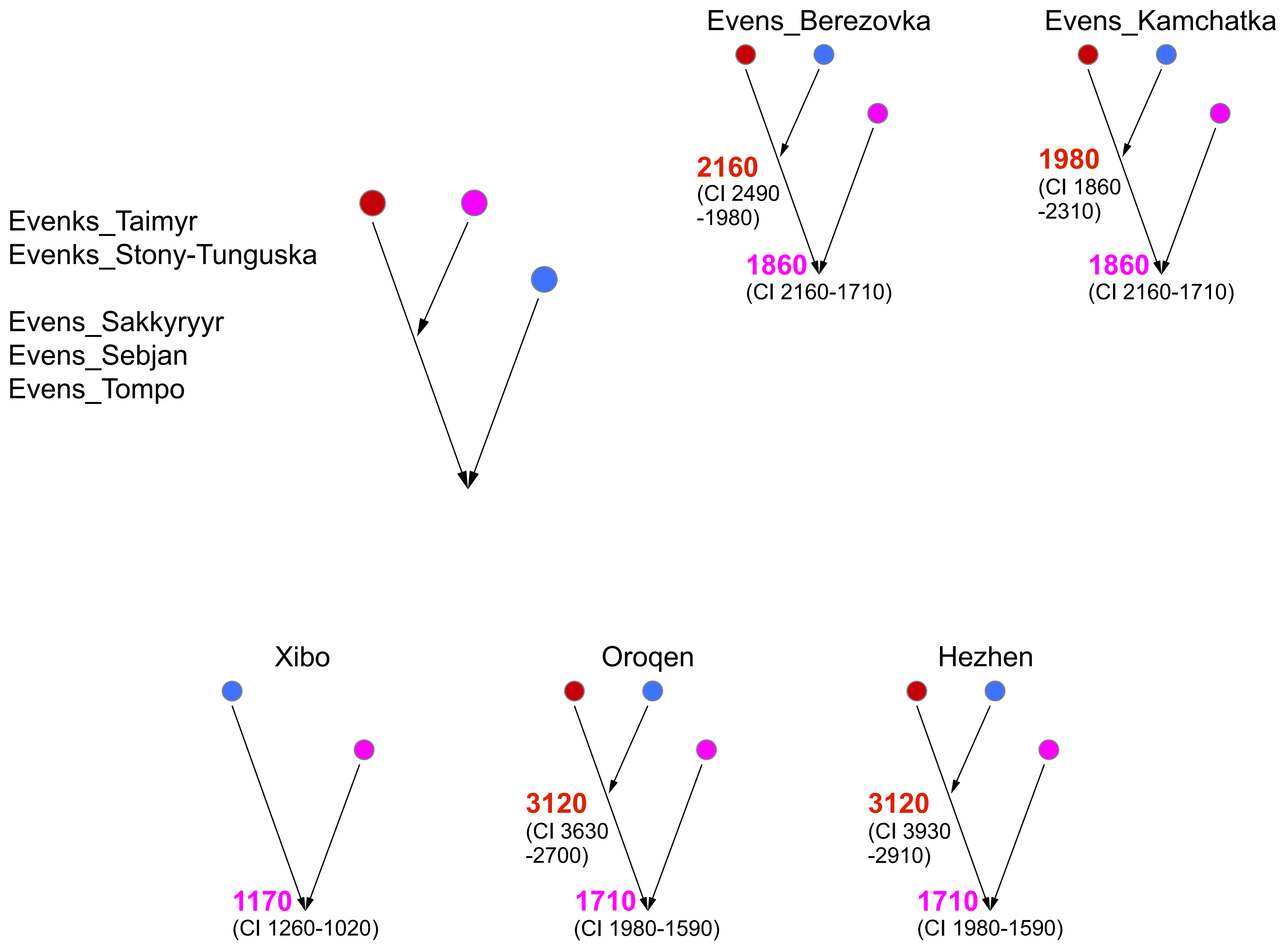

Evenks_Taimyr
Evenks_Stony-Tunguska
Evens_Sakkyryyr
Evens_Sebjan
Evens_Tompo
Evens_Berezovka
2160
(CI 2490 -1980)
1860
(CI 2160-1710)
Evens_Kamchatka
1980
(CI 1860 -2310)
1860
(CI 2160-1710)
Xibo
1170
(CI 1260-1020)
Oroqen
3120
(CI 3630 -2700)
1710
(CI 1980-1590)
Hezhen
3120
(CI 3930 -2910)
1710
(CI 1980-1590)

B

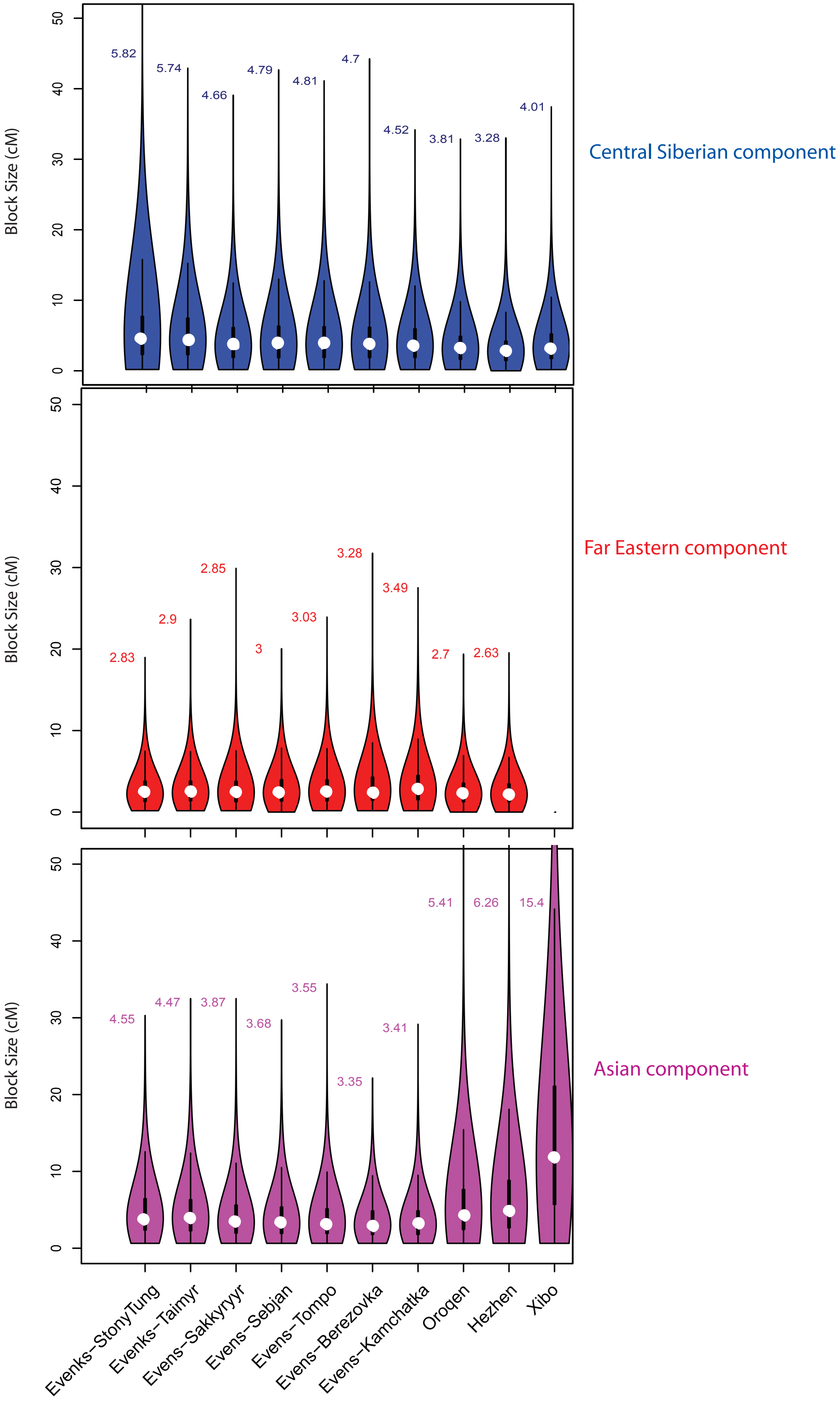

FIGURE S15. Admixture profiles for the Tungusic-speaking populations: Evenks, Evens, Oroqen, Hezhen and Xibo. (A) Admixture history graphs and admixture dates inferred for each population and each admixture episode. Proxy parental populations for the different ancestral components (represented as circles) were as follows: Blue = Nganasan, Red = Koryaks, Pink = Han Chinese. (B) Cumulative distribution of all ancestry blocks. For each population the plot captures the total abundance of blocks of each ancestry (x axis) of different genetic lengths in cM (y axis); the average width of the blocks of each ancestry and variance around the mean are also shown.

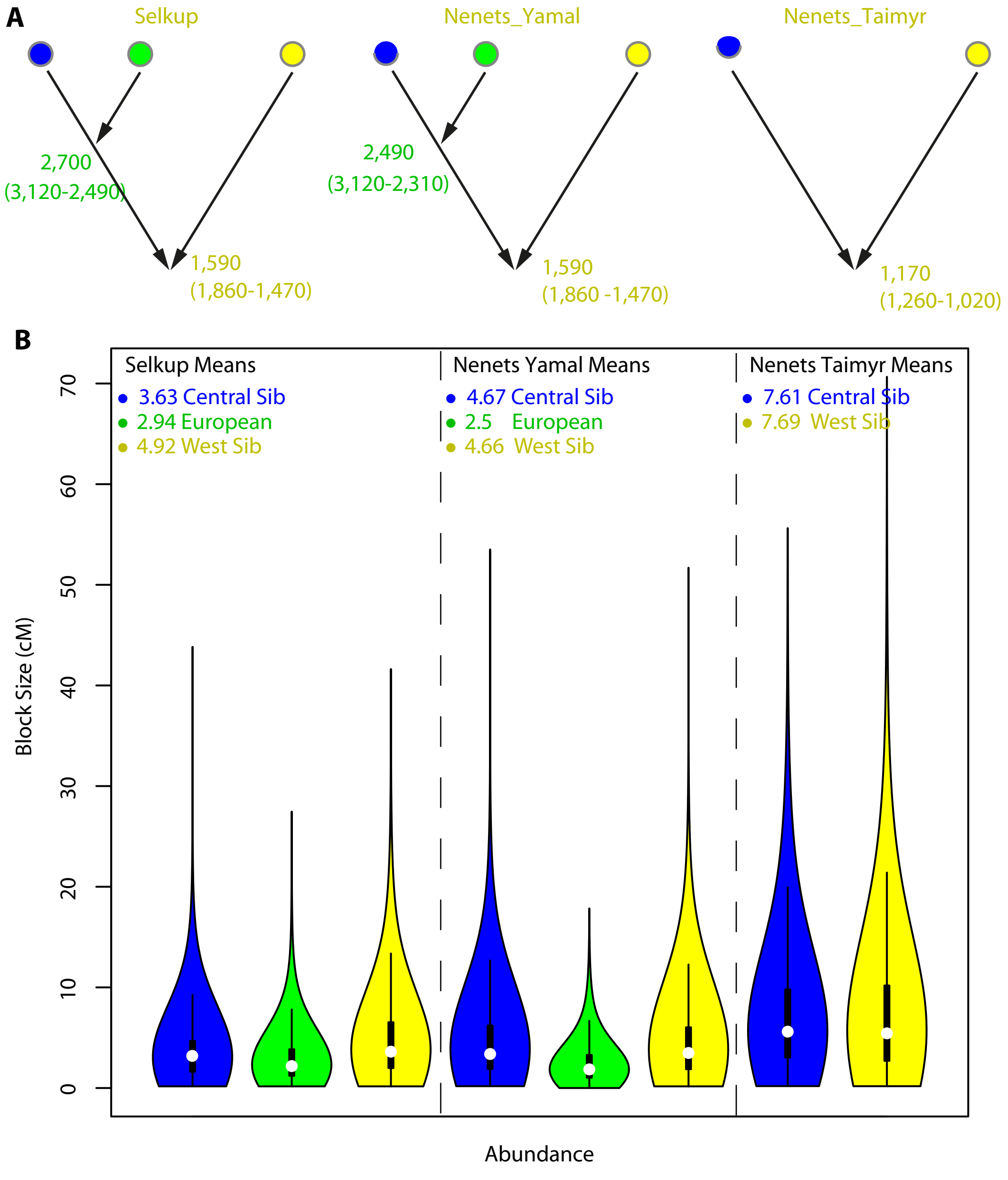

FIGURE S16. Admixture profiles for the Samoyedic-speaking populations: Selkup and Nenets. (A) Admixture history graphs and admixture dates inferred for each population and each admixture episode. Proxy parental populations for the different ancestral components (represented as circles) were as follows: Blue = Nganasan, Yellow = Khanty, Light Green = Italians. (B) Cumulative distribution of all ancestry blocks. For each population the plot captures the total abundance of blocks of each ancestry (x axis) of different genetic lengths in cM (y axis); the average width of the blocks of each ancestry and variance around the mean are also shown.

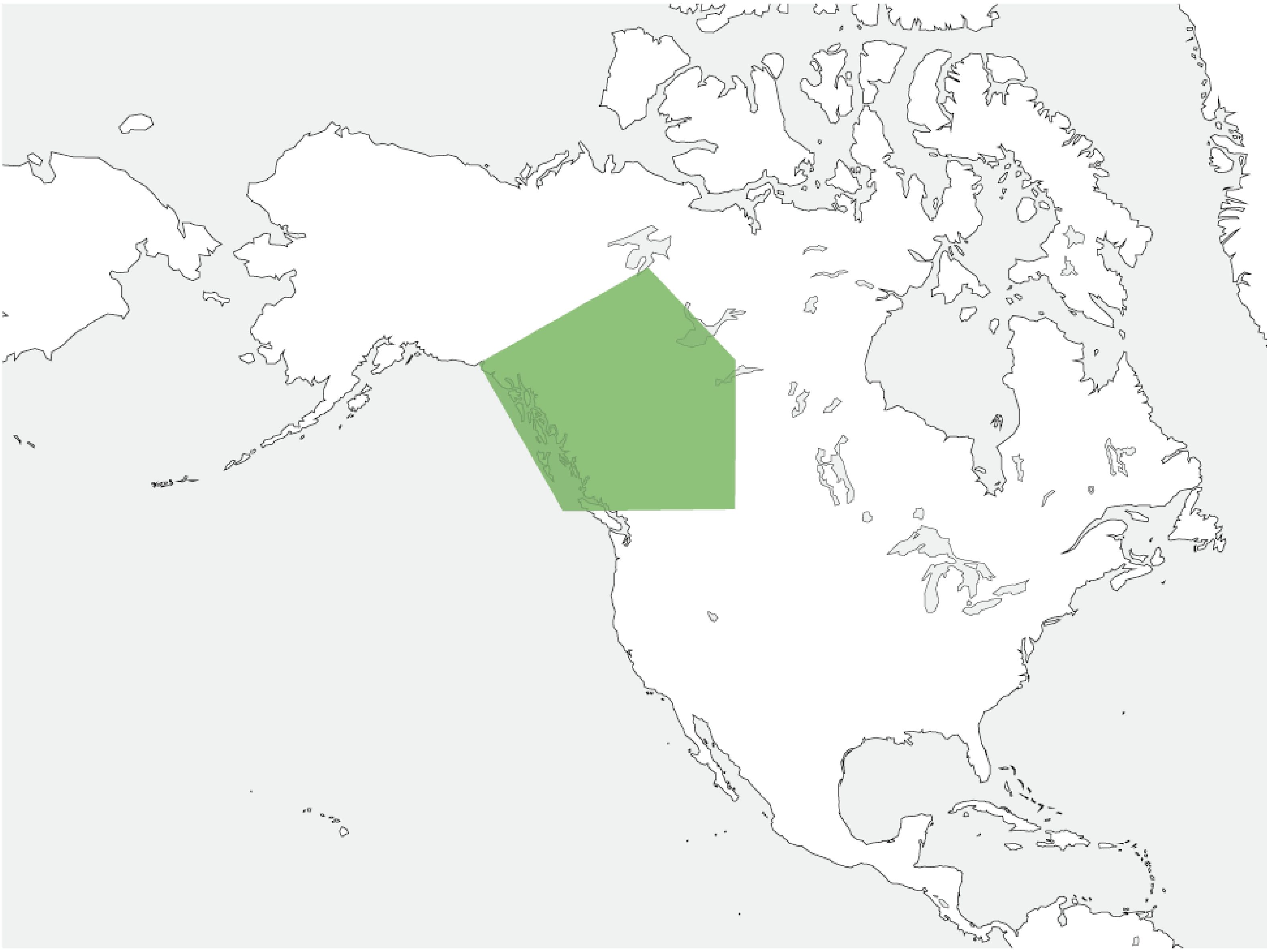

FIGURE S17. Extrapolated origins of the Siberian Naukan Yupik. The extrapolation is based on the results of the regression analysis of the genetic and geographic distances (Figure S22), which revealed that all the genetic distances between the Naukan Yupik and other populations in Siberia are elevated given the geographic distance between them. In order to improve the fit between the genetics and geography, the Naukan Yupik were assigned different sets of geographic coordinates in Alaska, Canada and Greenland, each time recalculating the regression coefficient. The best improvement was achieved when the Naukan Yupik were assigned any set of coordinates in the geographic region of Canada circumscribed here by the shaded area.

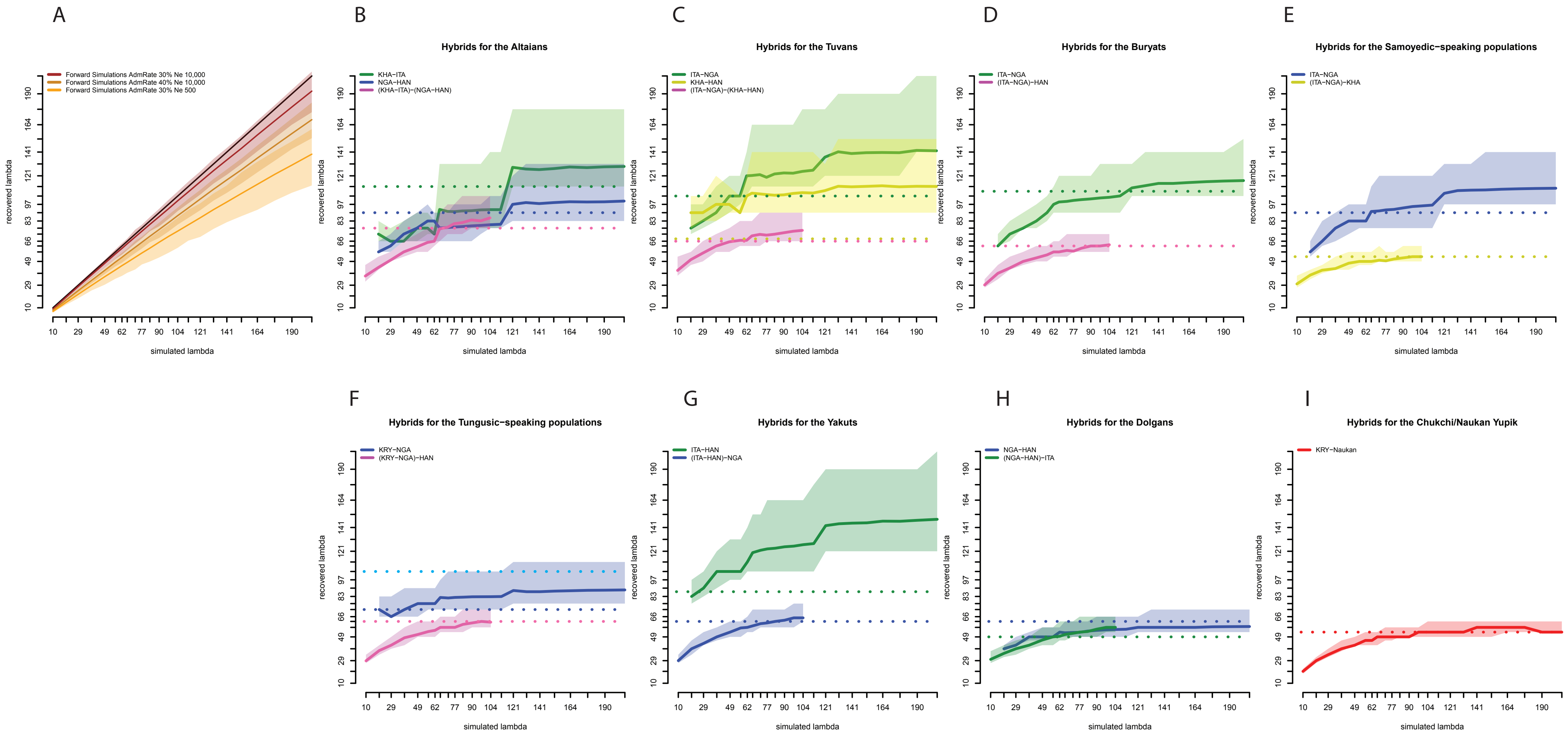

FIGURE S18. Assessment of the time-depth resolution of the study. (A) Admixture dates recovered from the results of forward simulations (100 simulations for each parameter setting) where we model dynamics of recombination in an admixed population with a certain rate of admixture and Ne. The observed number of breakpoints starts to deviate from the expected value (parameterized by λ) beginning around 50-100 generations since the admixture event (see Text S1D for details and references). Accordingly, since the WT method recovers not the simulated number of breakpoints, but the time of admixture the observed number of breakpoints corresponds to, the recovered λ deviates from the simulated λ. (B-I) Recovery of the admixture dates from artificial genomes, generated from populations used in this study as proxies for the parental groups. Solid lines show recovered admixture dates in an artificially generated hybrid population where a more recent gene flow occurs on a background of an older admixture event. The shaded area denotes the 95% confidence interval. Dotted lines denote the date of admixture actually inferred from the Siberian data. In (F) the lighter blue dotted line indicates the date of the Red-Blue admixture inferred for the Oroqen, while the darker blue line indicates the date inferred for the Berezovka Evens.

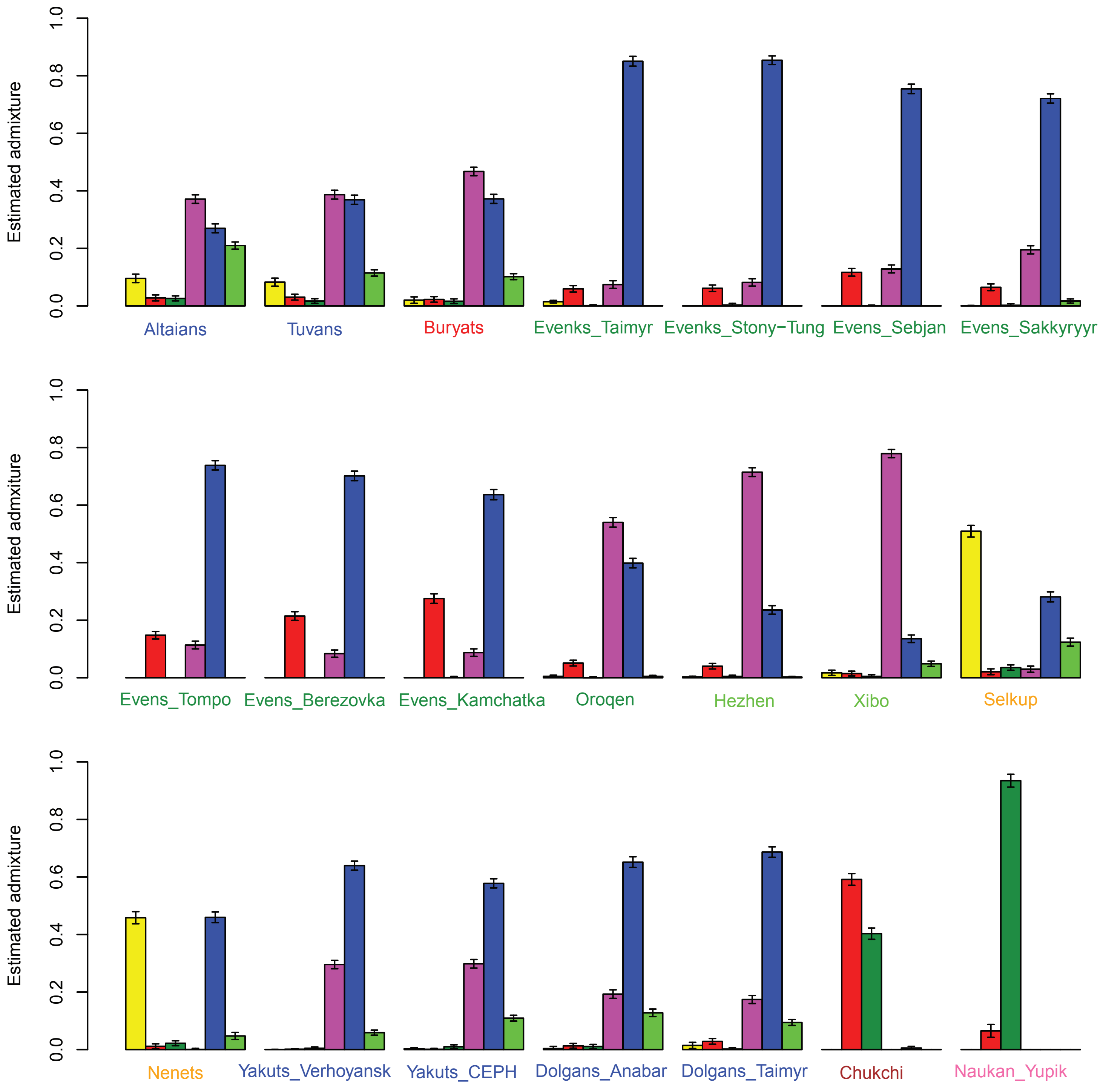

FIGURE S19. Admixture proportions and standard errors determined for each estimated admixture coefficient using a block bootstrap approach. Each color indicates a different ancestry component as described in the legend to Figure 1 of the main text.

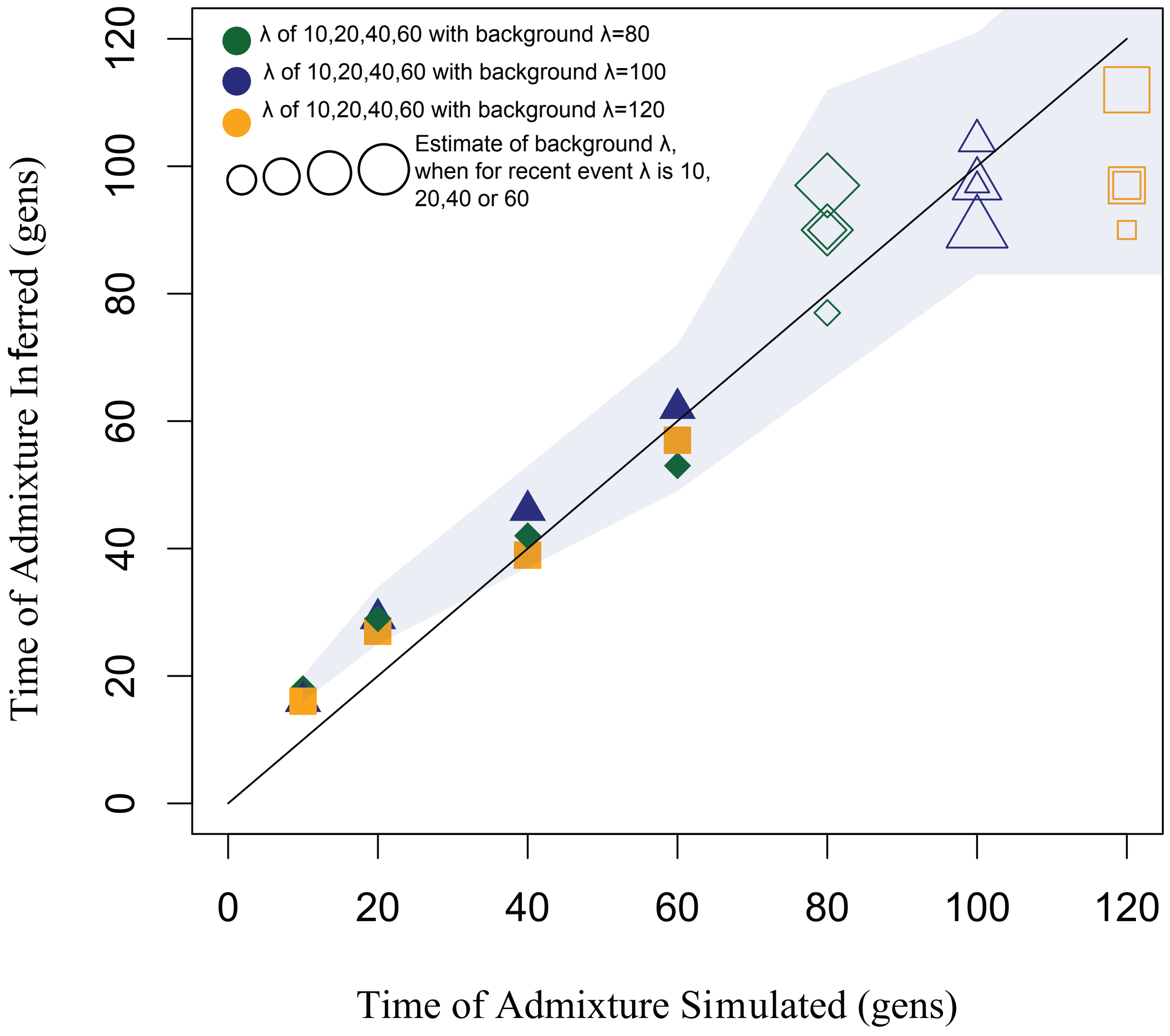

FIGURE S20. Performance of the wavelet transform analysis (adapted to include complex admixture scenarios) in recovering the simulated dates of admixture. The method was applied to 12 artificial datasets with different admixture histories. Each recent admixture time point (λ = 10, 20, 40 and 60) was simulated three times, each time using a different admixture background (λ = 80, 100 or 120). The shaded area denotes the 95% confidence interval for the inferred dates of admixture.

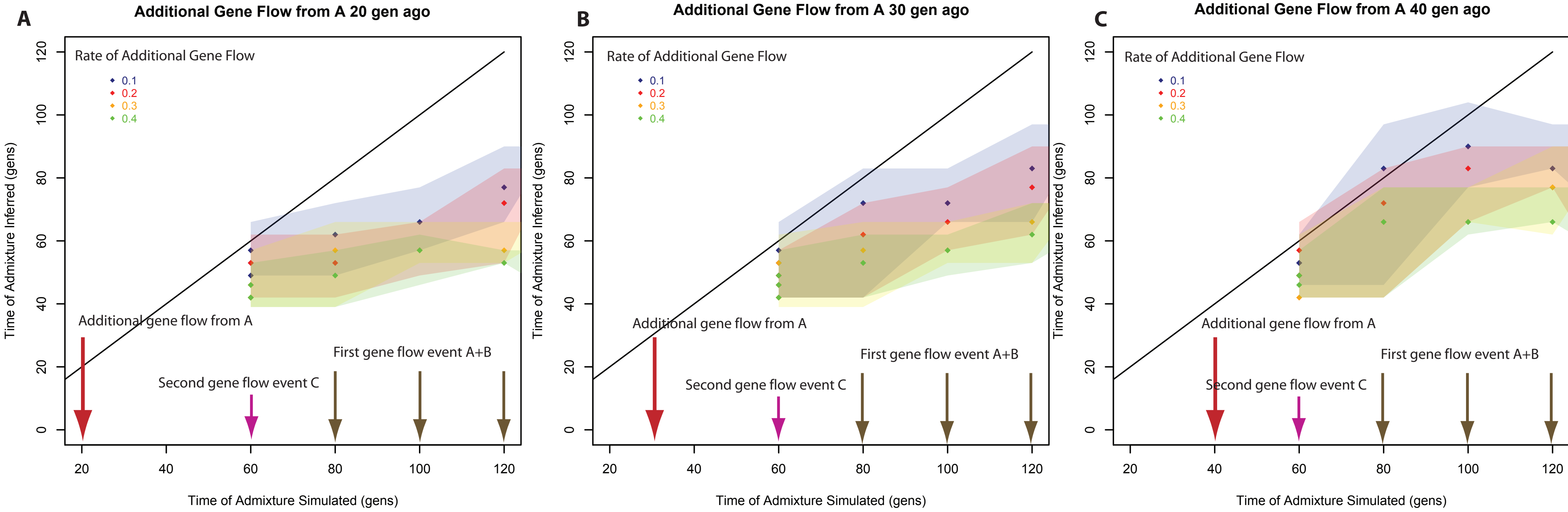

FIGURE S21. Admixture date estimates for artificially constructed admixed populations, where admixture history involved one ancestry which was contributed twice. Each panel shows a comparison of simulated vs. estimated dates of admixture for 12 sets of simulated populations (each with the same recent episode of admixture 60 generations ago, and a different earlier episode of admixture 120, 100 or 80 generations ago). Additional gene flow with four different admixture rates ($\alpha = 0.1, 0.2, 0.3$ or 0.4) from a population which had already contributed to an earlier episode was simulated for each of these populations. The shaded area denotes the 95% confidence interval for the inferred dates of admixture. Additional gene flow was introduced at (A) 20 (B) 30 or (C) 40 generations ago.

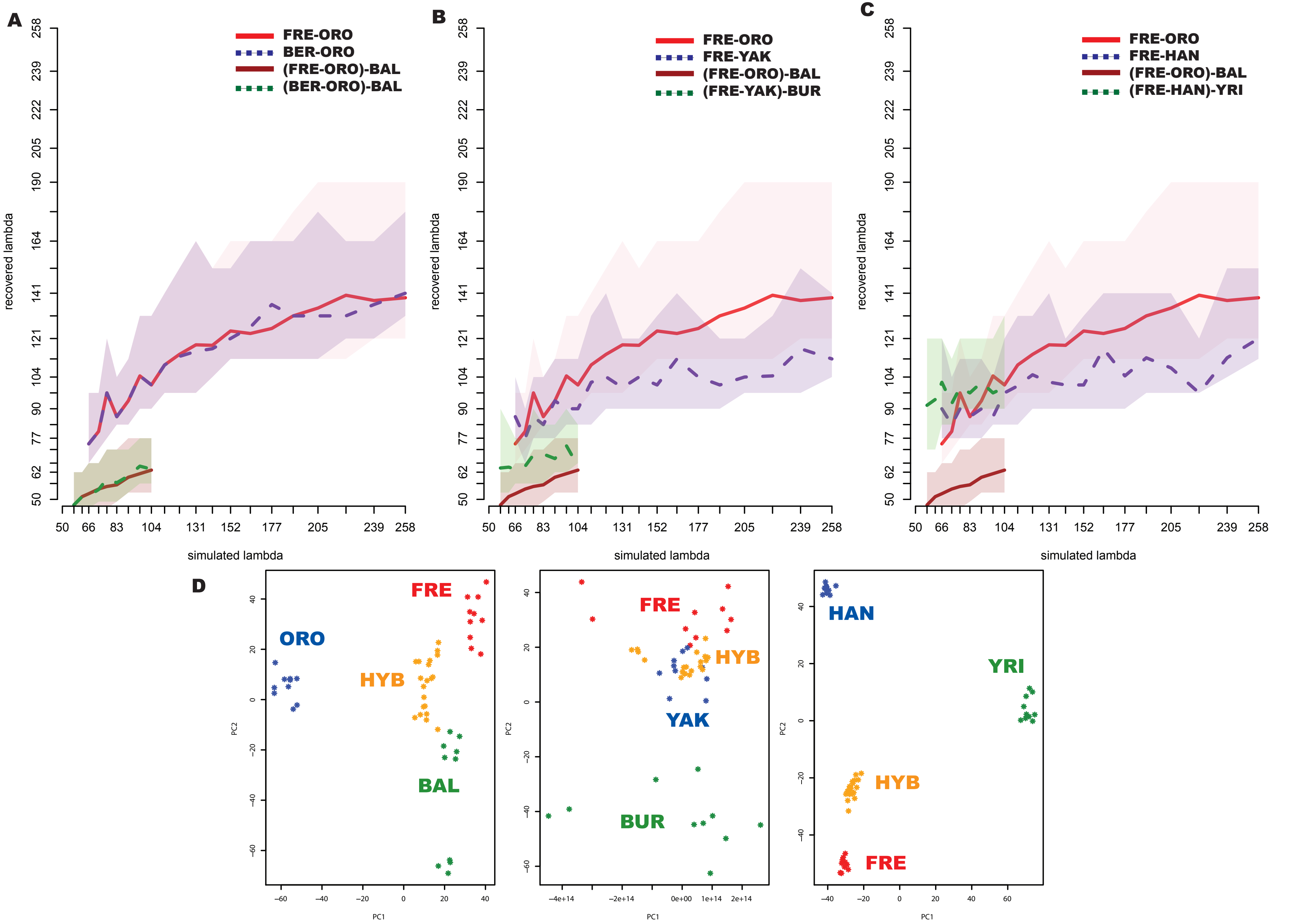

FIGURE S22. Sensitivity of PCAdmix and WT methods to mis-specification of parental groups. (A-C) Recovery of the admixture dates from artificial genomes. Solid lines show the result when the inference of ancestry along the chromsome by PCAdmix is based on the true source populations. Dotted lines show results obtained when mis-specified proxies are used in lieu of the true sources. (D) PCA results for SNPs used in the analysis.

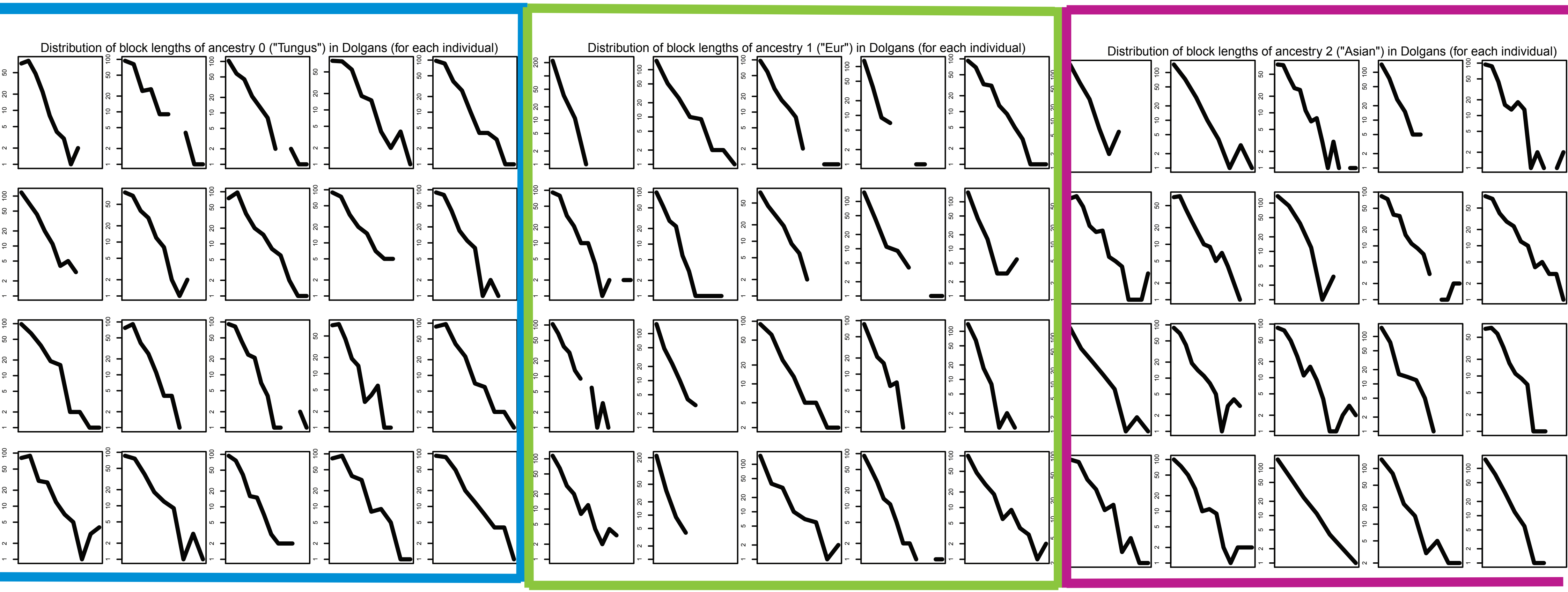

FIGURE S23. Test for excess of small-sized ancestry blocks as inferred by PCAdmix, with the expectation that an excess of short ancestral blocks would indicate erroneous insertions by PCAdmix. Shown is the genomewide distribution of block widths of each ancestry inferred for each Anabar Dolgan individual. Block width in (cM) is shown on the x-axis, while abundance is represented on the y-axis (note that the scale here is logarithmic).

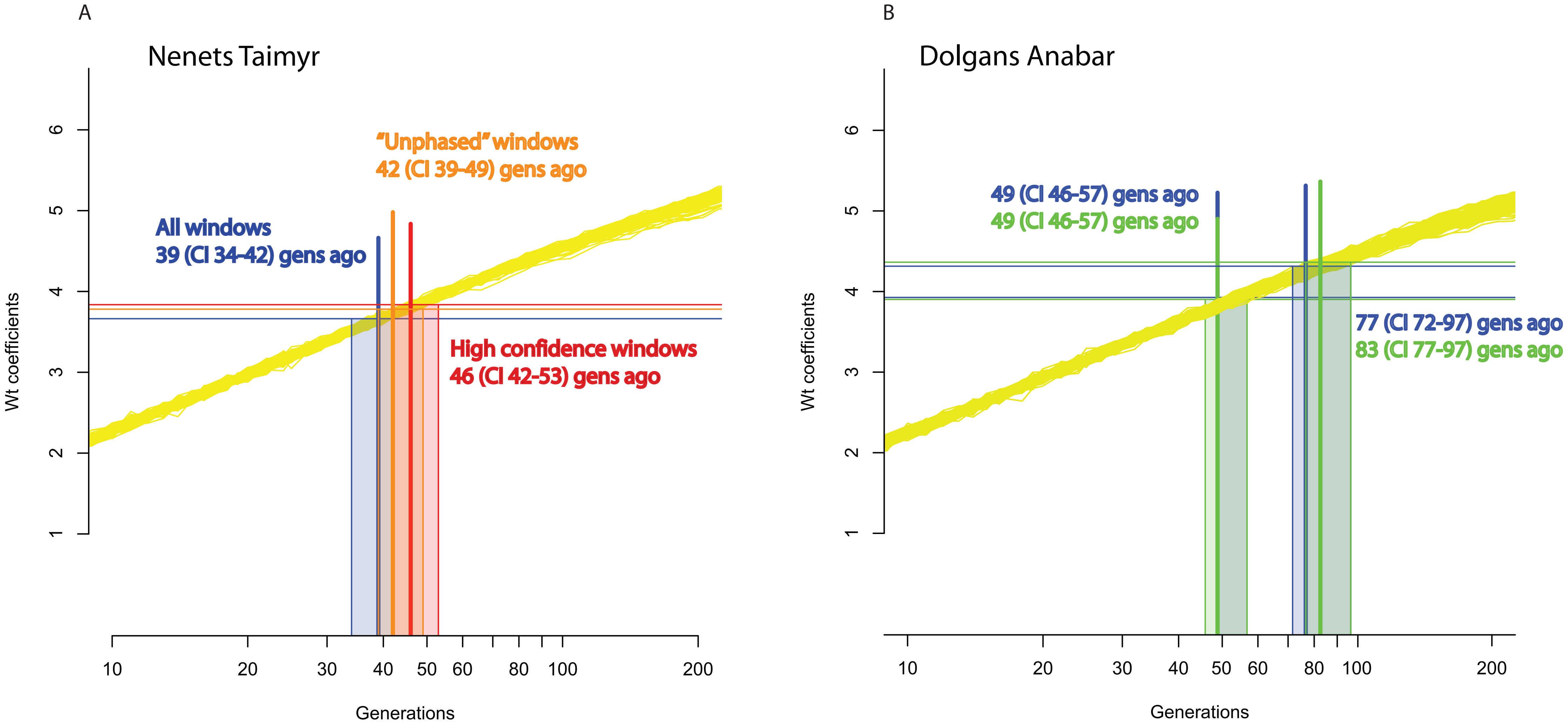

FIGURE S24. Testing the effect of the phasing switch error on time of admixture estimates. (A). Comparison of dates of admixture inferred using a full set of windows (blue) vs. only high confidence windows (red), and on artificially "unphased" data (orange). Dating is based on simulated data from 100 simulations with a 30% migration rate. Each curve represents a single admixed population. Average WT centers calculated for 100 chromosomes drawn at random from each population at exponentially growing time points are plotted as a function of time. Vertical lines indicate the time estimate, and shaded boxes define the confidence intervals. Time estimates are based on the entire unfiltered sample of Taimyr Nenents, and the admixture rate for the simulated data is chosen so as to match the empirical data. (B). Admixture time estimates (for two episodes of admixture) based on data phased with either BEAGLE or SHAPEit. Measurements obtained for a more recent vs. an older admixture episode using the BEAGLE-phased and SHAPEit-phased data are shown by blue and green horizontal lines, respectively. Vertical lines indicate the time estimate (blue font for the BEAGLE-phased, and green font for the SHAPE-it phased data), and shaded boxes define the confidence intervals. Time estimates are based on the entire unfiltered sample of Anabar Dolgans, and the admixture rate for the simulated data is chosen so as to match the empirical data.
